# Application of the C3-Methyltransferase StspM1 for the Synthesis of the Natural Pyrroloindole Motif

**DOI:** 10.1021/acscatal.3c04952

**Published:** 2023-12-14

**Authors:** Mona Haase, Benoit David, Beatrix Paschold, Thomas Classen, Pascal Schneider, Nadiia Pozhydaieva, Holger Gohlke, Jörg Pietruszka

**Affiliations:** † Institute for Bioorganic Chemistry & Bioeconomy Science Center (BioSC), Heinrich Heine University Düsseldorf in Forschungszentrum Jülich, 52426 Jülich, Germany; ‡ Institute of Bio- and Geosciences (IBG-4: Bioinformatics) Forschungszentrum Jülich, 52426 Jülich, Germany; § Institute of Bio- and Geosciences (IBG-1: Bioorganic Chemistry) & Bioeconomy Science Center (BioSC), Forschungszentrum Jülich, 52426 Jülich, Germany; ∥ Institute for Pharmaceutical and Medicinal Chemistry & Bioeconomy Science Center (BioSC), Heinrich Heine University Düsseldorf, 40225 Düsseldorf, Germany

**Keywords:** methyltransferase, diketopiperazine, selective C-methylation, reaction mechanism, SAM recycling

## Abstract

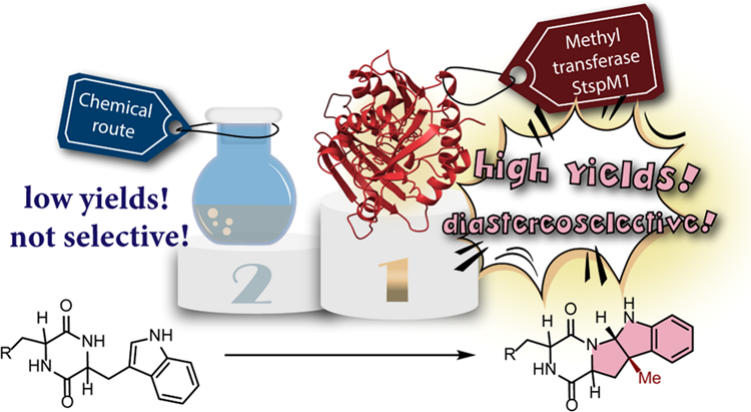

Even though pyrroloindoles are widely present in natural products with different kinds of biological activities, their selective synthesis remains challenging with existing tools in organic chemistry, and there is furthermore a demand for stereoselective and mild methods to access this structural motif. Nature uses C3-methyltransferases to form the pyrroloindole framework, starting from the amino acid tryptophan. In the present study, the SAM-dependent methyltransferase StspM1 from *Streptomyces* sp. HPH0547 is used to build the pyrroloindole structural motif in tryptophan-based diketopiperazines (DKP). The substrate scope of the enzyme regarding different Trp-Trp-DKP isomers was investigated on an experimental and computational level. After further characterization and optimization of the methylation reaction with a design of experiment approach, a preparative scale reaction with the immobilized enzyme including a SAM regeneration system was performed to show the synthetic use of this biocatalytic tool to access the pyrroloindole structural motif.

## Introduction

Many natural products containing a pyrroloindole structural motif exhibit different kinds of biological activities, such as antibacterial and anticancer effects.^1−4^ Despite its biological relevance, the stereoselective synthesis of the pyrroloindole structural motif (hexahydropyrrolo[2,3-*b*]indole) **1** with its rigid tricyclic molecular architecture still remains a challenge in organic synthesis.^3,5−7^ Inspired by nature, current synthesis strategies focus on a catalytic asymmetric dearomatization reaction of indoles to access pyrroloindoles.^3,8−10^

**Figure 1:**
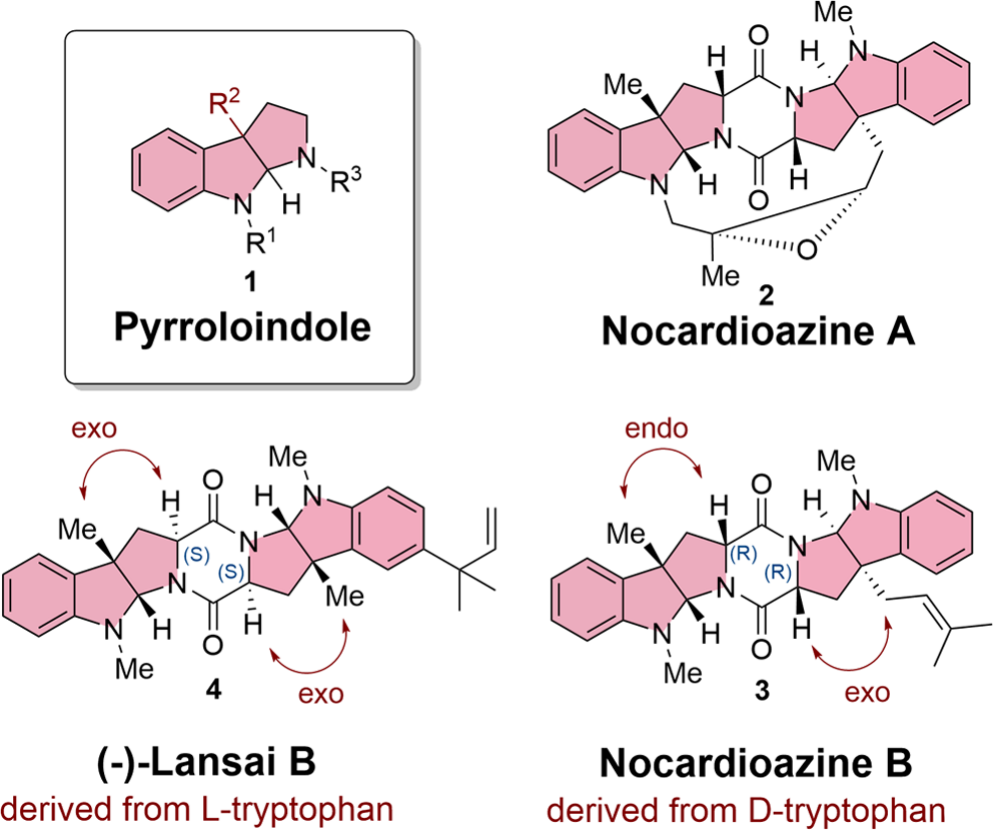
Lansai B (**4**) and nocardioazine A (**2**) and B (**3**) as DKP natural products containing a pyrroloindole structural motif (red).

Within the family of pyrroloindole natural products, diketopiperazines (DKP), with a pyrroloindole motif derived from a tryptophan, were studied regarding their biosynthesis (Figure 1).^11,12^ In the focus of these studies were nocardioazine A (**2**) and B (**3**), which were first isolated from a *Nocardiopsis* sp. (CMB-M0232) strain in an Australian marine sediment. Nocardioazine A has been shown to be a noncytotoxic inhibitor of the membrane protein efflux pump P-glycoprotein.^13^ In the past years, the absolute configuration of the stereogenic centers on the DKP core was discussed: Based on the finding of *cyclo*-*L**-*Trp-*L-*Trp DKP (*LL-*cWW) as a possible precursor of nocardioazine, extensive NMR spectral investigations, and biosynthetic speculation, the absolute configuration of the stereogenic centers at the DKP core was suggested to be an (*S*)-configuration.^13^ The first published approach for the total synthesis of nocardioazine B (**3**), starting with *L**-*configured tryptophans, revealed that the optical rotation of the synthetic final product was opposite in sign to the isolated natural product. In conclusion, the tryptophans in nocardioazine B (**3**) are d*-*configured, therefore meaning an absolute (*R*)-configuration of the stereogenic centers on the DKP core.^14^ Consequently, nocardioazine B (**3**) consists of an *endo*-pyrroloindole and an *exo*-pyrroloindole, which fits the macrocyclic structure of nocardioazine A (**2**).^15^ Further investigations on the biosynthetic pathway have shown that starting from *L**-*tryptophan as a natural amino acid, a cyclodipeptide synthase (CDPS) forms *LL-*cWW, which must be isomerized into its enantiomer *DD**-*cWW by an isomerase prior to prenylation and the final methylation.^11,16^

In comparison, lansai B (**4**), first isolated from *Streptomyces sp*. SUC1, an endophyte found on the aerial roots of *Ficus benjamina*, was proven to contain an *L**-*tryptophan-derived structure. The first total synthesis of lansai B having (*S*)-configured stereogenic centers on the DKP core revealed the same sign in optical rotation as the naturally isolated product.^12,15^ Therefore, lansai B (**4**) consists of two *exo*-pyrroloindole motifs with an absolute (*S*)-configuration in the DKP core (Figure 1).

Focusing on the synthesis of the pyrroloindole structural motif in these natural compounds, a methyltransferase is needed for this key step in the biosynthetic pathway.^11,16^ In nature, *S*-adenosyl methionine (SAM, **5**) is used as a methyl donor for SAM-dependent methyltransferase reactions. The methyl group of SAM (**5**) can be transferred by these methyltransferases to a large variety of acceptor molecules, such as small metabolites or even biopolymers, whereby *S*-adenosyl-homocysteine (SAH) (**6**) is formed as the byproduct.^17−22^ Methyltransferases can be further classified according to the atom on the substrate accepting the methyl group. Based on this classification, *C*-methyltransferases are comparatively rare (18%).^19^

**Scheme 1:**
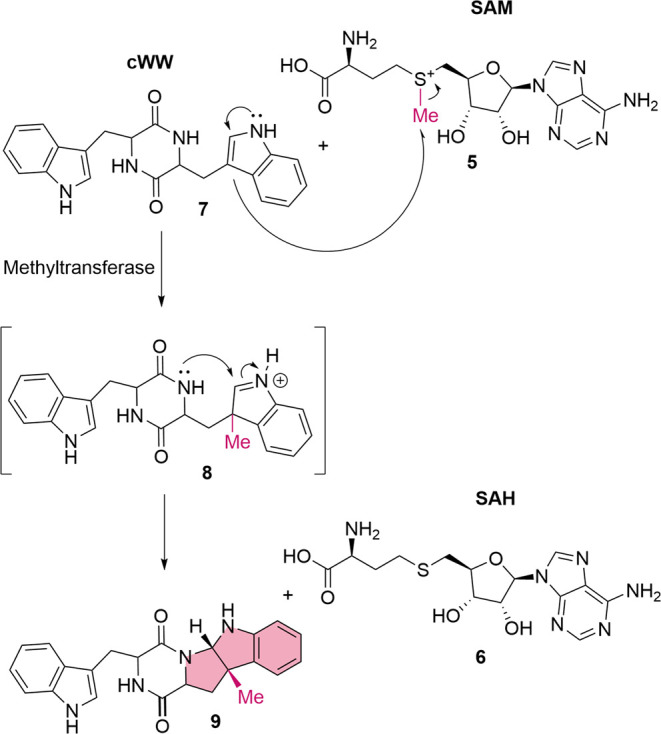
Mechanism for the Methylation of cWW with a Methyltransferase The methyl group from SAM (**5**) is transferred to the C3 position of the indole ring of the cWW 7, forming the pyrroloindole structural motif as the final step.

In the case of nocardioazine B (**3**) and lansai B (**4**), the methyl group from the cofactor SAM (**5**) is transferred by a suitable methyltransferase to the C3 position of the indole ring of cWW **7**, creating an electron sink on the indole nitrogen and forming a highly reactive iminium ion as an intermediate (**8**). As a next step, the nucleophilic nitrogen in the diketopiperazine ring attacks the C2 position of the former indole to form the target structure in the methylated product **9** (Scheme 1).^23^

The indole C3-methyltransferase from *Streptomyces* sp. HPH0547 is the first isolated enzyme known to accept *LL*-cWW **7** as a substrate. This enzyme catalyzes the indole C3-methylation and cyclization in diketopiperazines to form the methylated product.^23^ Li et al. describe a potential biosynthesis pathway in *Streptomyces* sp. HPH0547 assuming that an *L*-tryptophan-based isomer of the nocardioazine family is formed as the final product of the pathway, although natural nocardioazine B **3** is meanwhile known to contain *D*-tryptophans.^23^

Herein, we report on—potentially natural—substrates of the C3-methyltransferase StspM1, emphasizing its use for the possible synthesis of natural compounds, such as nocardioazine or lansai B, containing either *L**-* or *D**-*tryptophan-derived structures. Furthermore, we show that this methyltransferase reaction allows a diasteroselective and mild access to the methylated pyrroloindole framework in cyclodipeptides, which is not feasible with conventional organic chemistry methods so far. To showcase the synthetic use of this biocatalytic tool at a preparative scale, an enzymatic cascade coupling the main reaction with a cofactor recycling system was established after an optimization process with immobilized enzymes.

## Results and Discussion

### Biochemical Characterization of StspM1

**Scheme 2:**
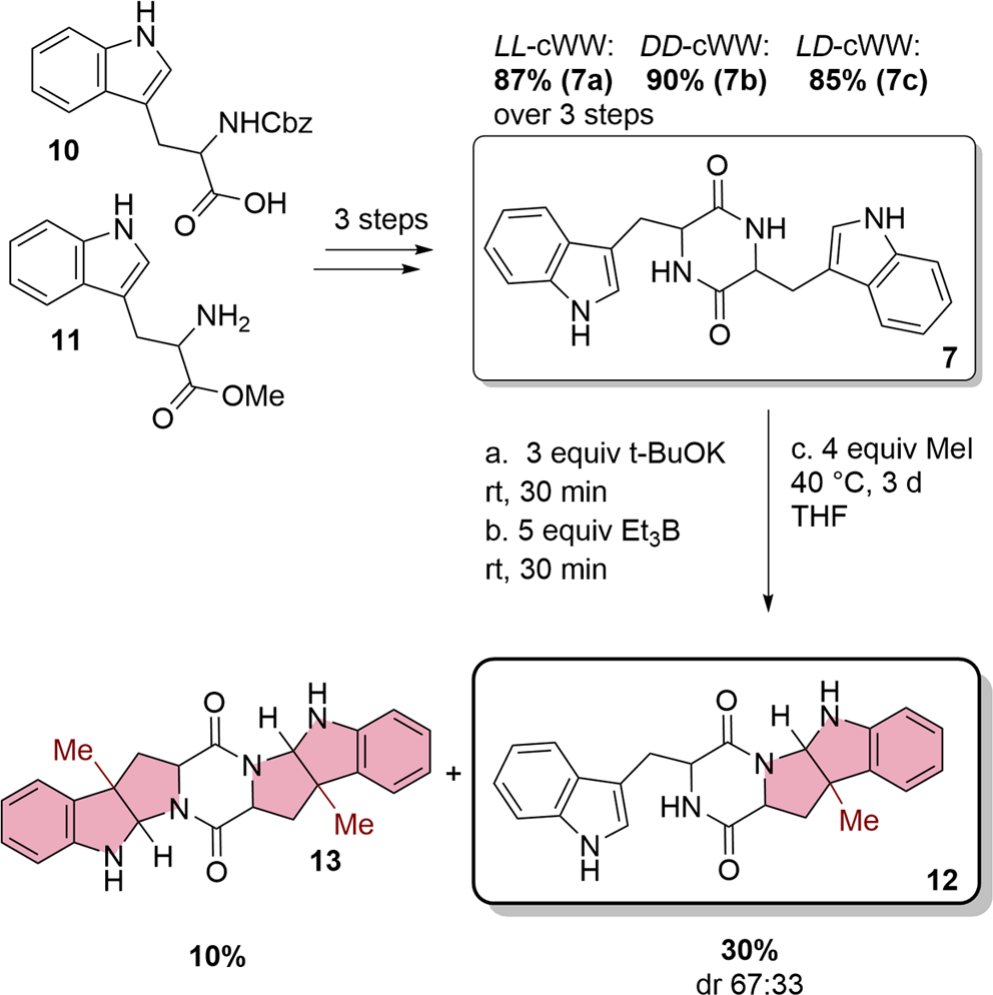
Three-Step Synthesis of the Single- and Double-Methylated cWW Substrates as References for the Identification of DKP Products Containing the Pyrroloindole Structural Motif (Light Red)

The methyltransferase StspM1 was heterologously expressed in *Escherichia coliBL21 Gold (DE3)* and purified using a nickel NTA column (Figure S1). For further investigations on the natural product of *Streptomyces* sp. HPH0547, different isomers of cWW **7** were synthesized with high yields of up to 90% in three steps, starting from protected tryptophans **10** and **11** (Scheme S1). For comparison, chemical methylation was carried out with methyl iodide as a methylation agent, resulting in low yields and diastereoselectivity of the corresponding pyrroloindoles (Scheme 2). In addition to the expected monomethylated product **12**, double methylation on both sides of DKP **13** was also observed. These chemically synthesized products served as references for the identification of the reaction products resulting from the biocatalytic methyltransferase reaction (Figures S2 and S3).

**Figure 2:**
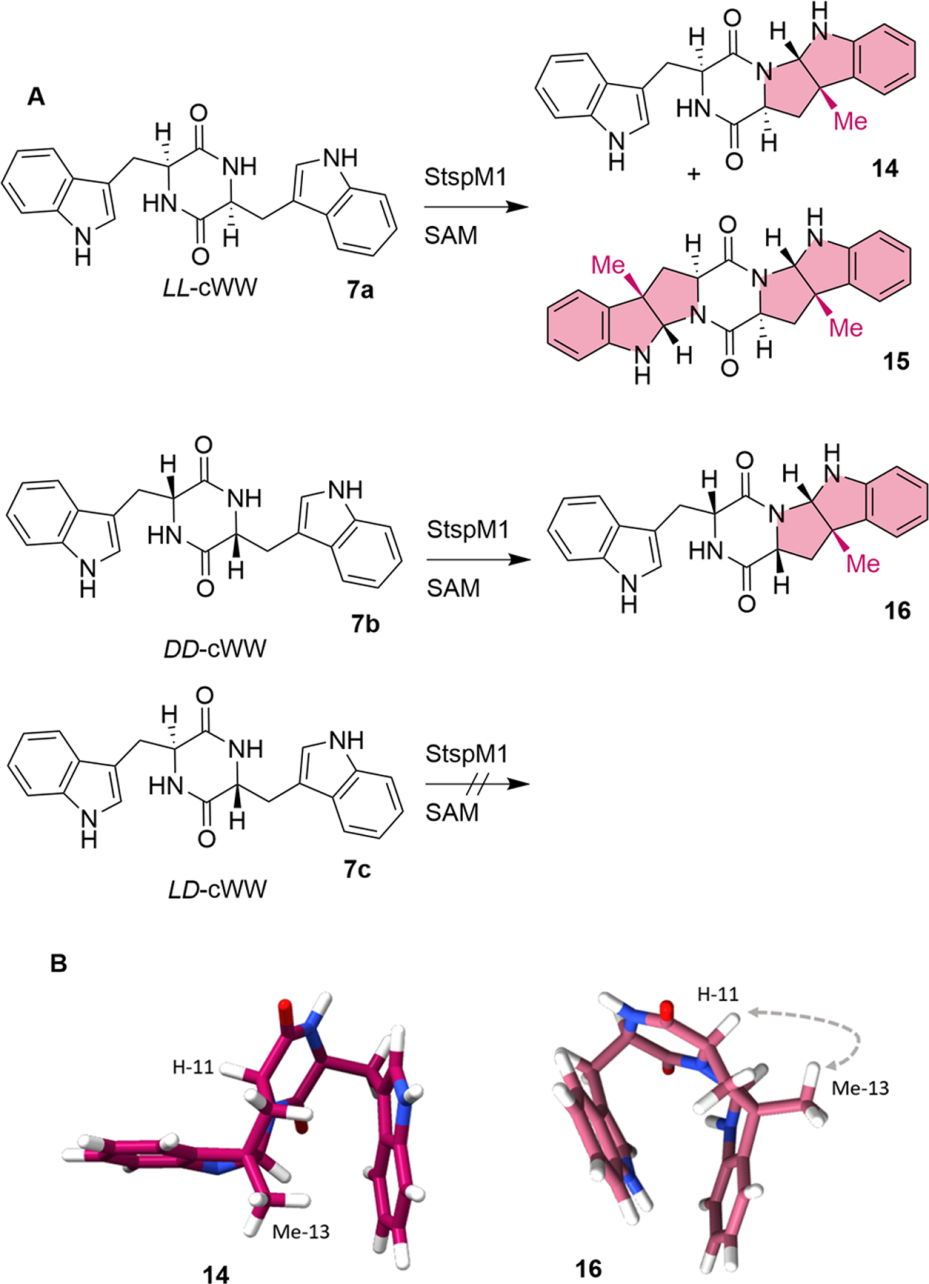
(A) Substrate acceptance (cWW isomers) of the methyltransferase StspM1. (B) DFT structures (r2SCAN-D4/def2-TZVP/C-PCM(H_2_O)) of single-methylated *LL-*cWW **14** (left) and *DD**-*cWW **16** (right). ROESY correlation was observed between H-11 and Me-13 in the single-methylated *DD**-*cWW only.

As Li et al. state *LL-*cWW **7a** as a substrate for StspM1, the enzymatic reaction with this compound was repeated under the same conditions as published (pH 7.5, 50 mM TRIS, 100 mM NaCl, 1 mM SAM, 1 mM cWW, 40 μM StspM1, 30 °C, 120 min).^23^ The methylation reaction was 10 times slower as reported and, even more surprisingly, the enzyme StspM1 did not just catalyze single methylation but also double methylation, which was not reported previously (Figure 2A and Table S3). To validate this result, an LC-MS analysis was performed and revealed masses of 387 *m*/*z* for monomethylated **14** and 401 *m*/*z* for double-methylated product **15**, showing a mass shift of +14 *m*/*z* for the additionally transferred methyl group. The retention times are in line with the chemically synthesized reference molecules (Table S3 and Figure S2), showing excellent diastereoselectivity (dr > 99:1).

For the biosynthesis of nocardioazines, *cyclo*-*D**-*Trp-*D**-*Trp DKP **7b** might serve as a precursor for the methyltransferase. When testing this substrate with the methyltransferase StspM1, only single-methylated product **17** was observed. *LD**-*cWW **7c** was not accepted as a substrate (Figure 2A and Table S3). For comparison, the recently investigated methyltransferase NozMT from *Nocardiopsis* sp. CMB-M0232 (sharing 57% sequence identity with StspM1), which is known to be involved in the biosynthesis of nocardioazine B, does not accept either of these substrates.^16^

To elucidate the absolute configuration of the newly formed stereogenic centers in methylated *LL-*cWW **14** and *DD**-*cWW **16**, ROESY NMR spectra were measured for identification of spatially adjacent protons. For single-methylated *DD**-*cWW **16**, a correlation between Me-13 and the H-11 was observed, proving a relative configuration in which the H-11 proton and the Me-13 methyl group face toward the same side of the molecule. In comparison, Me-13 and H-11 in single-methylated *LL**-*cWW **14** showed no correlation (Figures 2B, S45, and S46). The absolute configuration of the newly generated stereogenic center in both isomers is similar to the configurations in nocardioazine B **3** and lansai B **4**. Regarding selectivity, the methyltransferase StspM1 was found to be an excellent diastereoselective catalyst for the synthesis of the pyrroloindole structural motif found in both natural compounds.

**Figure 3:**
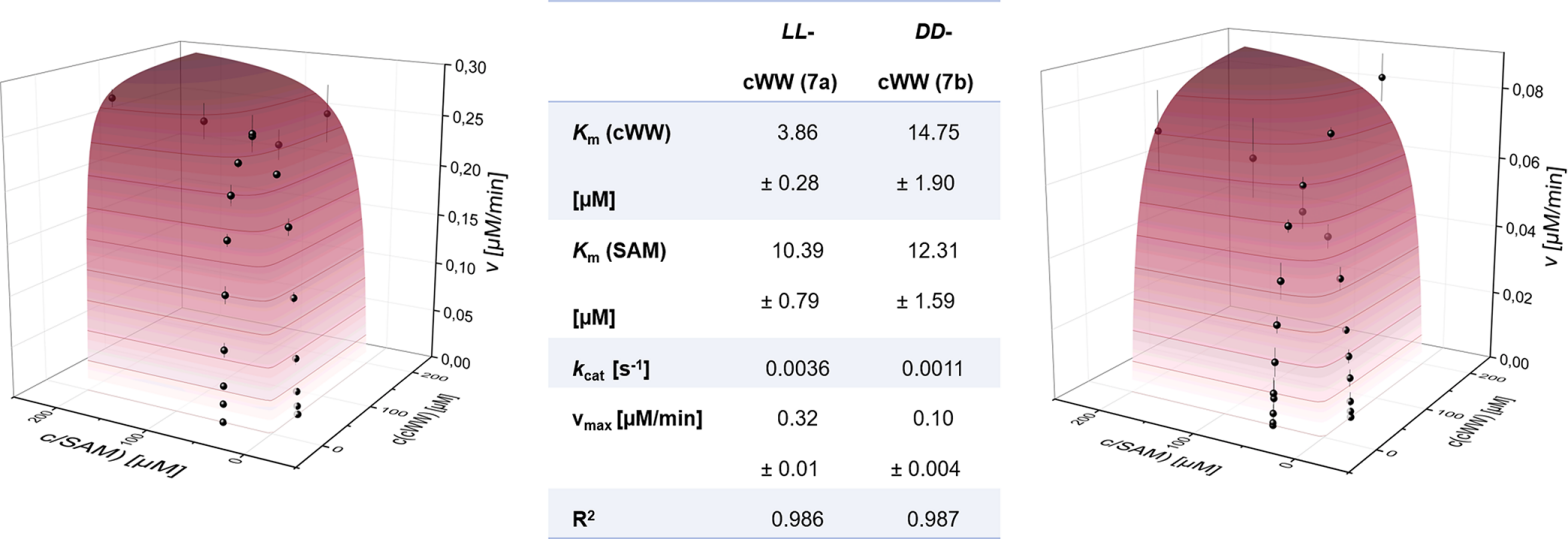
Michaelis–Menten kinetics and kinetic parameters of *LL-*cWW (left) and *DD-*cWW (right) for the biocatalyzed methylation using SAM as a cofactor.

The biocatalytic reactions were repeated under optimized in vitro assay conditions (KPi buffer 50 mM, pH 7.5, 2 mM SAM, 1 mM cWW, 100 μM StspM1, 40 °C),^23^ showing that the conversion rate of *LL-*cWW **7a** is two times higher than the conversion of *DD**-*cWW **7b**. Even under optimized conditions, *LD-*cWW **7c** was not converted (Table S3). For further investigations on this result and the comparison of both enantiomeric cWW substrates, Michaelis–Menten kinetics was performed to determine the kinetic parameters of StspM1 for these substrates (Figure 3). The concentration of either SAM or respective cWW was fixed at 50 μM, and the cWW substrate was used in a concentration range from 0.2 to 200 μM. The reactions were performed in a 96-well plate with an enzyme concentration of 1.5 μM. The methylation reaction was stopped after 5, 10, and 15 min by addition of 0.5% trifluoroacetic acid. To determine the substrate conversion rate, the commercially available bioluminescence-based Mtase-Glo Assay (Promega) was used, as previously reported.^24^ This assay, which detects the formation of SAH, was used to measure the consumption of SAM in the methyltransferase reaction.^25^

Plotting the reaction rate against the concentrations, *K*_m_ values of 3.86 μM for *LL-*cWW **7a** and 14.75 μM for its enantiomer *DD-*cWW **7c** were measured, indicating a higher affinity of the enzyme for *LL-*cWW. In addition, the *K*_m_ value for the second methylation step of *LL-*cWW **14** was determined as 32.45 μM, being 10 times higher than the *K*_m_ value of the monomethylation (Figure S7 and Table S4).

### Computational Analysis

**Figure 4:**
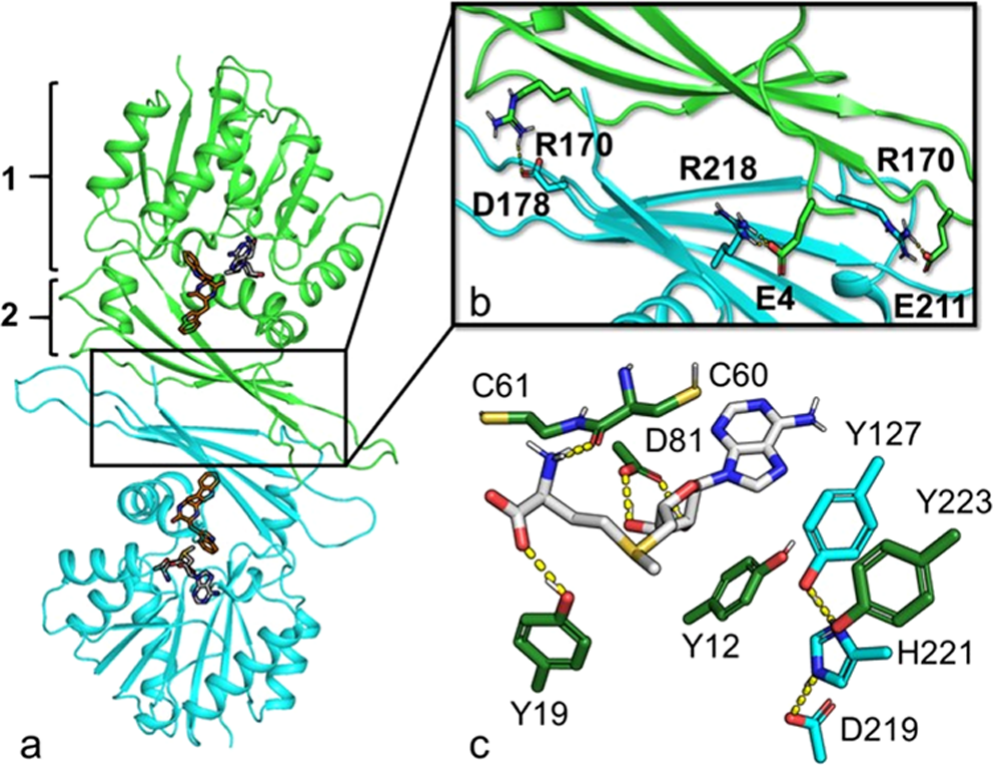
(a) Dimeric model of StspM1 in complex with *LL*-cWW **7a** and SAM (**5**). (b) Predicted dimerization interface: 1 and 2 highlight the parts of the 3D structure corresponding to the Rossmann fold and the β-cap domain, respectively. (c) Docking pose of the SAM cofactor (gray sticks) and binding site (green sticks) including the Y127-H221-D219 proton shuttle (cyan sticks) in the minimized model of StspM1. Polar interactions: yellow dashed lines.

To understand the relationship between substrate stereoselectivity and reactivity at the molecular level, a 3D model of the protein in complex with each tested cWW was generated for use in subsequent molecular dynamics (MD) simulations. The protein 3D structure was modeled using Colabfold^26,27^ and consisted of a typical Rossmann-type α/β fold branched to a β-cap domain (Figure 4a). The model is of high quality, with a pLDDT score per residue above 70 for 95% of all residues. The substrate binding modes were predicted by flexible docking with Glide.^28,29^ To determine the oligomerization state of StspM1, size exclusion chromatography was performed. The deviation of the measured weight (70.5 kDa; Table S5 and Figure S8) from the theoretical size of a dimer (61.8 kDa) could be explained by the nonperfectly globular shape of the dimer and has also been observed for the homologous PsmD methyltransferase, which is proven to be a dimer via the crystal structure.^24^ Furthermore, a dimeric structure was also predicted by GalaxyHomomer.^30^ The presence of solvent-exposed apolar amino acids in the β-cap domain suggests a plausible dimerization mediated by this domain. Although four interfacial salt bridges were predicted (Figure 4b), it is unclear whether they could have any role in stabilizing the interface given the low conservation of the residues involved (Figure S9) and the absence of coevolutionary coupling between them.

**Figure 5:**
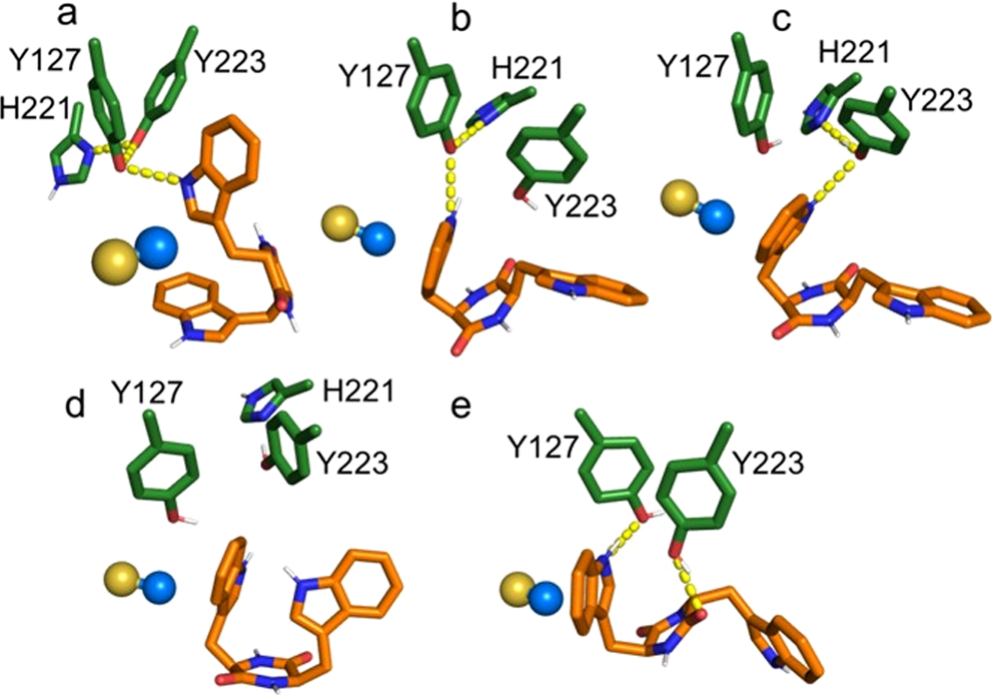
Binding modes of the reactive conformations of unmethylated *LL*-cWW **7a** (a) and unmethylated *DD*-cWW **7b** (b–d). (e) Unreactive conformation of unmethylated *LD*-cWW **7c** fulfilling both the C3_SAM_–C7_indole_ distance and C3_SAM_–C7_indole_–N9_indole_ angle constraints. Selected binding pocket residues (green), SAM (**5**) sulfur and methyl group (golden and blue spheres), and cWW substrates (orange) are shown. Polar interactions: yellow dashed lines.

As reported in other methyltransferases,^31,32^ docking predictions show that the SAM (**5**) cofactor forms hydrogen bonds with highly conserved residues, including D81 (Figure 4c) and both cysteines of the conserved CCGTG motif (Figure S9). By analogy with PsmD,^24^ the cofactor is surrounded by four tyrosine residues (Y12, Y19, Y127, and Y223), three of which (Y12, Y19, and Y127) are highly conserved (Figure S9). We show that Y127 is a catalytic residue, since its substitution by alanine or phenylalanine abolished the enzyme activity (Figure S26). By analogy with the PsmD methyltransferase,^24^ we indicate that Y127 may be involved in stabilizing the bound substrate by interacting with the amine moiety of the reactive indole ring (Figure 5). Despite the low conservation of Y223, its proximity to Y127 makes this residue potentially involved in catalysis. Moreover, coevolution-based contact predictions reveal that both are evolutionarily coupled.^33^ Finally, we also indicate that Y223 makes direct hydrogen bond interactions with the substrates, including the amine moiety of the reactive indole in the case of unmethylated *DD*-cWW **7b** (Figure 5).

Molecular modeling reveals that the configuration of cWW substrates influences their conformational preference in the binding pocket (Figure 5). A per-residue decomposition of the binding effective energy computed with MM-GBSA^34^ shows that they are stabilized by aromatic π-stacking and hydrogen bond interactions with neighboring residues (Figures S10 and S12). As specified in the Methods section (see Supporting Information), we used five geometrical criteria to identify cWW conformations compatible with an S_N_2 methyl transfer mechanism in our MD simulations (Figure S12). In the remainder of the manuscript, we refer to these binding poses as “reactive conformations.” To optimize their interactions with the binding site, we subsequently refined their geometry at the semiempirical QM level. Given the role of Y127 and its proximity to Y223, we supposed that polar interactions involving the substrate’s reactive indole and these residues are essential to catalysis. Thus, we compared the occurrence of such interactions among the cWW to rationalize the difference in reactivity observed in experiments.

The unmethylated *LD*-cWW **7c** does not form any reactive conformation. This is in agreement with experimental data showing that this substrate is inactive. Only a single conformation (Figure 5e) fulfilling two of the five geometric criteria (distance C3_SAM_–C7_indole_, angle C3_SAM_–C7_indole_–N9_indole_) could be identified. Of all tested substrates, unmethylated *LL*-cWW **7a** shows the largest conformational mobility in the binding pocket (Figure S13). However, only one reactive conformation (Figure 5a) could be isolated twice in the MD trajectory. The reactive indole in this conformation forms a hydrogen bond with the side chain of Y127. Unmethylated *DD*-cWW **7b** adopts two types of reactive conformations isolated in 19 frames of the MD trajectory, which contains ∼137 000 frames. While 4 conformers out of 19 display an envelope-like geometry (Figure 5b,c), the rest adopt a boat-like conformation (Figure 5d) due to the high proximity of the two indole moieties fostered by the *DD* configuration (Figure S14). However, hydrogen bonds of the reactive indole to the Y127 and Y223 side chains were only found in poses showing an envelope-like conformation, whereas boat-like conformations do not interact with any of these catalytic residues and are, thus, unlikely to be catalytically active.

**Figure 6:**
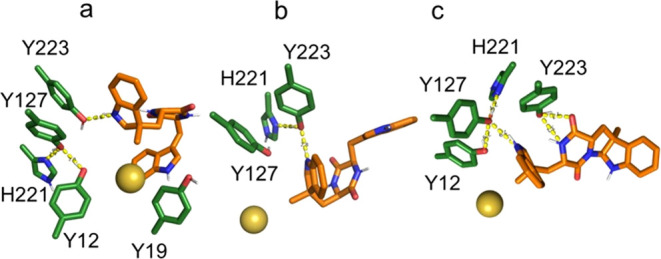
Binding modes of the reactive conformations of the reaction intermediates formed after methyl transfer. Single-methylated *LL*-cWW **14** (a) and *DD*-cWW **16** (b) before pyrroloindole cyclization, and double-methylated *LL*-cWW **15** (c) before pyrroloindole cyclization. The golden sphere highlights the position of the demethylated sulfur group of the SAM cofactor. The legend is identical to Figure 5.

Both unmethylated substrates present a comparably low number of polar interactions with Y127. Therefore, this parameter cannot be used to explain the difference in reactivity between these two substrates. Alternatively, the lower reactivity of the *DD* enantiomer might be rationalized by the detrimental influence of its configuration on the energy of the transition state due to the high likelihood of forming boat-like conformations. For both enantiomers, QM calculations show that the loss of planarity of the reactive indole upon methyl transfer favors the formation of a hydrogen bond with Y223 in the reaction intermediate (Figure 6a,b). We suggest that Y223 could assist in the cyclization of the pyrroloindole ring prior to the formation of the single-methylated cWW substrates.

**Figure 7:**
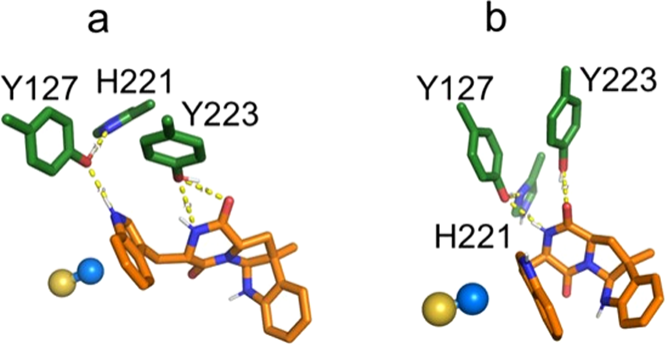
Binding modes of the reactive conformations of the single-methylated *LL*-cWW **14** (a) and *DD*-cWW **16** (b) substrates for the second methylation reaction. The legend is identical to Figure 5.

Experiments show that single-methylated *LL*-cWW product **14** can react as a secondary substrate and accept a methyl group on its additional reactive indole. This substrate forms reactive conformations in 12 frames of the MD trajectory. Although Y223 preferentially interacts with the DKP motif in most of the MD trajectory, a hydrogen bond between the reactive indole and the hydroxyl group of this residue can be observed in 5 frames. In the QM-optimized geometries, the reactive indole becomes hydrogen-bonded to the Y127 side chain in 3 out of these 5 frames (Figure 7a). Upon methyl transfer, the loss of planarity of the reactive indole doubles the occurrence of this interaction (Figure 6c).

Conversely, experiments show that single-methylated *DD*-cWW substrate **16** is inactive. Despite forming reactive conformations in 18 frames of the MD trajectory, the reactive indole in these conformations does not form any hydrogen bond with Y127 or Y223. The absence of a hydrogen bond between the reactive indole of this compound and these important tyrosines could explain its inactivity as a substrate. In addition, the reactive conformations of this substrate exclusively adopt boat-like geometries (Figure 7b), which, by analogy with unmethylated *DD*-cWW substrate **7b**, would likely increase the energy of the transition state.

### Preparative Enzymatic Methylation

**Scheme 3:**
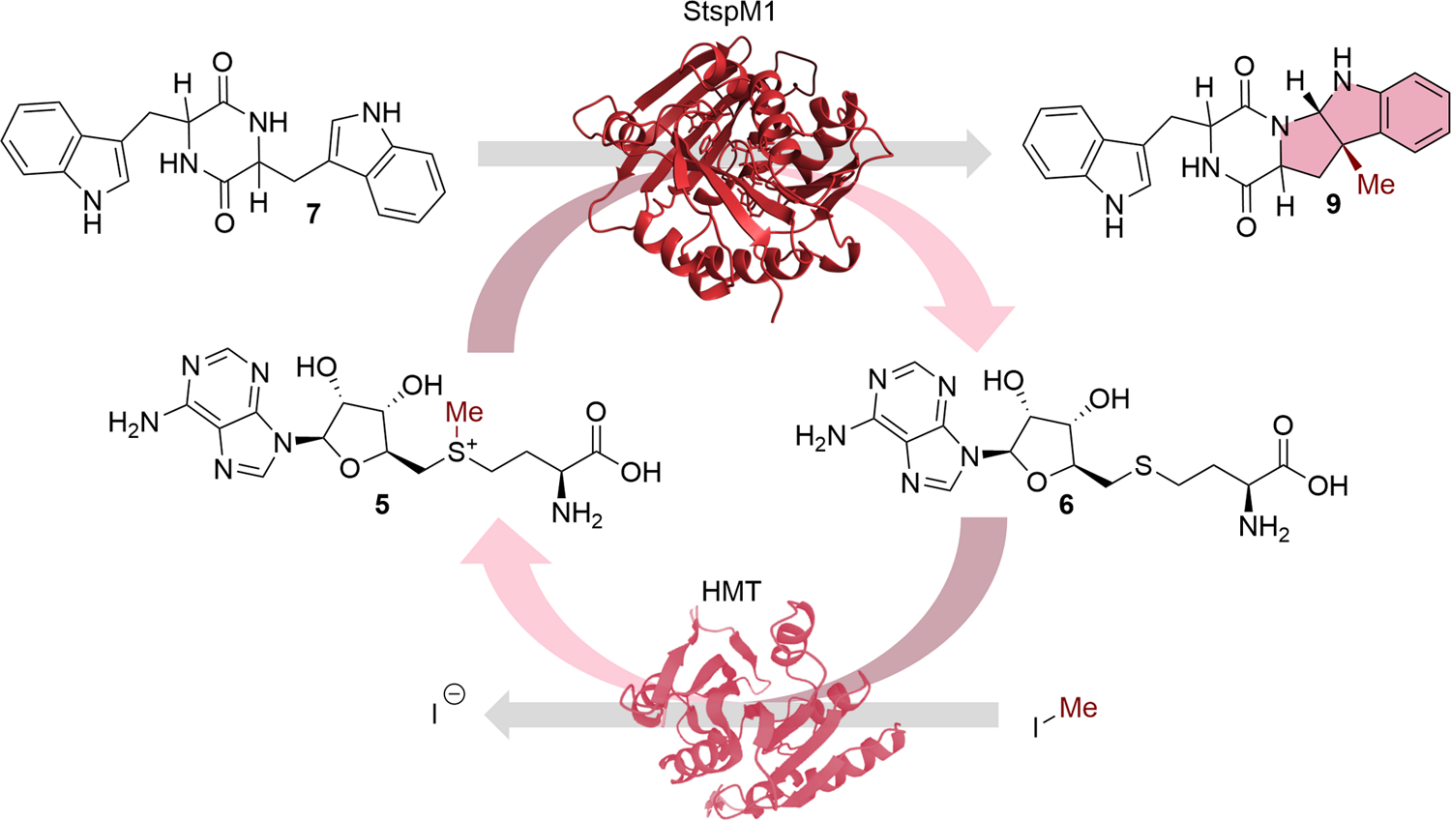
SAM (**5**) Recycling System Described by Seebeck et al.^35^ The HMT regenerates SAM (**5**) by transferring a methyl group from methyl iodide to SAH (**6**), which is produced in the StspM1-catalyzed reaction.

For the synthetic utility of the methyltransferase reaction, the stoichiometric demand for the high-priced cofactor SAM (**5**) remained a problem, which can be tagged by using a SAM recycling system based on the halide methyltransferase from *Chloracidobacterium thermophilum* (*Ct*HMT) described by Seebeck et al.^35^ The HMT transfers a methyl group from methyl iodide to SAH (**6**), forming SAM (**5**), which is transformed back to SAH (**6**) in the methyltransferase reaction catalyzed by StspM1 (Scheme 3).

**Figure 8:**
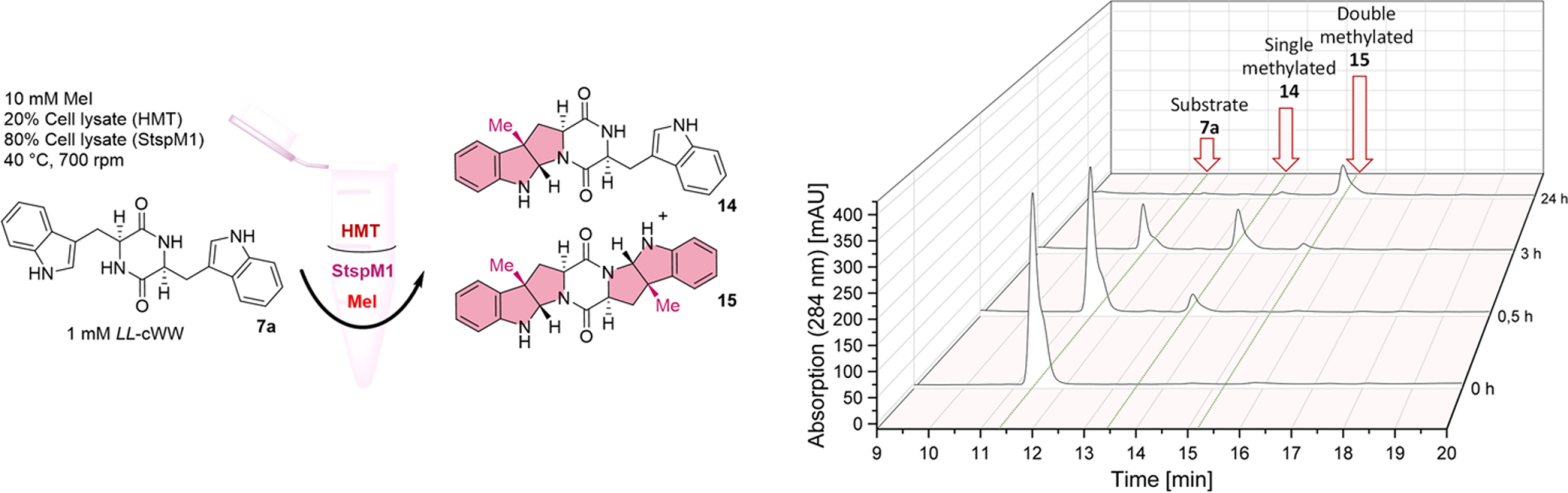
(Left) Biocatalytic methylation of *LL-*cWW **7a** with StspM1 using a SAM recycling system. (Right) Chromatograms of the time-dependent (0, 0.5, 3, 24 h) conversion of *LL-*cWW **7a** to its single-methylated **14** and double-methylated **15** products by measuring the absorption at 284 nm and retention times with HPLC.

To increase the synthetic utility of the methyltransferase, the use of lysates rather than purified enzymes is often beneficial for several reasons. First, using lysates does not require time-consuming and cost-intensive protein purification. Second, no addition of expensive SAH or SAM cofactors is needed, given the sufficient amount of SAM already available from the lysed cells. As the HMT lysate shows higher activity and faster conversion rates than the StspM1 lysate (Figures S15 and S16), an excess of StspM1 lysate was used for a prior test with the *LL-*cWW substrate. Despite both enzymes being more active at higher temperatures, a reaction temperature of 40 °C should not be exceeded, since the boiling point of methyl iodide is at 42 °C. Due to the eventual evaporation of this compound at this temperature, an excess of 10 mM methyl iodide was used for the reaction. The relative conversions were monitored via HPLC by measuring the absorption at 284 nm after 0.5, 3, and 24 h of incubation (Figure 8).

After 24 h, almost full conversion to the double-methylated product was observed (Figures S17 and S18). The negative controls with changing either the StspM1 lysate or the HMT lysate to a lysate with an empty vector showed no conversion. The lower peak area of the methylated products compared to the substrate can be explained due to the lower extinction coefficient. When forming the pyrroloindole motif from the indole motif, the aromatic system decreases, leading to a lower absorption at 284 nm. The extinction coefficient of substrate **7a** is 8607 M^–1^ cm^–1^, while single-methylated product **14** (4460 M^–1^ cm^–1^) as well as double-methylated product **15** (2270 M^–1^ cm^–1^) showed lower values (Figure S4).

**Figure 9:**
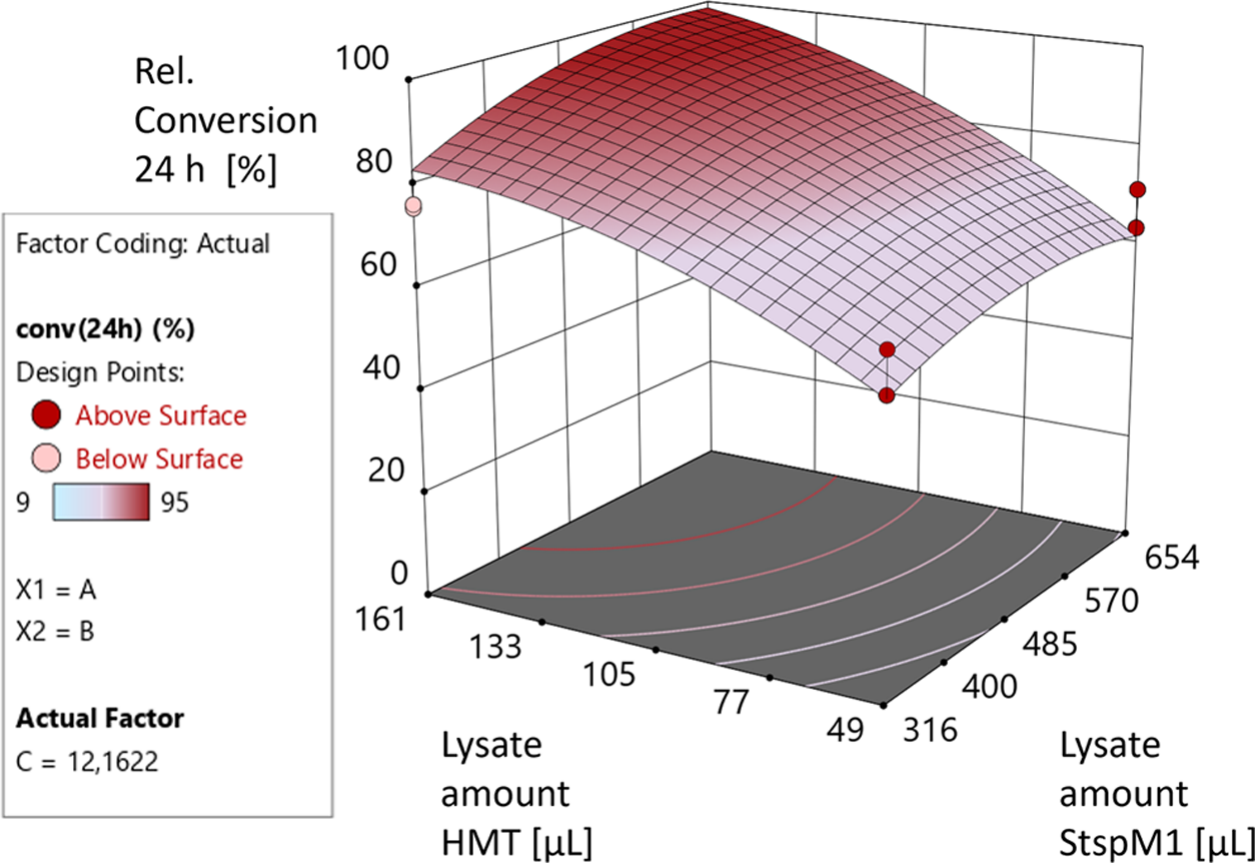
Result of the design of experiments approach. The conversion after 24 h of *LL-*cWW **7a** is modeled by the hypersurface in the 3D diagram.

To optimize the efficiency of the system for synthetic usage at a preparative scale and to minimize the amount of catalysts needed, a response surface design of experiments was performed. In this approach, different experimental conditions were tested by varying the methyl iodide concentration, the StspM1 lysate amount, and the CtHMT lysate amount as independent variables. For each experimental condition, the conversion rate after 24 h was finally measured via HPLC. For the design of experiments approach, 34 single experiments were performed in total (Table S6). The conversion after 24 h of *LL-*cWW **7a** was modeled using a hypersurface model in the 3D diagram (Figures 9 and S19).

The design of experiments approach shows that the conversion rate is mainly dependent on the added amount of the CtHMT lysate in the reaction medium. To obtain a maximal conversion after 24 h with a minimum amount of the StspM1 lysate, we determined that a v/v ratio of 34% for the StspM1 lysate, 16% for the HMT lysate, and 12 equiv of methyl iodide can be used. To confirm these findings, an additional verification experiment was carried out using these exact conditions. As a result, the expected substrate conversion (over 90%) was obtained (Table S8).

With these optimized conditions, a preparative scale experiment with 50 mg of the substrate (0.13 mmol; 134 mL final volume) was carried out (StspM1 catalyst load: 3.58 μM or 0.37 mol %). After 24 h, a conversion of 91% was calculated, fitting the results of the analytical scale reactions. The resulting mixture contained a product ratio of 46% of monomethylated and 44% of dimethylated products. After workup, a product yield of 36% could be achieved (19% monomethylated and 17% dimethylated products). For the workup, the residual methyl iodide was quenched with a sodium hydroxide solution, and the proteins in the lysate were precipitated with ammonium sulfate. When adding the extraction solvent (ethyl acetate), residual proteins aggregated, forming an inseparable interphase. Due to problems with the extraction, up to 60% of the products were lost.

**Figure 10:**
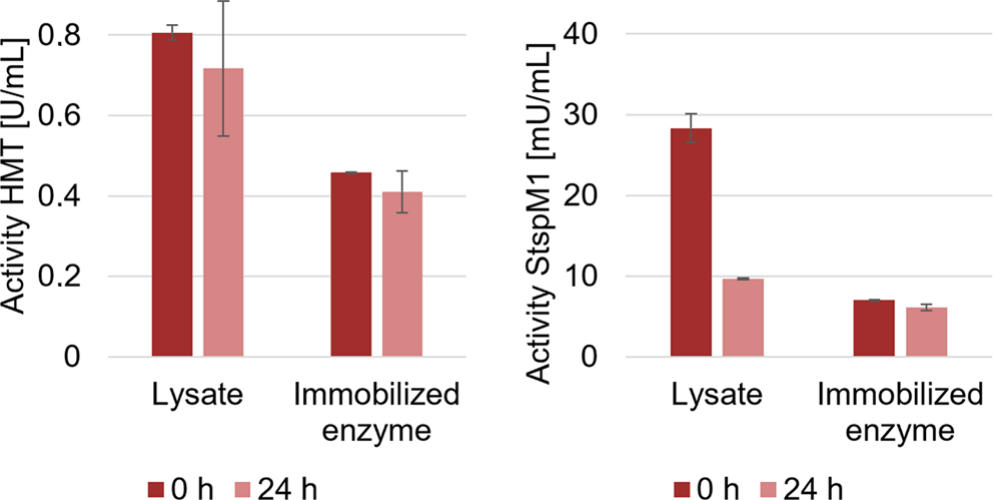
Activity of HMT (left) and StspM1 (right) in the lysate and the immobilized form before (0 h) and after (24 h) incubation at 40 °C.

To solve this problem, the enzymes (StspM1 and HMT) were immobilized on Ni-NTA agarose beads. By comparing the enzymatic activity of the lysates with the immobilized enzymes, we show that the immobilized StspM1 methyltransferase is four times less active than the enzyme in the lysate and that the immobilized HMT is two times less active than in the corresponding lysate (Figures S20 and S21). As immobilization can increase the stability of enzymes, the lysate activity and the activity of the immobilized enzymes were measured again after an incubation of 24 h under the same reaction conditions employed in the first preparative scale experiment. The activity of the HMT in the lysate and in the immobilized form decreased to the same extent after the incubation time (lysate: 11%, immobilized enzyme: 10%). In comparison to the immobilized enzyme StspM1 having only a small decrease in activity over time (13%), a decrease of 66% of the initial StspM1 methyltransferase activity in the lysate was detected after 24 h (Figure 10). These results show that the immobilization has a stabilizing effect for StspM1.

As immobilized enzymes were to be used to solve the problematic reaction workup, the SAH/SAM initially provided by the lysates had to be added separately to the reaction. To determine the sufficient amount of SAH (**6**), different concentrations were tested. The same conditions as in the design of experiments approach were applied, and the conversion was measured after 24 h. For 0.1 and 0.05 equiv of SAH (**6**), the conversion was close to the expected 90%. Adding less SAH (**6**) leads to lower conversions (Figure S22).

**Figure 11:**
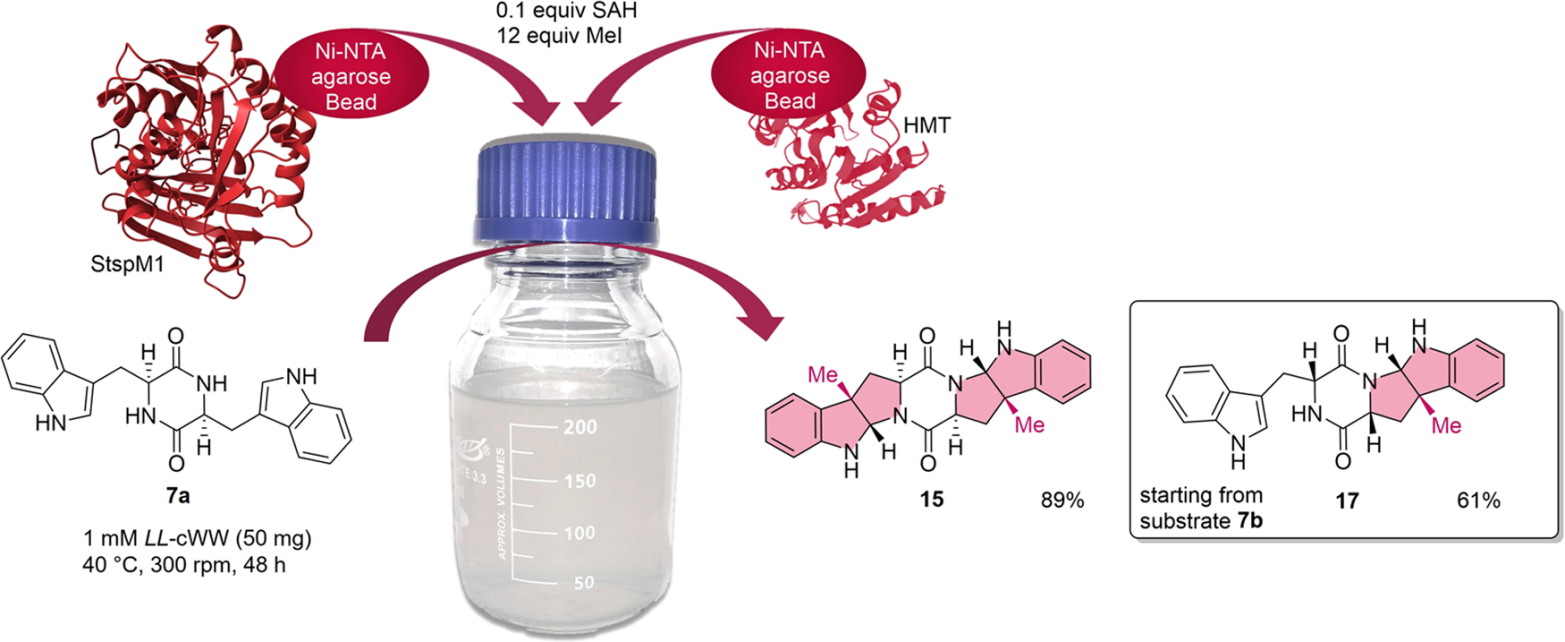
Setup of the preparative enzymatic methylation of *LL-*cWW **7a** as a substrate, leading to a yield of 89% of double-methylated product **15**.

The preparative experiment at a 50 mg scale was repeated under the same conditions with an additional amount of SAH (0.1 equiv). After 24 h of incubation, a conversion rate of 97% was obtained with 20% and 78% of monomethylated **14** and double-methylated **15** products, respectively. The reaction was quenched by the addition of ammonium thiosulfate before filtering off the Ni-NTA beads and extracting the reaction products with ethyl acetate. A yield of 89% was obtained, with a final mixture containing 20 and 69% of monomethylated **14** and double-methylated **15** products, respectively. In comparison with the reaction with lysates, the workup required less solvent and the isolated yield was increased up to 53%. As the stability of the StspM1 enzyme is increased due to the immobilization, the incubation was prolonged to 48 h to shift the substrate conversion further toward double-methylated product **15** while monitoring the conversion rate at different time points (Figure S23). After 48 h, the reaction was stopped due to no further substrate conversion, leading to a final conversion of 91% of cWW substrate **7a** to double-methylated product **15**. A final yield of 89% was achieved (Figure 11). To prove the applicability of this method, *DD*-cWW **7b** was also used as a substrate. The reaction was performed with double the amount of both enzymes used for the conversion of *LL*-cWW **7a** to compensate for the lower conversion rates of *DD*-cWW substrate **7b**, as shown previously. The reaction was stopped after 3 days, leading to a conversion rate of 63% and a final yield of 61% of single-methylated product **16** (Figure S24).

## Conclusions

In the present study, the methyltransferase StspM1 was used for the synthesis of the pyrroloindole structural motif in diketopiperazines. In comparison to conventional organic synthesis, the reaction is carried out at pH 7.5 in an aqueous system with excellent conversion rates and diastereoselectivity. Different cWW stereoisomers were tested as substrates, leading to single or double methylation depending on the configuration of the DKP structural motif. Computational simulations at the classical and QM levels were conducted to rationalize the difference in substrate stereoselectivity observed in experiments. For the synthetic utility of the methyltransferase, a cofactor recycling of SAM was successfully implemented by using a halide methyltransferase. The reaction was optimized via a design of experiments approach, and the use of both lysates and immobilized enzymes was compared at a preparative scale. With this new protocol, which incorporates the SAM recycling system and enzyme immobilization, it is now possible to perform one-pot enzymatic methylation of cWW substrates on a preparative scale with high yields. The reported setup allows for efficient reaction workup and increases catalyst stability.

## Supporting Information Available

Additional experimental details; materials; and methods, including ^1^H NMR data and HPLC chromatograms (PDF)
List of enzymes used for alignment (TXT)

## Abbreviations

SAM: *S*-adenosyl-L-methionine
SAH: *S*-adenosyl-L-homocysteine
cWW: cyclic tryptophan-tryptophan dipeptide
DKP: diketopiperazine
HMT: halide methyltransferase
SEC: size exclusion chromatography
