# Supplementary material for: Application of the C3-Methyltransferase StspM1 for the Synthesis of the Natural Pyrroloindole Motif
Source: ACS Catal. 2023 Dec 14;14(1):227–36. doi: 10.1021/acscatal.3c04952 (PMC10775177; doi:10.1021/acscatal.3c04952)
Supplement: Supplementary file 1 — cs3c04952_si_001.pdf [file cs3c04952_si_001.pdf]

Application of the C3-methyltransferase StspM1 for the synthesis of the natural pyrroloindole motive

Mona Haase,[a] Benoit David,[b] Beatrix Paschold,[a] Thomas Classen,[c] Pascal Schneider,[a] Nadiia Pozhydaieva,[a] Holger Gohlke,[b,d] Jörg Pietruszka[a,c]*

[a] Institute for Bioorganic Chemistry & Bioeconomy Science Center (BioSC), Heinrich Heine University Düsseldorf in Forschungszentrum Jülich, 52426 Jülich, Germany

E-mail: j.pietruszka@fz-juelich.de

[b] Institute of Bio- and Geosciences (IBG-4: Bioinformatics) Forschungszentrum Jülich, 52426 Jülich, Germany

[c] Institute of Bio- and Geosciences (IBG-1: Bioorganic Chemistry) & Bioeconomy Science Center (BioSC), Forschungszentrum Jülich, 52426 Jülich, Germany

[d] Institute for Pharmaceutical and Medicinal Chemistry & Bioeconomy Science Center (BioSC), Heinrich Heine University Düsseldorf, 40225 Düsseldorf, Germany

Table of Contents

Computational methods S4

Protein structure modeling S4

Docking calculations S4

Ligand parametrization S4

Molecular dynamics simulations S5

Geometric analyses S6

Geometric rules for defining cWW reactive conformations S6

QM calculations S7

Binding free energy decomposition S7

Residue conservation analysis S8

Biological methods S9

Protein sequences S9

Vectors, bacterial strains, and media S9

Transformation S10

Gene expression S10

Enzyme purification S11

Size exclusion chromatography S11

General procedure for Mtase glo assay S12

In vitro assay S12

HMT activity S13

Design of experiment S14

Enzymatic preparative scale reaction S14

Immobilization protocol S15

Enzymatic preparative scale reaction with immobilized enzyme S15

Mutagenesis S16

Chemical methods S18

General information S18

Extinction coefficient S19

*Synthesis of Methyl ((Benzyloxy)Carbonyl)-Tryptophyl-Tryptophanate (**S3**)*[44] S19

Synthesis of Cyclo-tryptophan-tryptophan diketopiperazine 7 (cWW)[44, 45] S23

Synthesis of methylated cWW references[46] S26

Figure S1: Protein purification S30

Figure S2: Retention times of substrates and products + calibration + extinction coefficient S31
Figures S5+6: Glo Assay calibration S33
Figures S7: Michaelis Menten Kinetics S34
Figure S8: SEC of StspM1 ..... S34
Figures S9-14: Computational analysis S35
Figures S15-18: Lysate activities S41
Figure and equation 19: Design of experiment S43
Figures S20+21: Activity of immobilized enzymes S44
Figure S22: SAH equivalents S45
Figures S23+24: Preparative enzymatic methylation S45
Figure S25: SDS gel of immobilization process S47
Table S3: Conversion under Initial conditions S49
Table S5: SEC of StspM1 ..... S50
Table S6-8: DOE conditions and validation S51
NMR spectra S54

Computational methods

Protein structure modeling

The monomeric structure of StspM1 was modeled using AlphaFold2[1, 2]. The dimeric assembly was predicted using Galaxy Homomer[3], and the interface geometry was subsequently optimized using Galaxy Refine[4]. The SAM cofactor was docked in its binding pocket using the coordinates of the SAH cofactor from the PsmD crystal structure (PDB ID 7ZKH)[5].

Docking calculations

The binding poses of both unmethylated (*LL*, *DD*, and *LD*) cWW were predicted with the Glide SP[6, 7] module of the Schrödinger software suite (2021-4). The docking predictions were performed using the OPLS_2005 force field[8]. The protein structure was prepared using the prepwizard[9] module. The substrate conformers were sampled using the Schrödinger LigPrep[1] module at pH 7.4. Based on the current knowledge of the methyltransferase mechanism,[10] a harmonic distance restraint of 10 kcal mol^{-1} $Å^{-2}$ was enforced to ensure that the substrate carbon atom involved in the methyl transfer stays within a radius of 3 Å to the SAM methyl group. The cWW were docked in the binding pocket of each subunit of the dimeric structure.

Ligand parametrization

The RESP fitting scheme[11] was used to derive the partial charges of all ligands from their electrostatic potential using Multiwfn 3.8[12]. The electrostatic potential was computed on the HF/6-31G* level of theory using the ORCA 5.0.3 simulation software[13]. The non-bonded parameters of all ligands were derived from the gaff2 force field[14] using the parmchk2 module of the Antechamber program from AmberTools21.[15]

Molecular dynamics simulations

The models generated from docking were subjected to seven replicas of MD simulations of 200 ns simulation time each. The ff14SB[16] and gaff2 force fields were used to model the protein and ligands, respectively. The protein was solvated in a truncated octahedral box filled with TIP3P water molecules[17]. 12 sodium ions were added to the solvent to neutralize the net charge of the protein. PROPKA 3.0[18] was employed to assign the protonation states of histidine and acidic residues according to a pH of 7.4. Internal water molecules were modeled using Metatwist[19-21] based on 3D-RISM calculations[22, 23]. Long-range electrostatic interactions beyond a 9 Å inter-atomic distance cutoff were treated using the Particle Mesh Ewald algorithm[24]. Minimization was performed using the pmemd.MPI module of the AMBER21 software[25], while pmemd.cuda[26] was used for all subsequent steps.

Prior to MD simulations, each model was minimized in three stages. 1000 steps of steepest-descent were first applied to optimize the distribution of solvent molecules while keeping the protein restrained. In the second stage, the whole system was minimized using 10000 steps of steepest descent followed by 40000 steps of conjugate gradient minimization. To ensure a near-attack positioning of the substrate, harmonic distance restraints of 10 kcal mol^{-1} $Å^{-2}$ were applied to keep the substrate carbon atom involved in the methyl transfer stays close to the SAM methyl group. During this stage, the binding mode of the SAM cofactor was optimized by enforcing three additional harmonic distance restraints with two neighboring protein residues. Two restraints were applied between the hydroxyl groups of the ribose moiety and the carboxylate oxygen atoms of the D81 residue to ensure the correct positioning of the SAM ribose moiety.

Langevin dynamics in the NVT ensemble[27] was performed for 500 ps to thermalize the system at 300K. Density equilibration in the NPT ensemble was conducted for 5 ns at 1 bar using

positional restraints of 10 kcal mol^{-1} $Å^{-2}$ on all protein and ligands atoms. The SHAKE algorithm[28] was employed to constrain the vibrational motions of C-H bonds, allowing the use of a 2 fs time step. MD simulation snapshots were saved every 10 ps.

Geometric analyses

The CPPTRAJ program[29] of AmberTools21 was used for all geometric (distances, angles) analyses, excluding the first 10 ns of each simulation trajectory. We excluded from analyses all simulation snapshots in which the cWW substrate methyl acceptor (C7 atom) is beyond a threshold distance of 5 Å from the SAM cofactor methyl group (C3 atom). The geometric criteria used to determine hydrogen-bonding were a donor (D) – acceptor (A) distance of 3.5 Å and a D-H...A angle greater than 135°.

Geometric rules for defining cWW reactive conformations

Five geometry criteria were used to isolate reactive conformations from MD trajectories. First, in agreement with published mechanistic data [30, 31], we propose that the distance between the cofactor reactive methyl moiety ($C3_{SAM}$) and the nucleophilic carbon ($C7_{indole}$) of the cWW reactive indole ring should be less than 3.2 Å. Second, we suggest that the angle formed by the methyl donor and acceptor atoms ($S1_{SAM}$ – $C3_{SAM}$ – $C7_{indole}$) should be in the range of 170 to 180°. Third, we suppose that an orthogonal orientation of the reactive indole ring plane to the cofactor reactive methyl group should be necessary to allow a maximal exposure of the pyrrole ring to the sulfonium ion to ensure an ideal stabilization of the positive charge of the transition state. The orientation of the reactive indole with respect to the cofactor S1 – C3 bond can be monitored by the $C3_{SAM}$ - $C7_{indole}$ - $N9_{indole}$ angle (Figure 12a). Thus, we considered all binding poses that also showed a $C3_{SAM}$ - $C7_{indole}$ - $N9_{indole}$ angle in the range of 85 to 95° (Figure 12b). Fourth, we suggest

that both substrate C8 and N12 atoms involved in the pyrroloindole ring formation should ideally be on the same side of the DKP ring, i.e., within a maximal distance of ~3.5 Å from one another (Figure 12c) to ensure the cyclization of the reaction intermediates after methyl transfer. Fifth, we assume that, due to its catalytic role, the Y127 side chain should be within 6 to 8 Å away from the reaction center. This distance cutoff is a conservative estimate based on the distances measured between the sulfur atom of the SAM and the hydroxyl moiety of the equivalent tyrosine residue in the crystal structures of other methyltransferases sharing a similar fold (Figure 12e-f).

QM calculations

Optimized geometries of reactive conformations of methylated reaction intermediates were modeled using GFN2-xTB[6] (tight accuracy). To account for the protein environment, the cWW were bound to a truncated binding pocket model (575 atoms). Starting conformations were extracted from individual MD snapshots. To account for missing residues and model a crude extended protein environment, the CPCM implicit solvation[7] model ($\varepsilon = 4$) was used in the ORCA 5.0.3 software[3]. The positions of the SAM cofactor and all backbone heavy atoms were constrained during geometry optimization.

Binding free energy decomposition

MM-GBSA calculations and a per-residue energy decomposition[32, 33] were performed with the GB^{OBC} model[34] using the MMPBSA.py script[35] to compute the effective binding free energy (ΔG_{res}) per residue. Based on former QMMM studies[10, 31] on other methyltransferases, these calculations were performed on a limited ensemble of conformations identified as reactant states. A reactant state was defined as all cWW substrate conformers showing a maximum distance of 3.5 Å between the substrate C7 atom and the SAM methyl group (C3 atom) as well as a C7-C3-

S1 angle value ranging between 160 and 180°. Calculations were performed using an ionic concentration of 0.1 M. ΔG_{res} values were averaged over seven independent simulation replicas of 200 ns each after the exclusion of the first 10 ns.

Residue conservation analysis

The WebLogo server[36, 37] was used to calculate and visualize residue conservation based on a multiple sequence alignment of 33 homologous sequences to StspM1 (Genbank: MK573553) built using the MAFFT-L-INS-i algorithm[38] in the ConSurf webserver.[39, 40] Homologous sequences showing a sequence identity to StspM1 between 40% and 90% were searched in the UniRef90 database[41] using psi-BLAST.[42] Coevolution-based contact predictions were performed using the NeBcon webserver.[43]

Biological methods

Protein sequences

Streptomyces sp. HPH0547 methyltransferase (StspM1) (GenBank: MK573553)

MSSETATPADPYTNLADSYDRLAEWAVTCQKESPRDRVADFLQTFWQSQQRPVRTVL
EICCGTGLMLGELARRGYAVTGLDRSAAMLERARRRLGEETTLIHAALPHIPAEAGPFD
AVVSAAGGLNYLPEEQISATFAAVARALPAGGTFTFDVFGRGFFRKFFDSSAPRVMALE
LDDIAYIWTFTASPEAPFVDMAYTQFTPAPAADGGEPPFLRTRDLHRYYPLPHTTVRRLA
AEHGFTDTKAYDNYSTDPSGPDSLYDTWTMVRSSSLEHHHHHH

Chloracidobacterium thermophilum halide methyl transferase (CtHMT) (GenBank: AEP12557.1)

MGHHHHHHAENLYFQGSGLGMDADTASFWEEKYRADLTAWDRGGVSPALEHWLA
EGALKPGRILIPGCGYGHEVLALARRGFEVWGLDIALTPVRRLQEKLAQAGLTAHVVEG
DVRTWQPEQPFDAVYEQTCLCALSPEDWPRYEAQLCRWLRPGGRLFALWMQTDRPGG
PPYHCGLEAMRVLFALERWRWVEPPQRTVPHPTGFFE YAAILERLV

Vectors, bacterial strains, and media

The gene sequence for the methyl transferase StspM1 was constructed synthetically in a pET21a vector (GenScript, USA). The gene sequence of the HMT was provided by Prof. Seebeck (University Basel, Institute for organic chemistry) on a pET28a(+) vector. For protein expression, *E. coli BL21(DE3) Gold* or *E. coli Δmtn* strains (provided by Prof. Seebeck) were chosen. As medium for precultures, LB (lysogeny broth) liquid medium (10 g·L^{-1} tryptone, 5 g·L^{-1} yeast

extract, 2 g·L^{-1} sodium chloride) or for gene expression TB (terrific broth) liquid medium (Carl Roth, Karlsruhe, Germany: 12 g·L^{-1} casein, 24 g·L^{-1} yeast extract, 12.54 g·L^{-1} K_2HPO_4, 2.3 g·L^{-1} KH_2PO_4, 4 mL·L^{-1} glycerol) with kanamycin or ampicillin as antibiotic with a final concentration of 100 µg·mL^{-1} were used. The cultivation media were prepared with distilled water and afterwards sterilized by autoclaving.

Transformation

Cells of the desired strain were transformed via heat shock procedure with the desired plasmid: To 100 µL competent cells 100 ng plasmid DNA were added and incubated 30 min on ice. For the heat shock, the cells were heated for 90 sec at 42 °C in a water bath. 700 µL LB-medium without antibiotics were added and slowly mixed by rotation for 1 h at 37 °C. Afterward, the transformed cells were cultivated overnight on LB-medium agar plates (supplemented with appropriate antibiotic) at 37 °C.

Gene expression

A colony from the transformed cells with the desired plasmid was picked from the agar plate to inoculate a 5 mL preculture. After 16 h at 37 °C, the preculture was used to inoculate 500 mL TB medium supplied with appropriate antibiotic in a 1 L non-baffled flasks to the dilution of 1:100 and incubated at 37 °C and 130 rpm until an OD600nm of 0.5 was reached. To start the protein expression, the non-degradable inducer IPTG was added to the final concentration of 100 µM. The culture was transferred to a shaker at 25 °C and incubated at 130 rpm for 20 h. After this, the cells

were harvested via centrifugation at 5000 g and 4 °C for 40 min and the pellet was stored at −20 °C till further use.

Enzyme purification

The cell pellet was resuspended (0.2 g/mL) in lysis buffer (50 mM KPi, pH 7.5), lysed by sonication with an ultrasonic cell disruptor (Branson Sonifier II "Modell W-250", Heinemann) for 10 min and an amplitude of 35–40%. The lysates were centrifuged (4 °C, 9000 × rcf, 15 min) and the pellet was discarded. For purification, a Ni-NTA affinity chromatography (5 mL HiTrap HP column, Bio-Rad) and a flow rate of 3 mL/min were used. After equilibrating the column (5 CV, 50 mM KPi, 500 mM NaCl, pH 7.5) and loading the lysate, a washing step was performed (5 CV, 50 mM KPi, 500 mM NaCl, 80 mM ImH, pH 7.5). For elution of the protein (3 CV, 50 mM KPi, 500 mM NaCl, 160 mM ImH, pH 7.5), the imidazole concentration was increased. The protein-containing fractions were concentrated (Vivaspin 20, 10000 MWCO PES, Sartorius Stedim Biotech) and the imidazole was removed with a buffer exchange to the lysis buffer again. For storing, the concentrated enzyme solution was frozen in liquid nitrogen and kept at −20 °C until usage.

Size exclusion chromatography

Size exclusion chromatography was performed on a Superdex™ G200 HiLoad 16/600 column (Cytiva) at 25 °C and a flow rate of 0.75 mL/min using an ÄKTA Purifier device. The calibration for the analytic size exclusion chromatography was performed using a gel filtration marker kit for protein molecular weights 12000-200000 Da (Sigma) including blue dextran, α-amylase,

cytochrome C, alcohol dehydrogenase, BSA, and carbonic anhydrase with KPi buffer (10 mM, 150 mM NaCl, pH = 7.5) as mobile phase.

General procedure for Mtase glo assay

For measuring the activity of the methyltransferase the commercially available Mtase Glo assay (Promega) was used according to the manufacturer protocol. An ideal enzyme concentration of 1.5 µM was calculated and a SAH calibration curve was measured as described in the protocol. The luminescence was detected in a plate reader (Infinite plate reader, Tecan). All measurements were performed in triplicates in a 96 well format (Nunc™ 96-well plates (Nunclon®; polystyrene, flat bottom, white)). The reactions were performed at 45 °C and terminated after five, ten, or 15 min by adding 0.5 % TFA. The reaction rate was calculated by linear regression over the three time points and plotted against the substrate concentration. The kinetic parameters were determined by a non-linear regression using the Michaelis-Menten equation performed with the software Origin Pro 2017G (OriginLab Corporation, Northampton, MA, USA).

In vitro assay

For analytical reactions, a total volume of 1 mL in 1.5 mL Eppendorf tubes was chosen. The cWW substrate was dissolved in DMSO (100 mM). The reactions were performed in KPi buffer (pH 7.5, 50 mM) with an end concentration of 1 mM cWW substrate at 40 °C and 700 rpm. For experiments with pure enzyme, the desired amount of pure StspM1 and 3 mM SAM were added. For reaction with the coupled recycling system, the desired amounts of StspM1 lysate and HMT

lysate were added plus the desired amount of MeI (1 M stock solution in DMSO). The lysate was produced by sonication of the cells in lysis buffer (0.2 g/mL) with an ultrasonic cell disruptor (Branson Sonifier II "Modell W-250", Heinemann) for 10 min and an amplitude of 35–40%. The reactions were stopped by addition of 20 % acetonitrile or 0.5 % TFA and analyzed by RP-HPLC (Jasco HPLC). As stationary phase, the hyperclone column (5µ ODS C18, 125*4 mm, 120 Å, Fa. Phenomenex) was used. As mobile phase A: Acetonitril + 0.1% formic acid (v/v) and B: water + 0.1% formic acid (v/v) were used with the following gradient: 0-2 min: 10:90 (A:B), 2-15 min: 10% A to 50% A, 15-25 min: 50% A to 95% A, 25-30 min: 95:5 (A:B), 30-35 min: 10:90 (A:B). Flow rate was set to 0.8 mL/min and the column temperature was kept at 25 °C. As detection wavelengths, 220 nm and 284 nm were chosen. 30 µL of each sample were injected.

HMT activity

For measuring the HMT lysate activity a total volume of 1 mL in 1.5 mL Eppendorf tubes was chosen. SAH and MeI as substrates were dissolved in DMSO. The reactions were performed in KPi buffer (pH 7.5, 50 mM) with an end concentration of 1 mM SAH and 10 mM MeI at 40 °C and 700 rpm. The desired amount of HMT lysate (1% - 3%) were added additional to the substrates. The lysate was produced by sonication of the cells in lysis buffer (0.2 g/mL) with an ultrasonic cell disruptor (Branson Sonifier II "Modell W-250", Heinemann) for 10 min and an amplitude of 35–40%. The reactions were stopped by addition of 0.5% TFA after 3, 6, and 9 min and analyzed by RP-HPLC (Jasco HPLC). As stationary phase, the hyperclone column (5µ ODS C18, 125*4 mm, 120 Å, Fa. Phenomenex) was used. As mobile phase A: Acetonitrile and B: 10 mM NaH_2PO_4 buffer (5 mM heptane sulfonic acid, pH 3.5) were used with the following

gradient: 0-5 min: 5:95 (A:B), 5-20 min: 20:80 (A:B), 20-24 min: 80:20 (A:B), 24-35 min: 95:5 (A:B), 30-35 min: 10:90 (A:B). Flow rate was set to 0.8 mL/min and the column temperature was kept at 25 °C. As detection wavelength, 260 nm was chosen. 30 µL of each sample were injected.

Design of experiment

A response surface design of experiment approach was chosen with the methyl iodide concentration, the StspM1 lysate amount, and the CtHMT lysate as factors and the conversion after 24 h as response. The Design Expert 12 (12.0.7.0) was used to create the conditions (see Table 1). Two additional negative controls were measured. The reactions were carried out randomized under the in vitro assay conditions.

Enzymatic preparative scale reaction

4.3 g of CtHMT cell pellet and 9.13 g of StspM1 cell pellet were lysed in 21.5 mL / 45.7 mL reaction buffer (50 mM KPi, pH 7.5). The lysate was produced by sonication of the cells in lysis buffer (0.2 g/mL) with an ultrasonic cell disruptor (Branson Sonifier II "Modell W-250", Heinemann) for 10 min and an amplitude of 35–40% two times. After centrifugation (4 °C, 10000 × rcf, 20 min) the supernatant served as lysate. The lysates were transferred into a glass bottle and filled up with buffer to a total volume of 134.4 mL. The cWW substrate (50 mg, 0.13 mmol) was added in DMSO (1.34 mL) as well as MeI (100 µL, 1.61 mmol). The reactions were performed in KPi buffer (pH 7.5, 50 mM) with an end concentration of 1 mM cWW substrate at 40 °C and 300 rpm. The reaction was monitored by TLC and quenched after 24 h by addition of NaOH solution (500 mM end concentration). After extraction with ethyl acetate (three times), the organic

phase was dried with $MgSO_4$ and concentrated under reduced pressure. The final products were purified by column chromatography (ethyl acetate).

Immobilization protocol

0.2 mL of the homogeneous suspension of Ni-NTA Agarose were transferred in 2 mL Eppendorf tube. The resin was settled by gravity and the supernatant was removed. 1 mL Lysis Buffer were added and gently resuspended. The supernatant was removed after settling down. 2 mL cleared lysate were added to the equilibrated Ni-NTA Agarose resin and incubated at 4 °C for 40 min on an end-over-end shaker. The supernatant was removed and 1 mL washing solution (Lysis Buffer + 80 mM ImH (StspM1 or 40 mM ImH (HMT)) were added and incubated at 4 °C for 10 min on an end-over-end shaker. After removing the supernatant, 2 mL lysis buffer were added again and the mixture was ready to use.

Enzymatic preparative scale reaction with immobilized enzyme

4.3 g of CtHMT cell pellet and 9.13 g of StspM1 cell pellet were lysed in 43 mL / 91.4 mL reaction buffer (50 mM KPi pH 7.5). The lysate was produced by sonication of the cells in lysis buffer (0.2 g/mL) with an ultrasonic cell disruptor (Branson Sonifier II "Modell W-250", Heinemann) for 10 min and an amplitude of 35–40% two times. After centrifugation (4 °C, 10000 × rcf, 20 min) the supernatant served as lysate. The immobilization of the enzymes was performed with the above protocol. The immobilized enzymes on the Ni-NTA material were transferred into a glass bottle and filled up with buffer to a total volume of 134.4 mL. The cWW substrate (50 mg,

0.13 mmol) was added in DMSO (1.34 mL) as well as MeI (100 µL, 1.61 mmol). The reactions were performed in KPi buffer (pH 7.5, 50 mM) with an end concentration of 1 mM cWW substrate at 40 °C and 300 rpm. The reaction was monitored by TLC and quenched after 24 h or 48 h by addition of ammonium thiosulfate (50 equiv). The Ni-NTA material was removed *via* filtration and washed with buffer and ethyl acetate. After extraction with ethyl acetate (three times), the organic phase was dried with $MgSO_4$ and concentrated under reduced pressure. The final products were purified by column chromatography (ethyl acetate).

Mutagenesis

Mutants were obtained by mutagenesis PCR amplification using the StspM1-pET21a vector as a template. The PCR mixture contained 2.5 µL of each primer (10 µM), 1 µL template vector (10 ng/µL), dNTP (10 mM) and 0.5 µL Phusion DNA-polymerase in a total volume of 50 µL Phusion-GC-buffer (Thermo Fisher Scientific).

Table S1. Primer for mutagenesis Y127A and Y127F

**Primer**	**Sequence**
MHA StspM1_Y127A Fw	CGGAGGACTGAACGCCCTGCCGGAGGAAC
**MHA StspM1_Y127F Fw**	CGGAGGACTGAACTTCCTGCCGGAGGAAC
**MHA StspM1_Y127 Rev**	GCGGCGCTGACGACGG

Table S2. Conditions of PCR reaction

Step	Cycles	Temperature [°C]	Time
Initial denaturation	1	98	30 s
Denaturation		98	10 s
Annealing	30	60	30 s
Extension		72	15-30 s/kb
Final extention	1	72	8 min

After digestion of the mixture with FastDigest DpnI (Thermo Fisher Scientific). for 1 h at 37°C, a preparative agarose gel was performed. The corresponding bands were extracted with the innuPREP DOUBLEpure KIT and the construct was ligated with the T4 DNA ligase (Thermo Fisher Scientific) at 16 °C for 24 h. The PCR product was used to transform *E.coli* DH5 alpha cells via heat shock. After overnight culturing, the plasmid was isolated using a plasmid isolation kit (Innuprep, Analytik Jena). After verification of the correct sequence, this plasmid was used for further protein expression. The mutants were tested under *in vitro* assay conditions showing no activity.

Chemical methods

General information

All used chemicals were received from commercial sources not further purified. For inert conditions, THF was used as a solvent, dried using an MB-SPS-800 solvent purification system. Flame-dried glassware and an inert atmosphere (N_2 or Ar atmosphere) were used to ensure oxygen and water exclusion. For reaction control, thin layer chromatography with TLC-foil Polygram SilG/UV254 by Macherey-Nagel as a stationary phase and ethyl acetate, if not stated differently, as mobile phase was used. The TLC plates were stained using a cerium-molybdate-solution [10 g $Ce(SO_4)2\cdot4\ H_2O$, 25 g phosphomolybdic acid, 60 mL conc. H_2SO_4, 940 mL H_2O]. For purification, preparative column chromatography with silica gel (0.040−0.063 mm) by Merck as stationary phase and ethyl acetate as mobile phase were used. The optical rotation of synthesized compounds was measured using a JASCO P-2000 polarimeter. A Bruker Avance DRX 600 NMR was used for measuring the ^{1}H, ^{13}C and 2D NMR (COSY, HSQC, HMBC) spectra of the samples dissolved in deuterated chloroform or deuterated methanol. The ROSEY NMR sprctra were measured at the AVANCE III console NMR sprectrometer (Bruker) at the IBI-7 at the Research Center Jülich. The solvent peak or the internal standard (tetramethylsilane) peak were used as reference peak. For measuring IR spectra, a PerkinElmer SpectrumTwo spectrometer was used. High-resolution ESI mass spectra were measured by the HHU Center of Molecular and Structural Analytics at the Heinrich-Heine-Universität Düsseldorf using an MDS SCIEXQ Model Trap 4000 mass spectrometer.

Extinction coefficient

The absorption was measured with the Nanodrop 2000c (Thermo Scientific) in a concentration range from 0.2 mM to 1 mM in Water:Acetonitril in a 4:1 ratio in dublicates. The path length of the system is 0.1 cm.

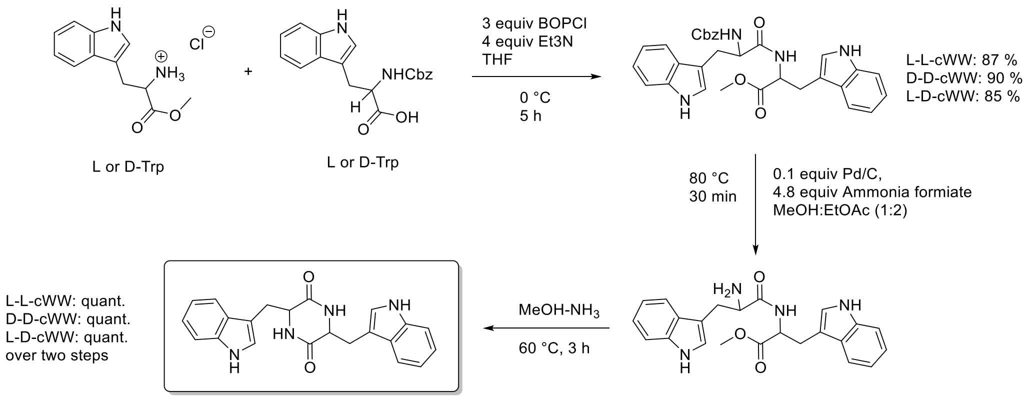

Scheme S1. Synthesis of cWW substrates with reaction conditions and yields.

*Synthesis of Methyl ((Benzyloxy)carbonyl)-tryptophyl-tryptophanate (**S3**)*[44]

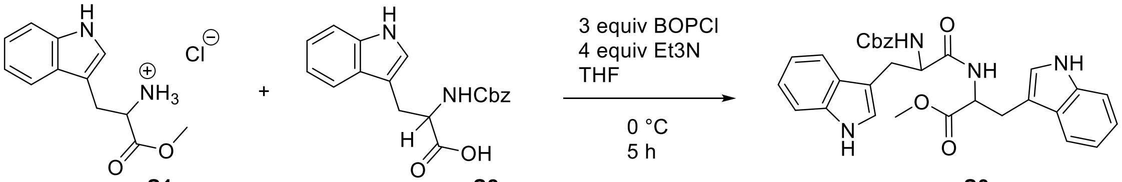

S1 **S2** **S3**

Benzyl chloroformate-protected L-tryptophan **S2** (1.0 equiv) and methylated L-tryptophan **S1** (1.0 equiv) were dissolved in dry THF (0.25 mmol·mL^{-1}) and cooled to 0 °C. After 10 minutes, bis(2-oxo-3-oxazolidinyl)phosphinic chloride (BOPCl) (3.0 equiv) was added and the reaction stirred for 2 h at 0 °C and then over night at 21 °C. For quenching, 20 mL water were added, and the product was extracted with ethyl acetate (3 x 20 mL). The merged organic phases were washed

with brine and dried over $MgSO_4$. After filtration, theThe solvent was removedunder reduced pressure. The crude product **S3** was purified via column chromatography on silica using ethyl acetate as eluent.

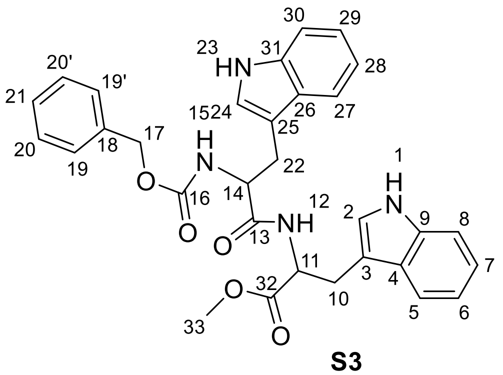

S3

LL-**S3**

Starting from 800 mg (2.36 mmol) *L*-**S2** and 662 mg (2.36 mmol) *L*-**S1** 1.096 g *LL*-**S3** (2.15 mmol, 91%) were obtained.

^{1}H NMR (600 MHz, MeOD) δ 7.57 (d, J = 7.9 Hz, 1H), 7.39 (d, J = 8.0 Hz, 1H), 7.37 – 7.22 (m, 8H), 7.14 – 7.05 (m, 4H), 7.04 – 6.93 (m, 3H), 5.06 – 4.97 (m, 2H, 17-H), 4.71 (t, $^3J_{11,10}$ = 6.4 Hz, 1H, 11-H), 4.45 (t, $^3J_{14,22}$ = 6.9 Hz, 1H, 14-H), 3.58 (s, 3H, 33-H), 3.25 – 3.14 (m, 3H, H-22b, H-10), 3.08 (dd, $^2J_{22a,22b}$ = 14.6 Hz, $^3J_{22a,11}$ = 7.6 Hz, 1H, H-22a).

^{13}C NMR (151 MHz, MeOD) δ 174.22 (C-13), 173.48 (C-32), 158.11 (C-16), 138.12, 138.04 (C-9, C-33), 137.95 (Ar-H), 129.42 (C-19, C19‘ or C-20, C-20‘), 128.90, 128.69 (C-Ar), 128.66 (C-19, C19‘ or C-20, C-20‘), 124.73, 124.60, 122.43, 122.40, 119.86, 119.36, 119.11, 112.31, 112.27 (C-Ar), 110.79 (C-25), 110.24 (C-3), 67.60 (C-17), 57.14 (C-14), 54.78 (C-11), 52.64 (C-33), 29.04 (C-22), 28.40 (C-10).

IR: IR (ATR): ṽ [cm^{-1}] = 3379 (m), 3304 (m), 1742 (s), 1694 (s), 1651 (vs), 1436 (w), 1265 (s), 1248 (s), 1219 (s), 734 (vs), 732 (vs), 642 (m), 608 (m), 560 (m), 460 (w).

MS: MS (ESI, 60 eV): m/z (%) = 539.23 [M+]

Optical rotation: $[\alpha]_D^{20}$ = -12.4 (*c* 1.0, DMSO)

Melting point: 186.2 °C

DD-**S3**

Starting from 569 mg (1.68 mmol) *D*-**S2** and 167 mg (1.68 mmol) *D*-**S1** 812 mg *DD*-**S3** (1.51 mmol, 90%) were obtained.

^{1}H NMR (600 MHz, MeOD) δ 7.58 (d, J = 7.9 Hz, 1H), 7.39 (d, J = 8.0 Hz, 1H), 7.37 – 7.22 (m, 8H), 7.14 – 7.05 (m, 4H), 7.04 – 6.93 (m, 3H), 5.06 – 4.97 (m, 2H, 17-H), 4.71 (t, $^3J_{11,10}$ = 6.4 Hz, 1H, 11-H), 4.46 (t, $^3J_{14,22}$ = 6.9 Hz, 1H, 14-H), 3.58 (s, 3H, 33-H), 3.25 – 3.14 (m, 3H, H-22b, H-10), 3.08 (dd, $^2J_{22a,22b}$ = 14.6 Hz, $^3J_{22a,11}$ = 7.6 Hz, 1H, H-22a).

^{13}C NMR (151 MHz, MeOD) δ 174.21 (C-13), 173.48 (C-32), 158.12 (C-16), 138.15, 138.06, 137.97 (C-9, C-33, C-18), 129.43 (C-19, C19‘ or C-20, C-20‘), 128.91, 128.70 (C-Ar), 128.67 (C-19, C19‘ or C-20, C-20‘), 124.73, 124.60, 122.44, 122.40, 119.86, 119.84, 119.36, 119.10, 112.30, 112.27 (C-Ar), 110.80 (C-25), 110.25 (C-3), 67.60 (C-17), 57.15 (C-14), 54.79 (C-11), 52.64 (C-33), 29.04 (C-22), 28.40 (C-10).

IR: IR (ATR): ṽ [cm^{-1}] = 3379 (m), 3304 (m), 1742 (s), 1694 (s), 1651 (vs), 1436 (w), 1219 (s), 734 (vs), 732 (vs), 643 (m), 608 (m), 560 (m).

MS: MS (ESI, 60 eV): m/z (%) = 53923 [M+]

Optical rotation: $[\alpha]_D^{20}$ = 8.0 (*c* 0.8, DMSO)

Melting point: 184.1 °C

LD-**S3**

Starting from 100 mg (0.46 mmol) *D*-**S2** and 155 mg (0.46 mmol) *L*-**S1** 210 mg *LD*-**S3** (0.39 mmol, 85%) were obtained.

^{1}H NMR (600 MHz, MeOD) δ 7.52 (d, $^3J_{30,29}$ = 7.9 Hz, 1H, 30-H), 7.43 (d, $^3J_{8,7}$ = 7.9 Hz, 1H, 8-H), 7.36 – 7.24 (m, 7H), 7.13 – 7.06 (m, 3H), 7.02 – 6.96 (m, 2H), 6.93 (s, 1H, H-24), 6.84 (s, 1H, H-2), 5.08 – 4.97 (m, 2H, 17-H), 4.68 (t, $^3J_{11,10}$ = 6.5 Hz, 1H, 11-H), 4.46 (t, $^3J_{14,22}$ = 7.0 Hz, 1H, 14-H), 3.62 (s, 3H, 33-H), 3.18 (dd, $^2J_{22b,22a}$ = 14.6, $^3J_{22b,11}$ = 6.3 Hz, 1H, 22b-H), 3.13 – 3.04 (m, 3H, 10-H), 2.99 (dd, $^2J_{22a,22b}$ = 14.5 Hz, $^3J_{22a,11}$ = 7.7 Hz, 1H, 22a-H).

^{13}C NMR (151 MHz, MeOD) δ 174.26 (C-13), 173.67 (C-32), 160.49 (C-16), 138.14, 138.03, 138.00 (C-9, C-33, C-18), 129.43 (C-19, C19‘ or C-20, C-20‘), 128.92, 128.73, 128.60, 124.64, 124.57, 122.48 (C-24), 122.40 (C-2), 119.92, 119,86, 119.38, 119.37, (C-8, C-13), 119.13, 112.34, 112.27, 110.86 (C-25), 110.23 (C-3), 67.62 (C-17), 57.29 (C-14), 54.61 (C-11), 52.68 (C-33), 29.27 (C-22), 28.32 (C-10).

IR: IR (ATR): ṽ [cm^{-1}] = 3407 (m), 3302 (m), 1740 (s), 1690 (s), 1653 (vs), 1538 (m), 1268 (s), 1251 (s), 1048 (m), 744 (vs), 692 (m), 600 (m), 552 (m), 462 (w).

MS: MS (ESI, 60 eV): m/z (%) = 539.23 [M+]

Optical rotation: $[\alpha]_D^{20}$ = 1.2 (*c* 1.0, DMSO)

Melting point: 209.8 °C

*Synthesis of Cyclo-tryptophan-tryptophan diketopiperazine **7** (cWW)*[44, 45]

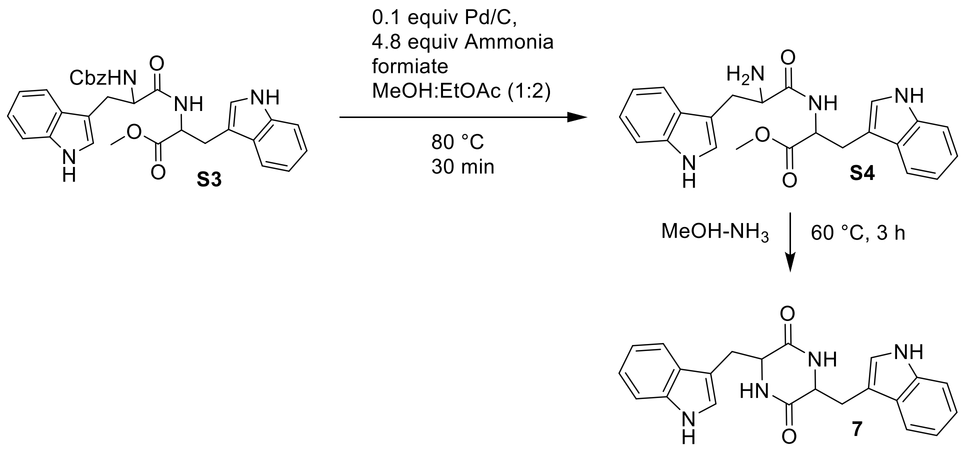

The Methyl((benzyloxy)carbonyl)-tryptophyl-tryptophanate **S3** was dissolved in MeOH/ethyl acetate (1:2) (0.05 mmol·mL^{-1}) under nitrogen atmosphere. Ammonium formate (4.8 equiv) and 10% Pd/C (0.1 equiv.) were added and the reaction mixture was stirred at reflux for 30 minutes. After filtration of the Pd/C, the filtrate was evaporated under reduced pressure and the crude product was used without further purification for the next step. The deprotected crude product was dissolved in 7 N methanolic ammonia (0.05 mmol·mL^{-1}) and stirred at 60 °C for 4 h. The solvent was removed under reduced pressure. The product appeared as white solid without further purification needed.

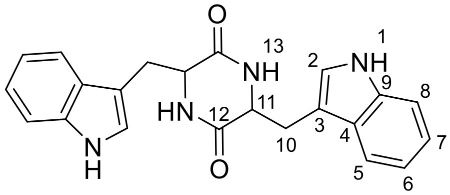

7

7a (*LL*-cWW)

Starting from 598 mg (1.11 mmol) *LL*-**S3** 409 mg **7a** (1.11 mmol, quant) were obtained.

Scale: 409 mg, 1.10 mmol (quant.)

^{1}H NMR (600 MHz, MeOD) δ 7.47 (d, $^3J_{8,7}$ = 8.0 Hz, 2H, H-8), 7.32 (d, $^3J_{5,6}$ = 8.1 Hz, 2H, H-5), 7.11 (dd, $^3J_{7,8}$ = 8.1, $^3J_{7,6}$ =6.9 Hz, 2H, H-7), 7.03 (dd, $^3J_{6,7}$ = 8.0, $^3J_{6,5}$ = 7.0 Hz, 2H, H-6), 6.49 (s, 2H, H-2), 4.06 (dd, $^3J_{11,10a}$ = 3.8 Hz, $^3J_{11,10b}$ = 7.2 Hz, 2H, H-11), 2.94 (dd, $^3J_{10a,10a}$ = 14.8, $^3J_{10a,11}$ = 3.8 Hz, 2H, H-10a), 2.19 (dd, $^3J_{10b,10a}$ = 14.8, $^3J_{10b,11}$ = 7.3 Hz, 2H, H-10b).

^{13}C NMR (151 MHz, MeOD) δ 169.71 (C-12), 138.05 (C-9), 128.61 (C-4), 125.90 (C-2), 122.52 (C-7), 120.07 (C-6), 119.68 (C-8), 112.41 (C-5), 109.45 (C-3), 56.86 (C-11), 31.39 (C-10).

IR: IR (ATR): ṽ [cm^{-1}] = 3266 (s), 3053 (vw), 2926 (w), 1704 (m), 1664 (vs), 1457 (s), 1360 (m), 1325 (m), 1230 (m), 1011 (w), 743 (s), 535 (vw).

MS: MS (ESI, 60 eV): m/z (%) = 373.17 (80) [M$^+$]

Optical rotation: $[\alpha]_D^{25}$ = *-77.0 (c* 0.4*, DMSO)*

Melting point: 254.3 °C

7b (*DD*-cWW*)*

Starting from 743 mg (1.38 mmol) *DD*-**S3** 514 mg **7b** (1.38 mmol, quant) were obtained.

Scale: 514 mg, 1.38 mmol (quant.)

^{1}H NMR (600 MHz, MeOD) δ 7.45 (d, $^3J_{8,7}$ = 8.0 Hz, 2H, H-8), 7.30 (d, $^3J_{5,6}$ = 8.1 Hz, 2H, H-5), 7.09 (dd, $^3J_{7,8}$ = 8.1, $^3J_{7,6}$ 7.0 Hz, 2H, H-7), 7.01 (dd, $^3J_{6,5}$ = 8.0, $^3J_{6,7}$ = 7.0 Hz, 2H, H-6), 6.46 (s, 2H, H-2), 4.05 (dd, $^3J_{11,10b}$ = 7.0, $^3J_{11,10a}$ = 3.8 Hz, 2H, H-11), 2.92 (dd, $^3J_{10a,11}$ = 14.4, $^3J_{10a,10b}$ = 3.8 Hz, 2H, H-10a), 2.17 (dd, $^3J_{10b,11}$ = 14.4, $^3J_{10b,10a}$ = 7.2 Hz, 2H, H-10b).

^{13}C NMR (151 MHz, MeOD) δ 169.73 (C-12), 138.09 (C-9), 128.64 (C-4), 125.90 (C-2), 122.54 (C-7), 120.09 (C-6), 119.69 (C-8), 112.42 (C-5), 109.50 (C-3), 56.91 (C-11), 31.40 (C-10).

IR: IR (ATR): ṽ [cm^{-1}] = 3266 (vs), 2923 (w), 1703 (m), 1663 (vs), 1457 (s), 1359 (s), 1325 (s), 1229 (m), 1091 (m), 1011 (w), 743 (vs), 524 (vw).

MS: MS (ESI, 60 eV): m/z (%) = 373.17 (80) [M$^+$]

Optical rotation: $[\alpha]_D^{25}$ *= 65.5 (c 0.3, DMSO)*

Melting point: 249.0 °C

7c (*LD*-cWW)

Starting from 598 mg (1.11 mmol) *LD*-**S3** 322 mg **7c** (0.80 mmol, quant) were obtained.

^{1}H NMR (600 MHz, MeOD) δ 7.43 (d, $^3J_{8,7}$ = 8.0 Hz, 2H, H-8), 7.28 (d, $^3J_{5,6}$ = 8.2 Hz, 2H, H-5), 7.07 (dd, $^3J_{7,6}$ = 8.2 Hz, $^3J_{7,8}$ = 7.0 Hz, 2H, H-7), 6.99 (t, $^3J_{6,5}$ = 8.0 Hz, $^3J_{6,7}$ = 7.0 Hz, 2H, H-

6), 6.44 (s, 2H, H-2), 4.03 (dd, $^{3}J_{11,10}$ = 7.1 Hz, 2H, H-11), 2.90 (dd, $^{3}J_{10a,11}$ = 14.4, 2H, H-10a), 2.14 (dd, $^{3}J_{10b,11}$ = 14.4 Hz, 2H, H-10b).

^{13}C NMR (151 MHz, MeOD) δ 169.72 (C-12), 138.08 (C-9), 128.63 (C-4), 125.90 (C-2), 122.53 (C-7), 120.09 (C-6), 119.69 (C-8), 112.42 (C-5), 109.50 (C-3), 56.90 (C-11), 31.40 (C-10).

IR: IR (ATR): ṽ [cm^{-1}] = 3304 (s), 3055 (vw), 2927 (w), 1670 (vs), 1459 (m), 1363 (w), 1318 (m), 1229 (w), 1088 (w), 1012 (vw), 744 (s)

MS: MS (ESI, 60 eV): m/z (%) = 373.17 (80) [M^{+}]

Melting point: 239.7 °C

Synthesis of methylated cWW references[46]

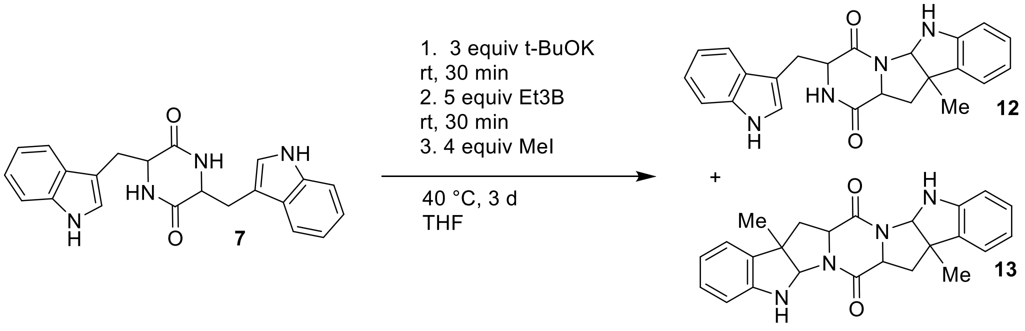

The cWW (1.0 equiv) was dissolved in dry THF (0.5 mmol·mL^{-1}) in an oven-dried Schlenk flask under argon atmosphere at room temperature. Afterward, t-BuOK (3.0 equiv) was added and the reaction mixture was stirred at room temperature. 30 min later, Et_3B (1 M in THF, 5.0 equiv) was added dropwise to the reaction mixture, and stirring continued for another 30 min. Methyl iodide (4.0 equiv) was added and the reaction was heated to 40 °C for 48 h. For quenching, NaOH solution was added to an end concentration of 500 mM. The aqueous phase was extracted with

ethyl acetate (3x 25 mL). The merged organic phases were washed with brine and dried over $MgSO_4$. The solvent was removed under reduced pressure. The crude product was purified via column chromatography on silica using ethyl acetate as eluent.

Starting from 90 mg (0.24 mmol) **a** 19 mg single methylated (0.05 mmol, 30%) and 10 mg (0.02 mmol) double methylated product were obtained.

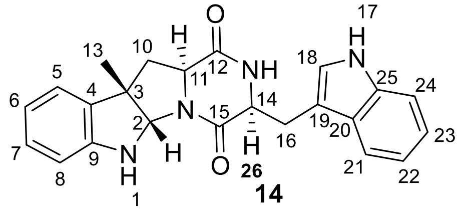

14

LL-cWW single methylated **14**

^{1}H NMR (600 MHz, $CDCl_3$) δ 8.35 (s, 1H, NH), 7.56 (d, $^3J_{24,23}$ = 8.0 Hz, 1H, 24-H), 7.36 (d, $^3J_{21,22}$ = 8.2 Hz, 1H, 21-H), 7.24 – 7.18 (m, 1H, H-Ar), 7.16 – 7.06 (m, 4H, H-Ar), 6.78 (t, J = 7.4, 1H, H-Ar), 6.63 (d, $^3J_{5,6}$ = 7.4 Hz, 1H, 5-H), 5.89 (s, 1H, NH), 5.26 (s, 1H, 2-H), 5.13 (s, 1H, NH), 4.30 (dd, $^3J_{14,16a}$ = 10.6 Hz, $^3J_{14,16b}$ = 3.8 Hz, 1H, 14-H), 3.95 (dd, $^3J_{11,10a}$ = 11.3 Hz, $^3J_{11,10b}$ = 6.1 Hz, 1H, 11-H), 3.67 (dd, $^2J_{16b,16a}$ = 15.2 Hz, $^3J_{16b,14}$ 3.8 Hz, 1H, 16b-H), 3.03 (dd, $^2J_{16a,16b}$ = 15.0 Hz, $^3J_{16a,14}$ = 10.6 Hz, 1H, 16a-H), 2.65 (dd, $^2J_{10b,10a}$ = 12.7 Hz, $^3J_{10b,11}$ = 6.1 Hz, 1H, 10b-H), 2.03 (dd, $^2J_{10a,10b}$ = 12.7 Hz, $^3J_{10a,11}$ = 11.3 Hz, 1H, 10a-H), 1.37 (s, 3H, 13-H).

^{13}C NMR (151 MHz, $CDCl_3$) δ 169.01 (C-15), 166.21 (C-12), 148.61 (C-9), 136.69 (C-25), 132.27 (C-4), 128.83 (C-18), 126.80 (C-20), 123.64 (C-18), 122.91, 122.88, 120.13, 119.71 (C-Ar), 118.67 (C-24), 111.68 (C-21), 109.57 (C-19), 109.52 (C-8), 81.57 (C-2), 59.16 (C-11), 54.88 (C-14), 51.71 (C-3), 40.93 (C-10), 27.33 (C-16), 24.47 (C-13).

IR: IR (ATR): ṽ [cm^{-1}] = 3316 (m), 1664 (vs), 1457 (s), 1321 (m), 1216 (w), 1097 (w), 1062 (w), 745 (s).

MS: MS (ESI, 60 eV): m/z (%) = 387.18 [M+]

Optical rotation: $[\alpha]_D^{20}$ = -140 (c 1.0, MeOH)

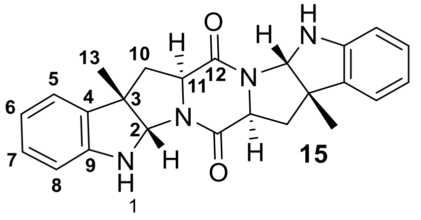

LL-cWW double methylated **15**

^{1}H NMR (600 MHz, $CDCl_3$) δ 7.05 (d, $^3J_{8,7}$ = 7.4 Hz, 1H, 8-H), 7.04 – 7.00 (m, 1H, 7-H), 6.74 (dd, $^3J_{6,5}$ = 7.4 Hz, $^3J_{6,7}$ = 7.4 Hz, 1H, 6-H), 6.52 (d, $^3J_{5,6}$ = 7.8 Hz, 1H, 5-H), 5.19 (s, 1H, 2-H), 5.04 (s, 1H, NH), 3.96 (dd, $^3J_{11,10b}$ = 11.0 Hz, $^3J_{11,10b}$ = 6.1 Hz, 1H, 11-H), 2.69 (dd, $^2J_{10b,10a}$ = 12.9 Hz, $^3J_{10b,11}$ = 6.2 Hz, 1H, 10b-H), 2.27 (dd, $^2J_{10a,10b}$ = 12.9 Hz, $^3J_{10a,11}$ = 11.3 Hz, 1H, 10a-H), 1.44 (s, 3H, 13-H).

^{13}C NMR (151 MHz, $CDCl_3$) δ 166.75 (C-12), 148.40 (C-9), 132.22 (C-4), 128.79 (C-7), 122.70 (C-8), 119.70 (C-6), 109.60 (H-5), 81.23 (C-2), 60.63 (C-11), 52.06 (C-3), 39.99 (C-10), 24.24 (C-13).

IR: IR (ATR): ṽ [cm^{-1}] = 3373 (m), 2960 (w), 1660 (vs), 1484 (m), 1424 (s), 1241 (w), 1165 (w), 1057 (w), 744 (s).

MS: MS (ESI, 60 eV): m/z (%) = 401.20 [M+]

Optical rotation: $[\alpha]_D^{20}$ = - 704 (c 0.1, MeOH)

$[\alpha]_D^{20}$ = - 741 (c 0.2, $CDCl_3$)

DD-cWW single methylated **16**

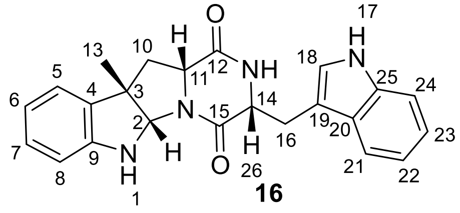

16

Starting from 90 mg (0.24 mmol) **7b** 20 mg single methylated product (0.05 mmol, 21%) were obtained.

^{1}H NMR (600 MHz, $CDCl_3$) δ 8.26 (s, 1H, NH), 7.58 (d, $^3J_{24,23}$ = 7.9 Hz, 1H, 24-H), 7.33 (d, $^3J_{21,22}$ = 8.2 Hz, 1H, 21-H), 7.24 – 7.18 (m, 1H, H-Ar), 7.15 – 7.07 (m, 3H, H-Ar), 7.02 – 6.98 (m, H, NH), 6.78 (t, J = 7.5 Hz, 1H, H-Ar), 6.61 (d, $^3J_{5,6}$ = 7.7 Hz, 1H, 5-H), 5.95 (s, 1H, NH), 5.41 (d, $^3J_{2,NH}$ = 3.1 Hz, 1H, 2-H), 4.37 (dd, $^3J_{14,16a}$ = 11.0 Hz, $^3J_{14,16b}$ = 3.8 Hz, 1H, 14-H), 4.35 – 4.30 (m, 1H, 11-H), 3.72 (dd, $^2J_{16b,16a}$ = 15.0 Hz, $^3J_{16b,14}$ = 3.7 Hz, 1H, 16b-H), 2.98 (dd, $^2J_{16a,16b}$ = 15.1 Hz, $^3J_{16a,14}$ =11.0 Hz, 1H, 16a-H), 2.36 (d, $^3J_{10,11}$ = 8.7 Hz, 2H, 10-H), 1.48 (s, 3H, 13-H).

^{13}C NMR (151 MHz, $CDCl_3$) δ 168.22 (C-15), 167.26 (C-12), 147.28 (C-9), 136.74 (C-25), 133.51 (C-4), 128.67 (C-18), 126.77 (C-20), 123.63, 122.83, 122.61, 120.06, 119.42 (C-Ar), 118.55 (C-24), 111.71 (C-21), 109.9´65 (C-19), 109.58 (C-5), 84.09 (C-2), 58.41 (C-14), 54.81 (C-11), 51.32 (C-3), 41.03 (C-10), 26.81 (C-16), 23.57 (C-13).

IR: IR (ATR): ṽ [cm^{-1}] = 3333 (m), 2925 (w), 1668 (vs), 1418 (s), 1343 (w), 1199 (m), 1101 (w) 745 (vs), 463 (w).

MS: MS (ESI, 60 eV): m/z (%) = 387.18 [M+]

Optical rotation: $[\alpha]_D^{20}$ = - 46 (c 1.0, MeOH)

Figure S1: Protein purification

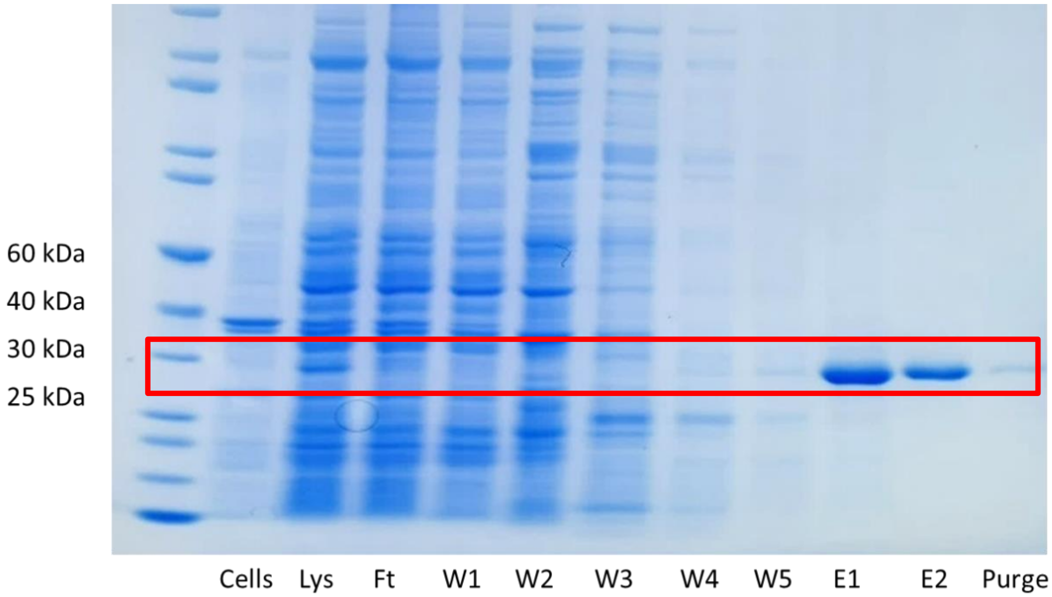

Figure S1. SDS gel of the purification of StspM1. 6.5 mg protein have been isolated from 4 g wet cells. As standard, the Page Ruler unstained protein ladder (0.02 – 0.05 µg/µL) has been used. For the resuspended cell-pellet (cells), lysate and flow through: 2 µL sample have been diluted in 10 µL water before applying on the gel; the other samples were applied undiluted. Lys =Lysate , Ft = Flow through, W = Washing fraction, E = Elution fraction.

Figure S2: Retention times of substrates and products + calibration + extinction coefficient

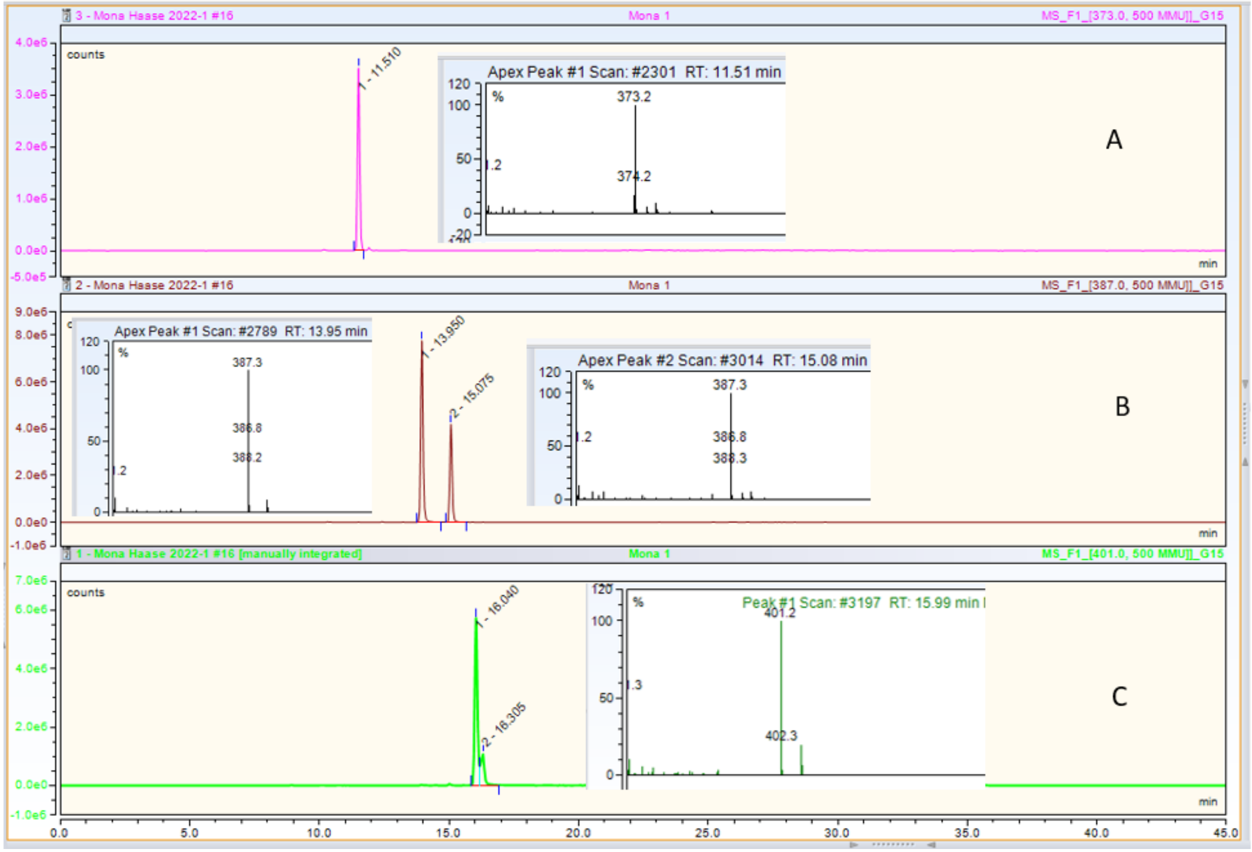

Figure S2. Extracted chromatograms of references measured with HPLC/MS. A: *LL*-cWW (**7a**), B: single methylated product of *LL*-cWW (**14**) (left) and *DD*-cWW (**16**) (right), C: double methylated *LL*-cWW (**15**).

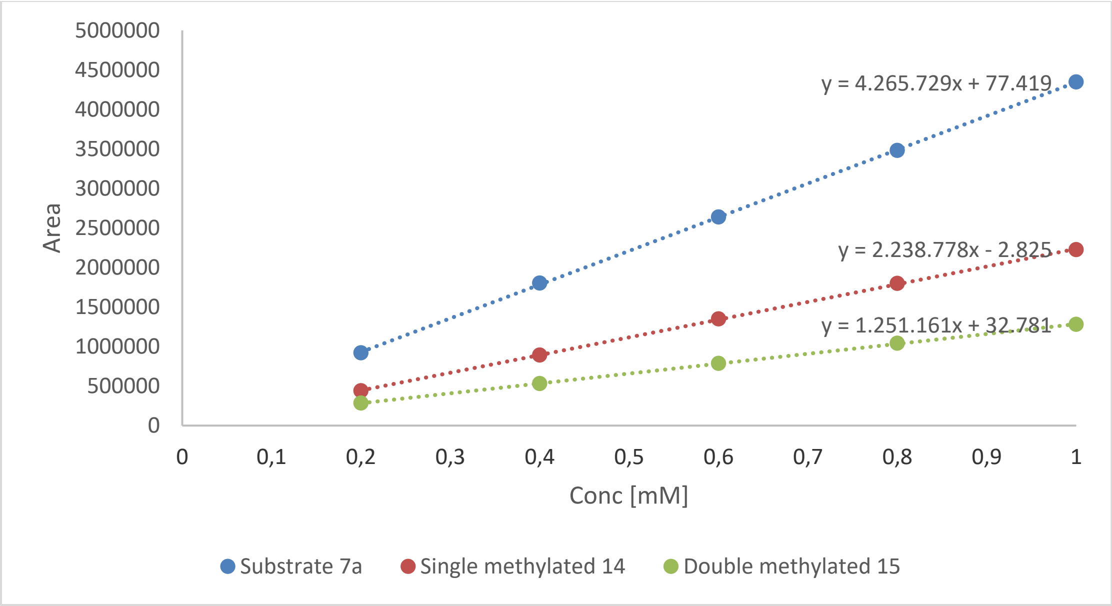

Figure S3. HPLC calibration of *LL*-cWW (**7a**), single methylated *LL*-cWW (**14**) and double methylated *LL*-cWW (**15**).

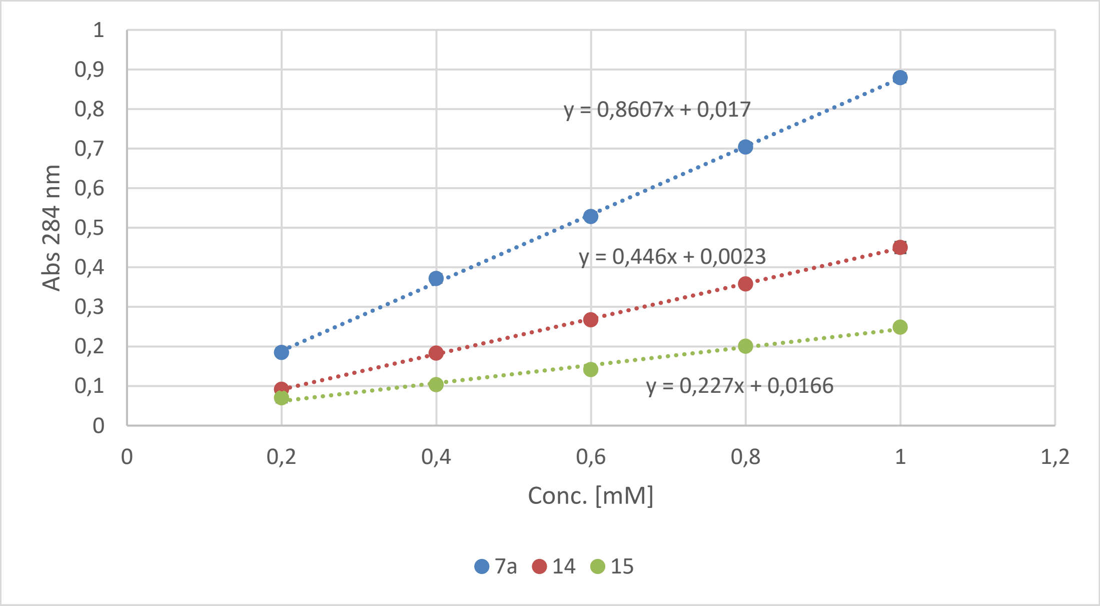

Figure S4. Determination of extinction coefficient of *LL*-cWW **7a**, single methylated *LL*-cWW **14** and double methylated *LL*-cWW **15**. The extinction coefficient of the substrate **7a** is 8607 $M^{-1}cm^{-1}$, of the single methylated product **14** 4460 $M^{-1}cm^{-1}$ and of the double methylated product **15** 2270 $M^{-1}cm^{-1}$.

Figures S5+6: Glo Assay calibration

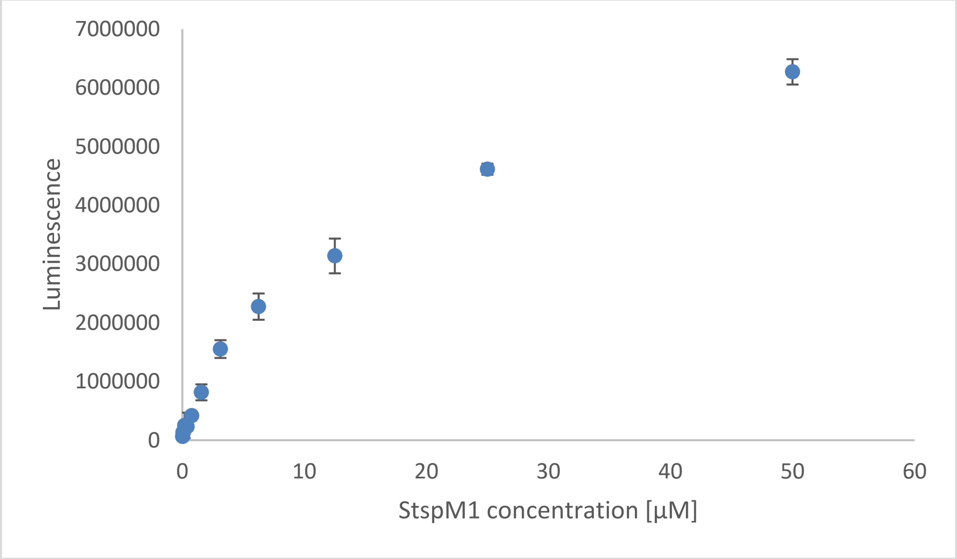

Figure S5. Determination of the ideal enzyme concentration with the Glo assay (Promega) tested with the substrate **7a**. Relative luminescence was measured for different StspM1 concentrations. Experiment was performed according to the manufacturer's protocol.

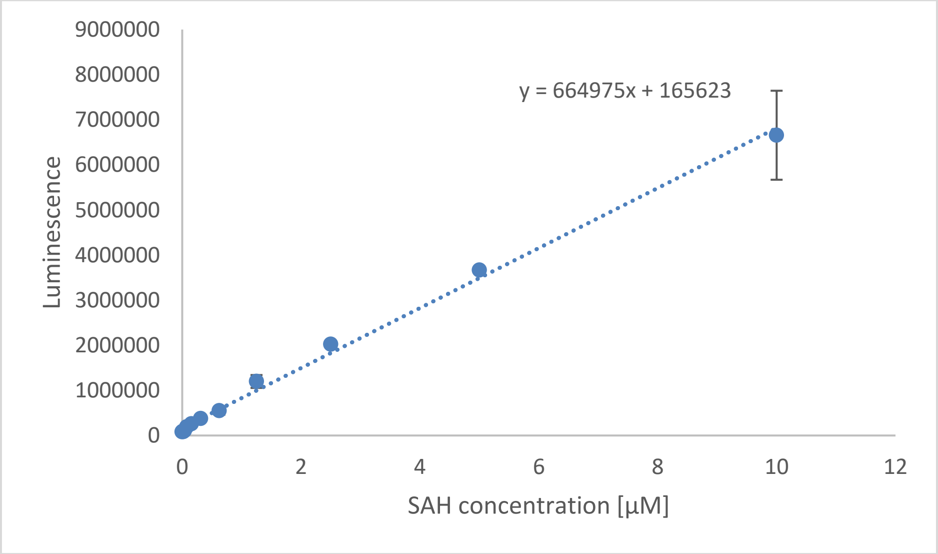

Figure S6. SAH calibration curve for the Glo assay (Promega). Relative luminescence was measured for different SAH concentrations. Experiment was performed according to the manufacturer's protocol.

Figures S7: Michaelis Menten Kinetics

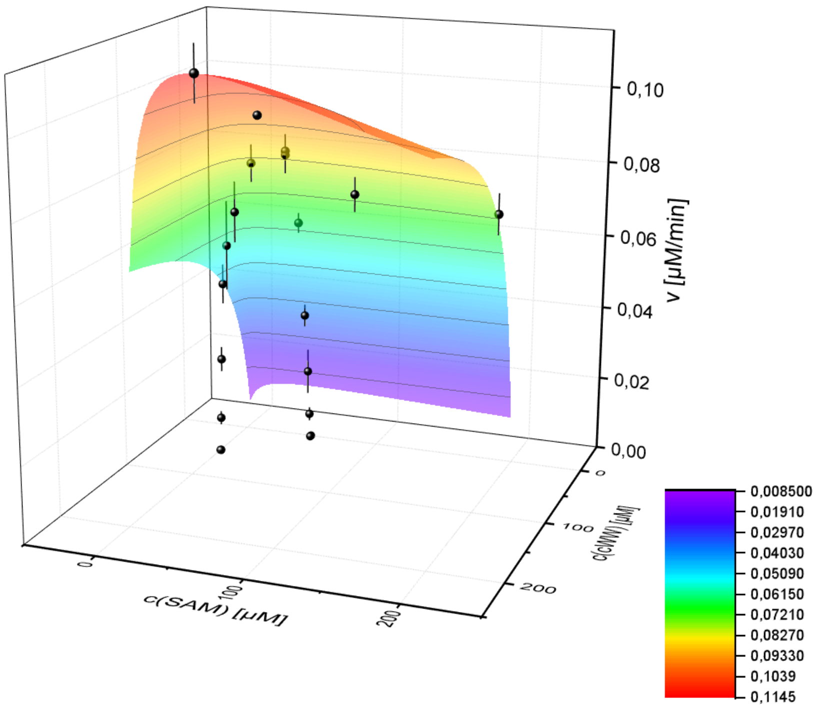

Figure S7. Enzyme kinetics (McMurray-Plot) with single methylated *LL*-cWW **14** and SAM as substrates. Kinetic values are shown in table S4.

Figure S8: Size Exclusion Chromatography (SEC) of StspM1

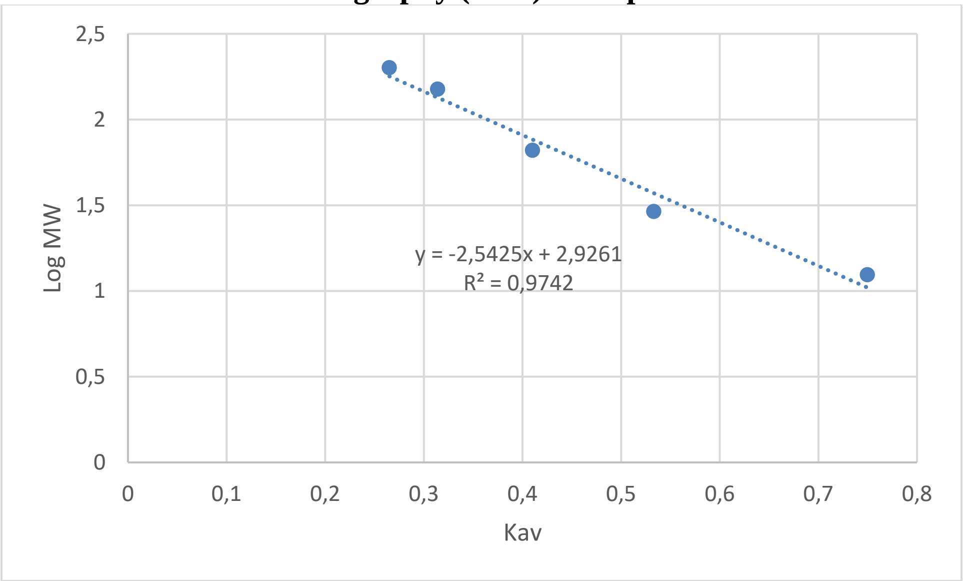

Figure S8. Results of SEC. Kav = partition coefficient (proportion of pores available to the molecule). With the elution volume of 78.4 mL, the MW of StspM1 was calculated to be 70.5 kDa.

Figures S9-14: Computational analysis

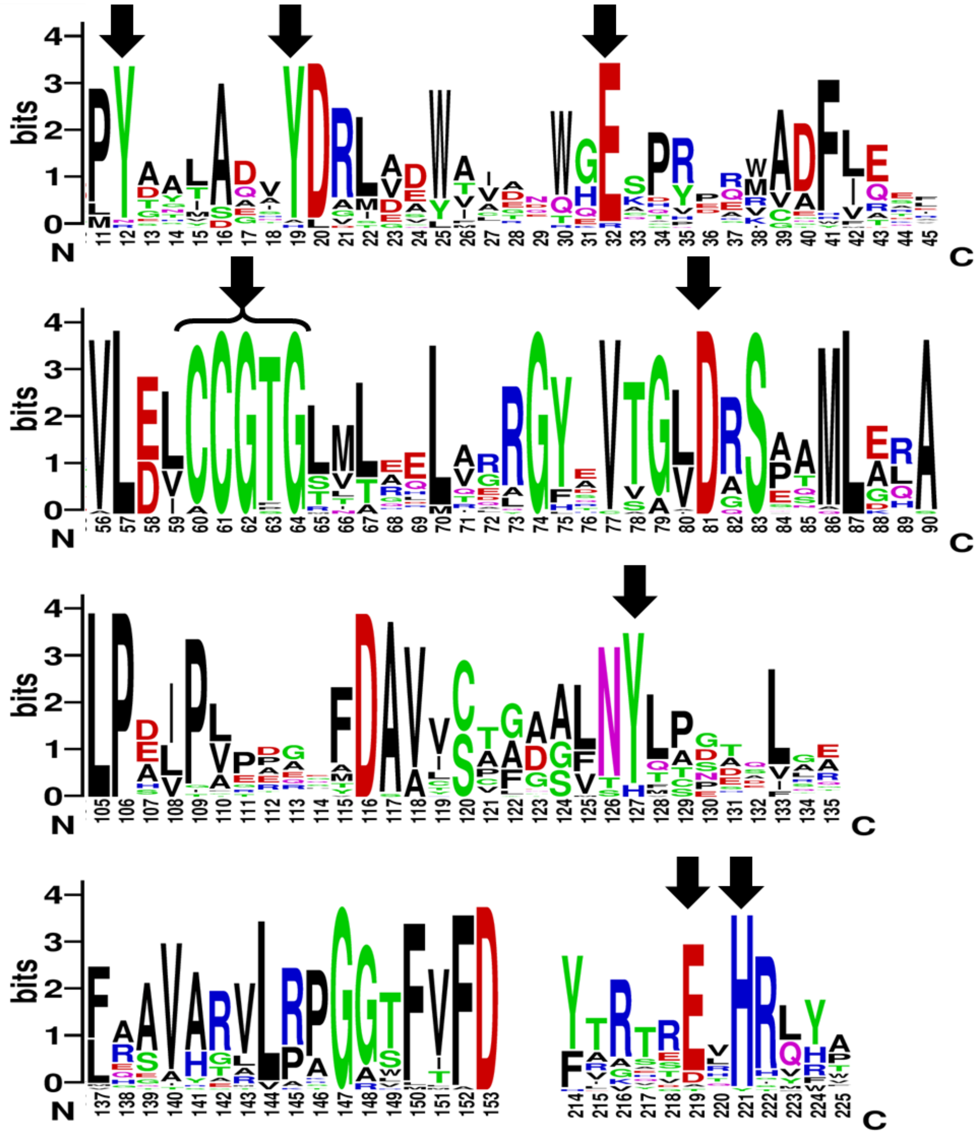

Figure S9. Conservation of key residues in StspM1 sequence homologs (for utilized sequences see supporting sequences_alignment file). The conservation score (in bits) was calculated from a multiple sequence alignment built using ConSurf[40] and processed using the WebLogo server.[36, 37] Essential residues mentioned in the discussion part are indicated by a black arrow. The curly bracket highlights the conserved CCGTG motif.

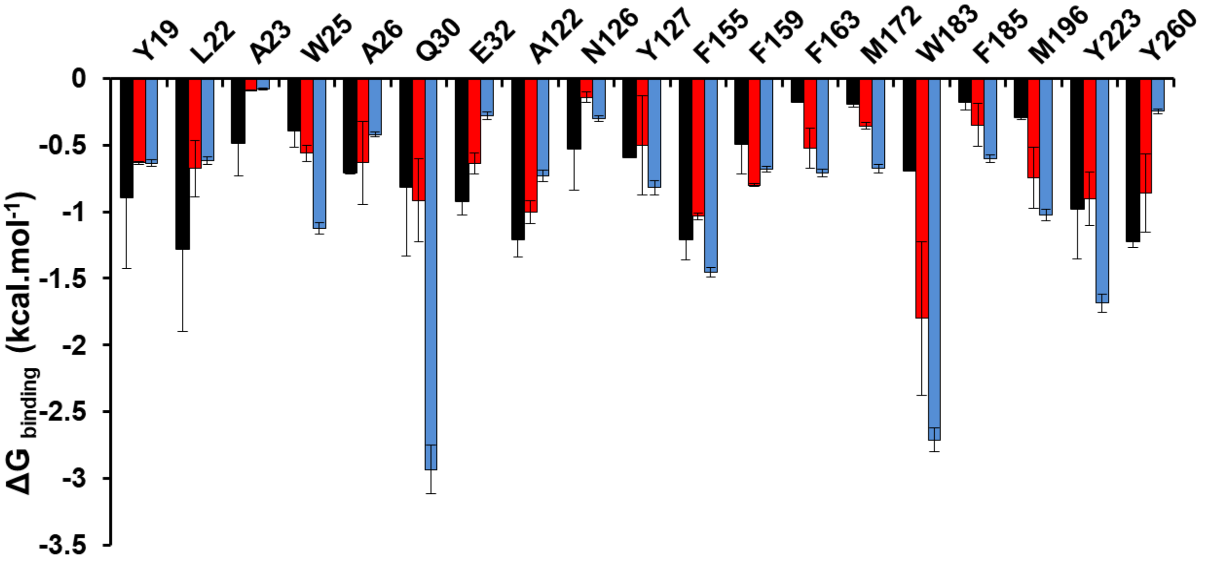

Figure S10. Residues contributing to effective binding energies of the unmethylated cWW over 7 x 200 ns of MD simulation. The black, red, and blue bars refer to the per-residue average MM-GBSA effective energy values for *LL*-cWW **7a**, *DD*-cWW **7b**, and *LD*-cWW **7c**, respectively. The average free energy values and their standard error were calculated over seven simulation replicas of ~ 200 ns length each. Both subunits in the StspM1 dimer were considered.

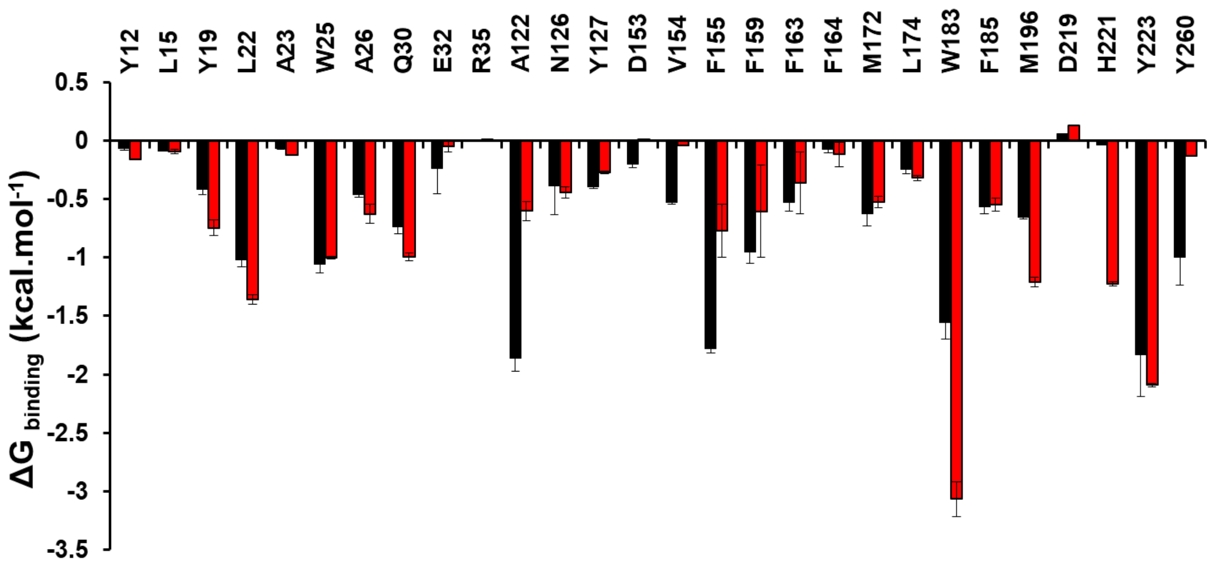

Figure S11. Residues contributing to the effective binding energies of the single methylated cWW over 7 x 200 ns of MD simulation. The black and red bars refer to the per-residue average MMGBSA effective energy values for the single methylated *LL*-cWW **14** and *DD*-cWW **16**, respectively.

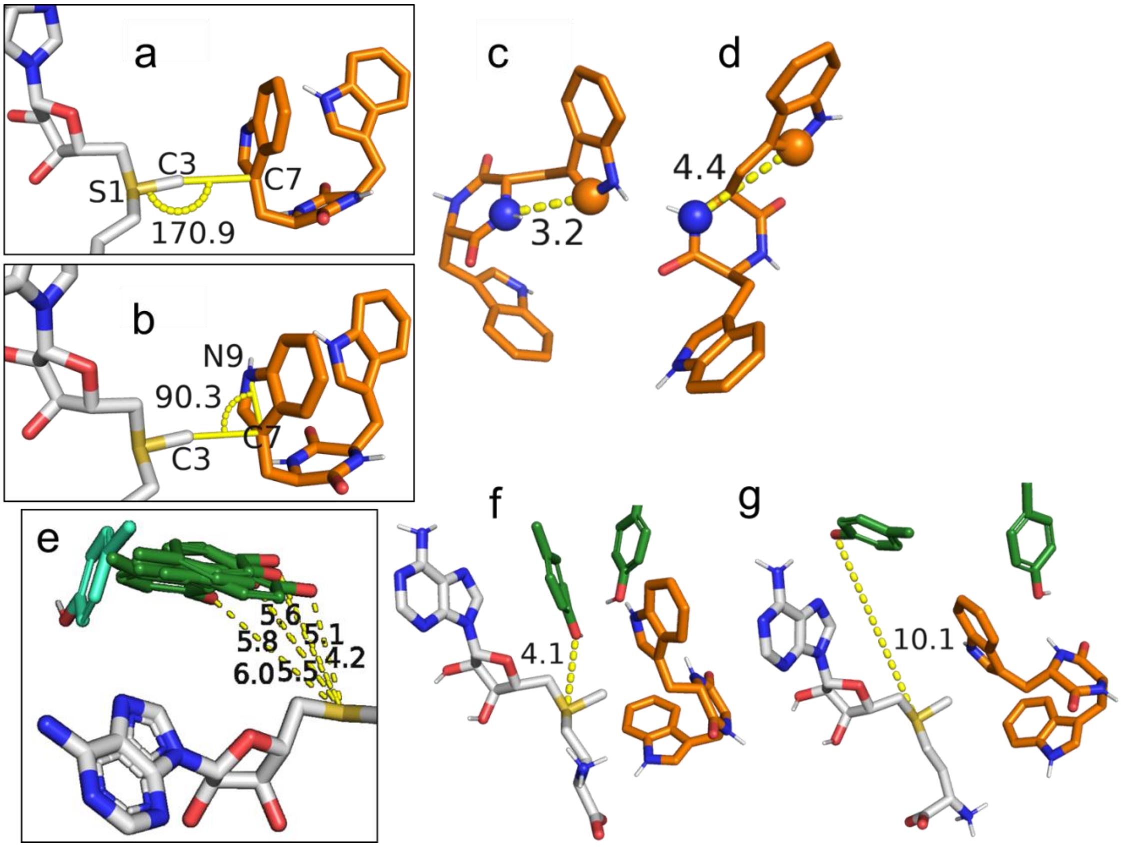

Figure S12. Geometry criteria chosen for the definition of reactive conformations. Examples of *DD*-cWW **7b** conformations (orange) showing acceptable values for the $C3_{SAM}$ -$C7_{indole}$ distance, the $S1_{SAM}$ -$C3_{SAM}$ -$C7_{indole}$ angle (a) and the $C3_{SAM}$ -$C7_{indole}$ -$N9_{indole}$ angle (b). Examples of compatible (c) and incompatible (d) distances (dashed lines) between the two atoms involved in pyrroloindole cyclization in two conformations of *LL*-cWW **7a**. (e) Catalytic tyrosines (green) equivalent to Y127 in the crystal structures of five methyltransferases sharing a similar fold (PDB IDs 7ZKH, 1WZN, 1Y8C, 3D2L, 3BX0). The cyan conformer, isolated from a single MD trajectory performed in this study, shows an orientation plausibly incompatible with catalysis. (f) Orientation of the Y127 side chain suitable for catalysis. (g) Opposite scenario where the Y127 side chain points away from the cofactor (grey) and is unlikely to contribute to catalysis. For a and b, the legend is identical to Figure 15. The distances between the Y127 hydroxyl group and the sulfur atom of the SAM cofactor are shown by dashed lines with their corresponding value.

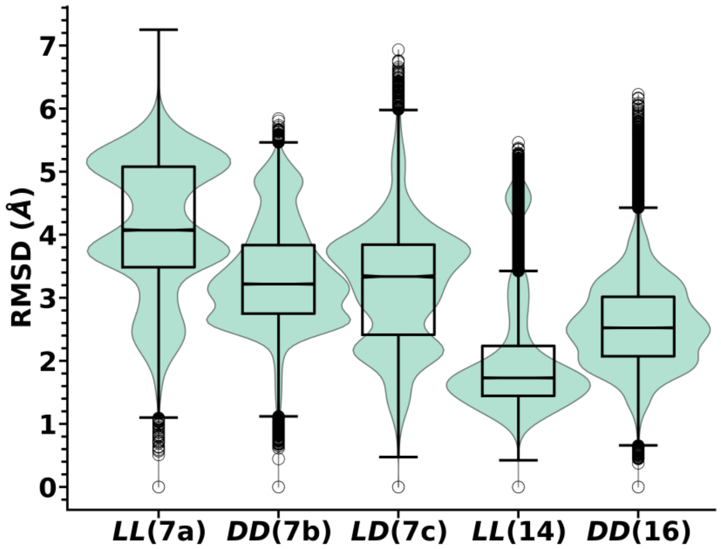

Figure S13. Distribution of the cWW RMSD (heavy atoms) over 7 replicas of 200 ns of MD simulation. The central boxplot line shows the median value. *LL* (**7a**), *DD* (**7b**), *LD* (**7c**) respectively refer to the unmethylated *LL*-cWW (**7a**), *DD*-cWW (**7b**), and *LD*-cWW (**7c**). *LL* (**14**) and *DD* (**16**) respectively refer to the single methylated *LL*-cWW (**14**) and *DD*-cWW (**16**).

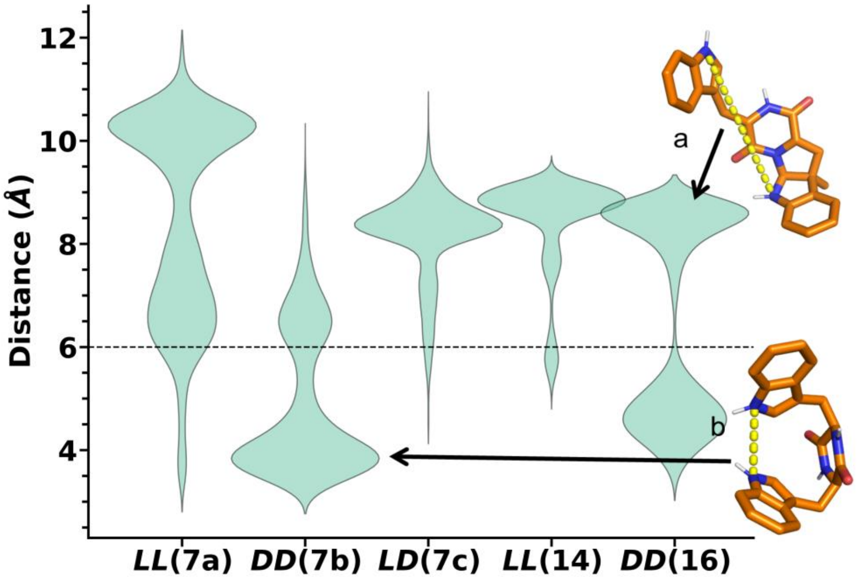

Figure S14. Distribution of the distance between the indole rings nitrogen atoms of the cWW over 7 replicas of 200 ns of MD simulation. *LL* (**7a**), *DD* (**7b**), *LD* (**7c**) respectively refer to the unmethylated *LL*-cWW **7a**, *DD*-cWW **7b**, and *LD*-cWW **7c**. *LL* (**14**) and *DD* (**16**) respectively refer to the single methylated *LL*-cWW **14** and *DD*-cWW **16**. A "stretched" conformation of the single methylated *LL*-cWW (a) and a "boat-like" conformation of the unmethylated *DD*-cWW substrate (b) are shown in comparison. The dashed line illustrates the distance measured and the black arrows points to the corresponding distance values. The black line at 6 Å is a crude distance threshold to separate these two classes of conformations.

Figures S15-18: Lysate activities

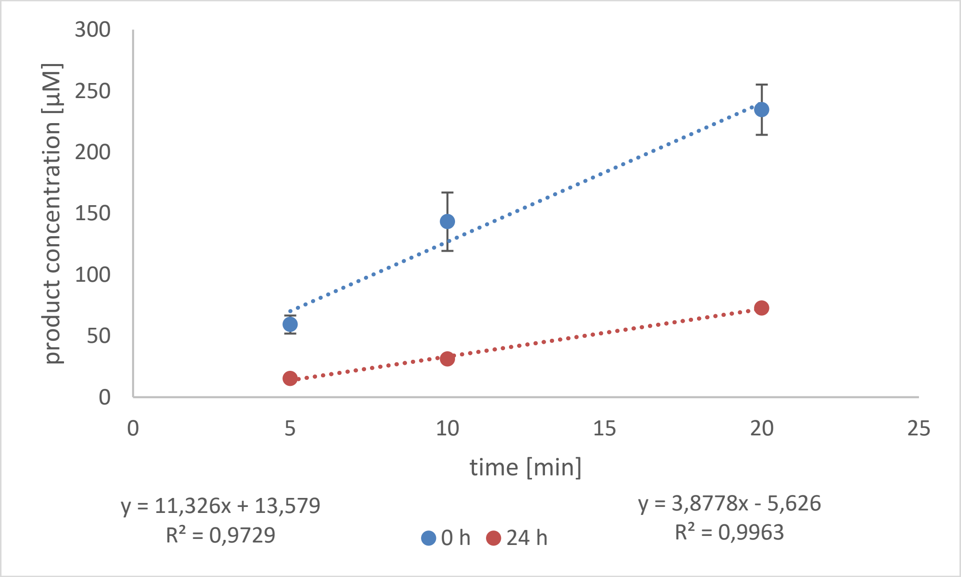

Figure S15. Lysate activity of StspM1 after 0 h and 24 h incubation at 40 °C and 700 rpm. Reaction was performed under *in vitro* assay conditions with 40% lysate and an additional 1 mM SAM. Lysate activity (0 h) in triplicates: 28.3 mU/mL; Lysate activity (24 h): 9.7 mU/mL.

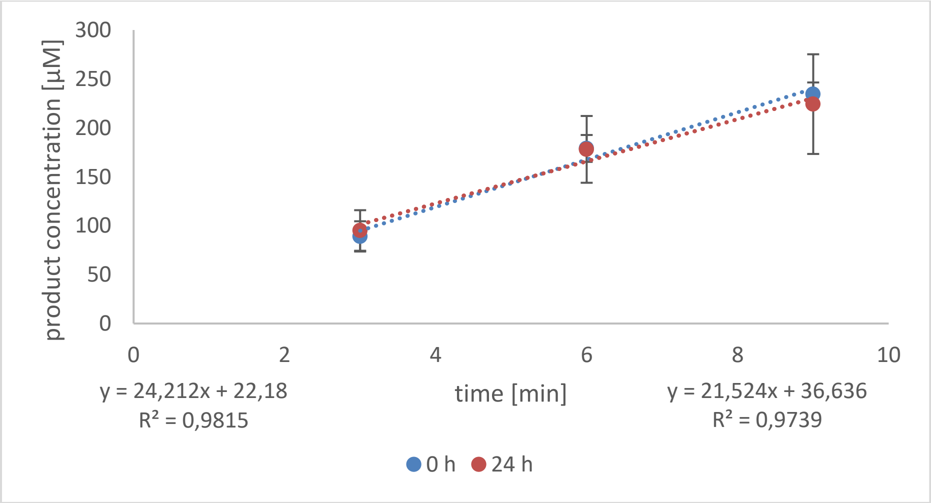

Figure S16. Lysate activity of HMT after 0 h and 24 h incubation at 40 °C and 700 rpm. Reaction was performed under *in vitro* assay conditions with 3% lysate and 5 mM MeI, Lysate activity (0 h) in triplicates: 0.81 U/mL; Lysate activity (24 h): 0.72 U/mL.

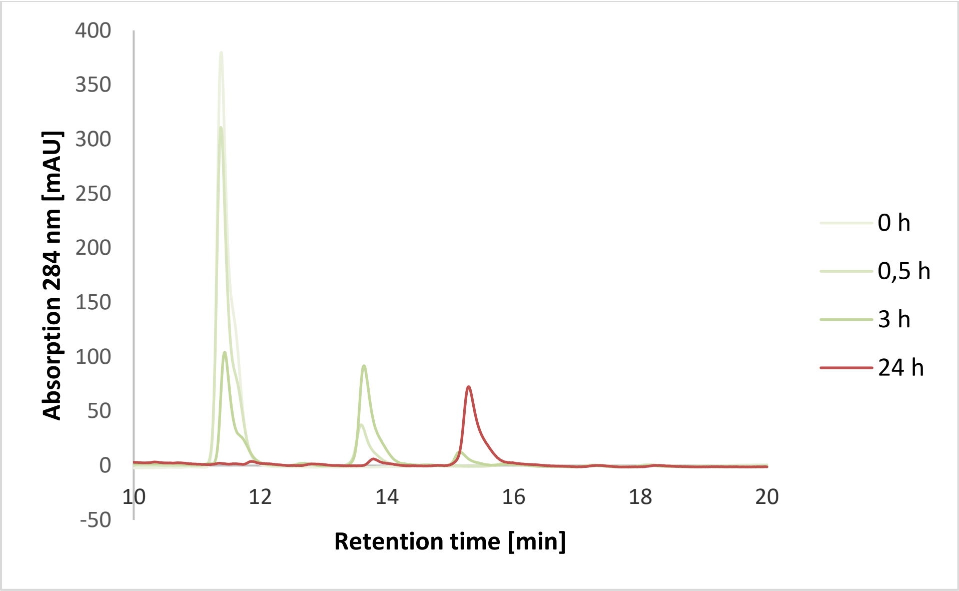

Figure S17. HPLC chromatogram of the conversion of *LL*-cWW **7a** at different time points. Reaction was carried out under in *vitro assay* conditions with 80% StspM1 lysate and 20% HMT Lysate. Retention times: 11.45 min (substrate **7a**), 13.65 min (single methylated product **14**), 15.16 min (double methylated product **15**).

Time [h]	Substrate	Single methylated	Double methylated
0,5	85%	16%	-
1	41%	36%	22%
3	26%	41%	33%
24	-	1%	99%
Negative control 1	100%	-	-
Negative control 2	100%	-	-

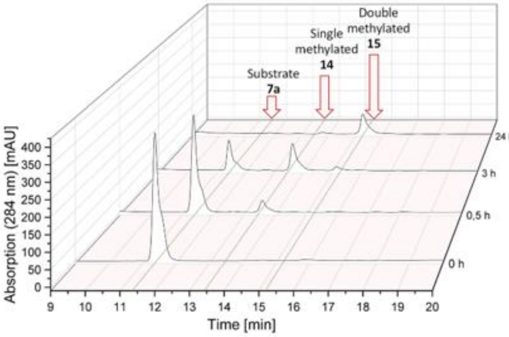

Figure S18. Reaction of *LL*-cWW **7a** under *in vitro* assay conditions with 80% StspM1 lysate and 20% HMT Lysate. Retention times: 11.45 min (substrate), 13.65 min (single methylated product), 15.16 min (double methylated product). Negative control 1 contained 80 Vol% StspM1 lysate and 20 Vol% lysate with an empty vector. Negative control 2 contained 80 Vol% lysate with an empty vector and and 20 Vol% HMT lysate.

Figure and equation 19: Design of experiment

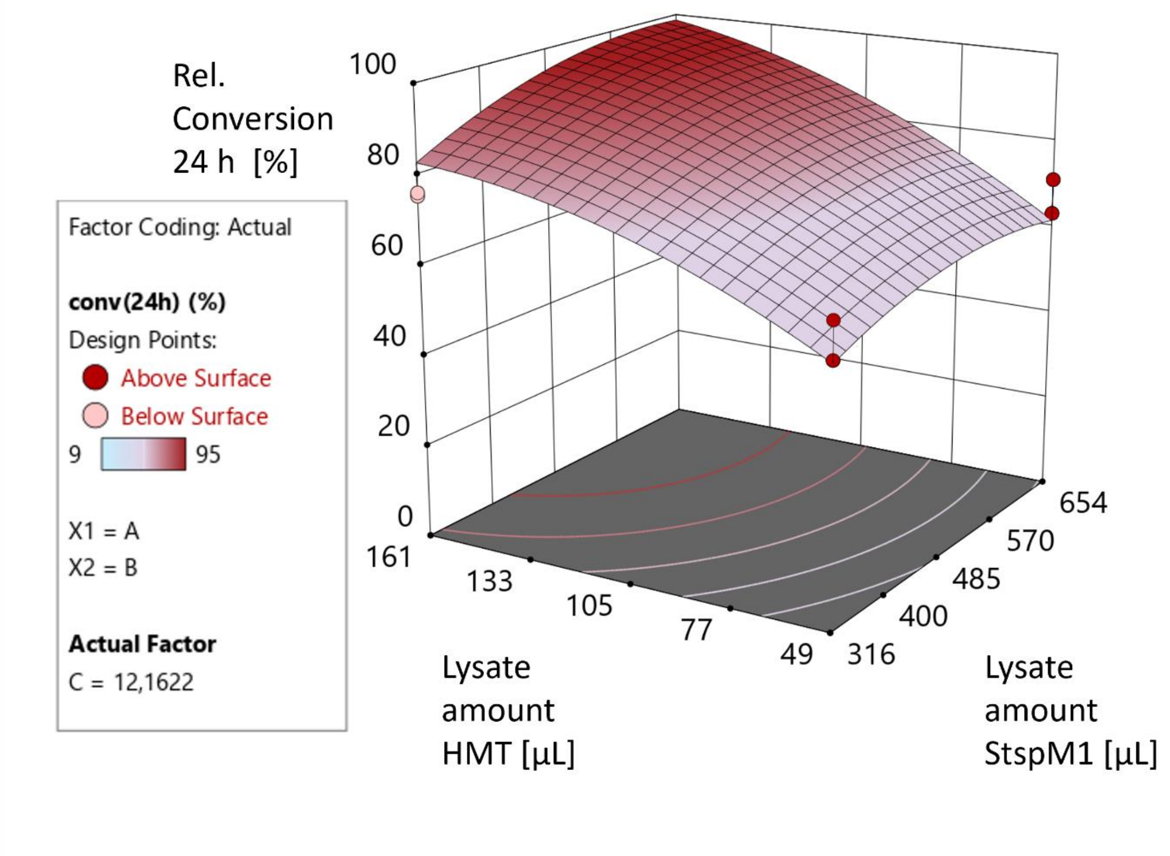

Figure S19. Result of the design of experiment approach. The conversion after 24 h of the *LL*-cWW **7a** was modeled by the hypersurface in the 3D diagram. The hypersurface is described by the equation: conversion= -24,99 % + 0,064291 $\%\mu L^{-1}\times$ V(StspM1) + 0.584892 $\%\mu L^{-1}\times$ V(HMT) + 4,63827 $\%\mu M^{-1}$ c(MeI) + 0,000235 $\%\mu L^{-1}\ \mu L^{-1}$ V(StspM1)×V(HMT) + 0,008614 $\%\mu M^{-1}\ \mu L^{-1}\times$c(MeI)×V(StspM1) – 0,005232 $\%\mu M^{-1}\ \mu L^{-1}\times$c(MeI)×V(HMT) – 0,000166 $\%\mu L^{-1}\times$V(StspM1)^2 – 0,00168 $\%\mu L^{-1}\times$V(HMT)^2 – 0,253316 $\%\mu M^{-1}$c(MeI)^2.

Figures S20+21: Activity of immobilized enzymes

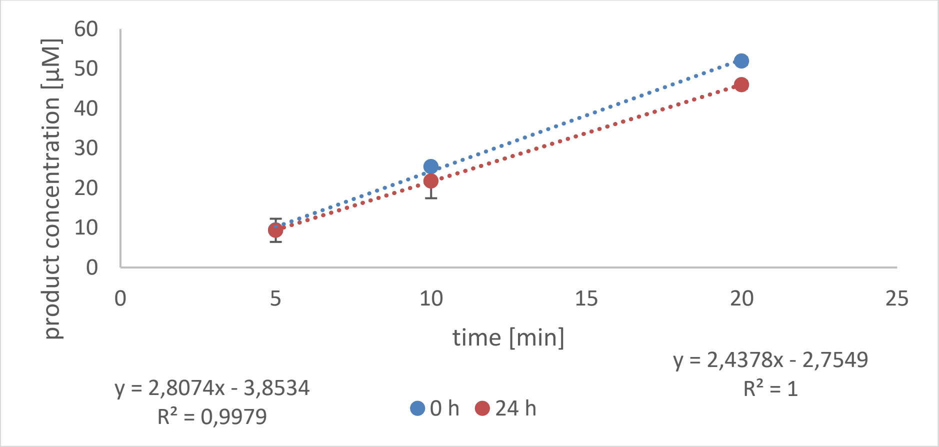

Figure S20. Activity of StspM1 (immobilized from lysate) after 0 h and 24 h incubation at 40 °C and 700 rpm. Reaction was performed under *in vitro* assay conditions with 40% immobilized lysate and an additional 1 mM SAM in triplicate. Beads activity (0 h): 7.0 mU/mL; beads activity (24 h): 6.1 mU/mL.

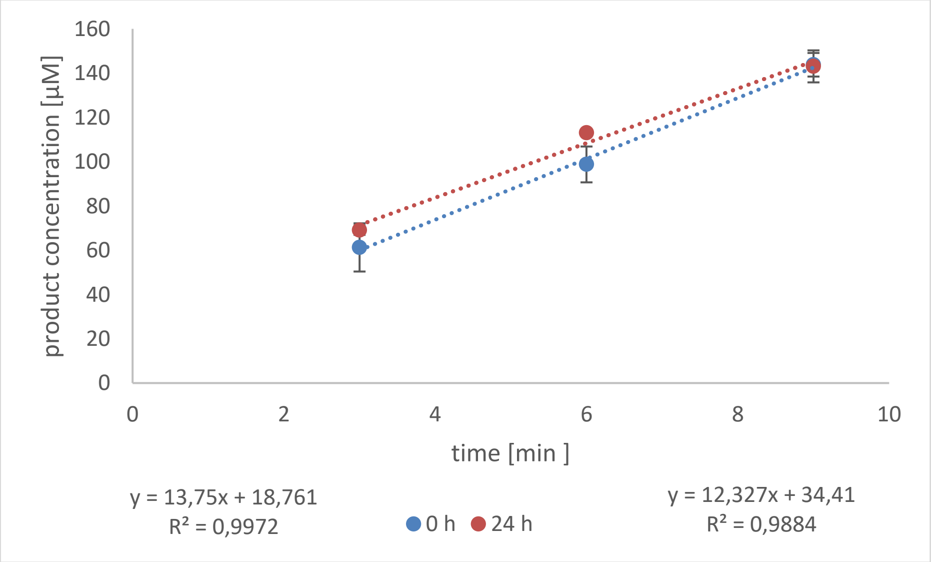

Figure S21. Activity of HMT (immobilized from lysate) after 0 h and 24 h incubation at 40 °C and 700 rpm. Reaction was performed under *in vitro* assay conditions with 3% immobilized lysate and additional 5 mM MeI. Beads activity (0 h) in triplicate: 0.46 U/mL; beads activity (24 h): 0.41 U/mL.

Figure S22: SAH equivalents

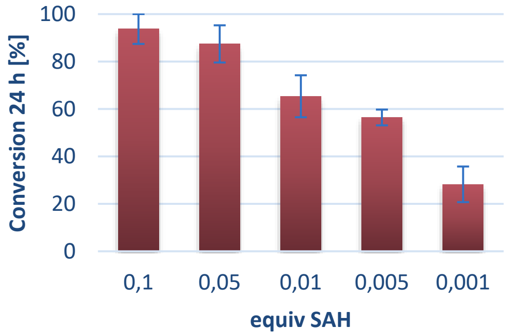

Figure S22. Conversion after 24 h of the methylation reaction of *LL*-cWW **7a** under *in vitro* assay conditions with immobilized enzymes and different concentrations of SAH.

Figures S23+24: Preparative enzymatic methylation

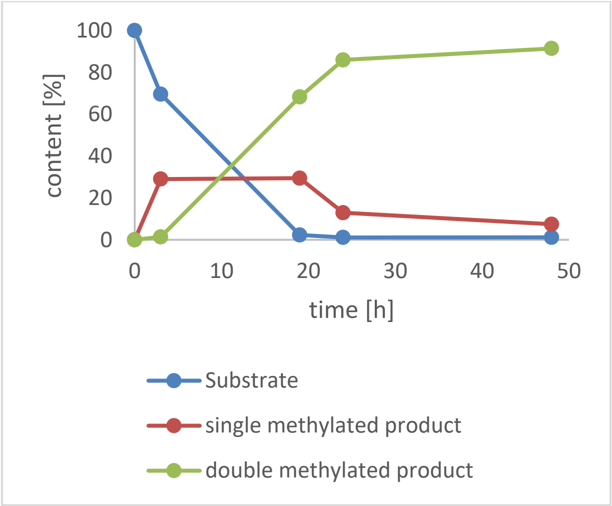

Figure S23. Conversion of *LL*-cWW **7a** at different time points (monitored via HPLC). Reactions were carried out under preparative scale reaction conditions (immobilized enzymes).

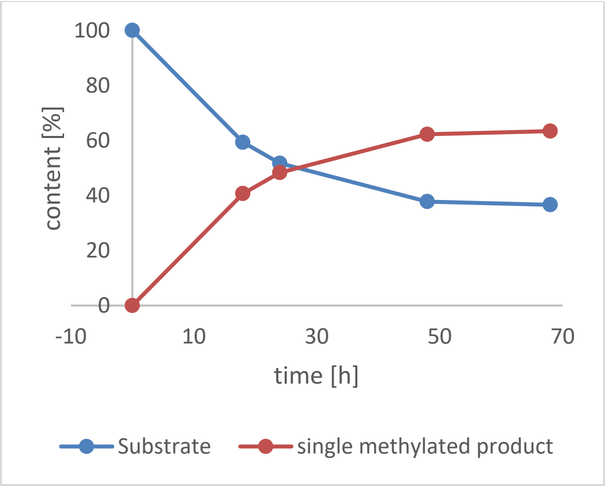

Figure S24. Conversion of *DD*-cWW **7b** at different time points (monitored via HPLC). Reactions were carried out under preparative scale reaction conditions (immobilized enzymes) with an doubled enzyme load.

Figure S25: SDS gel of immobilization process

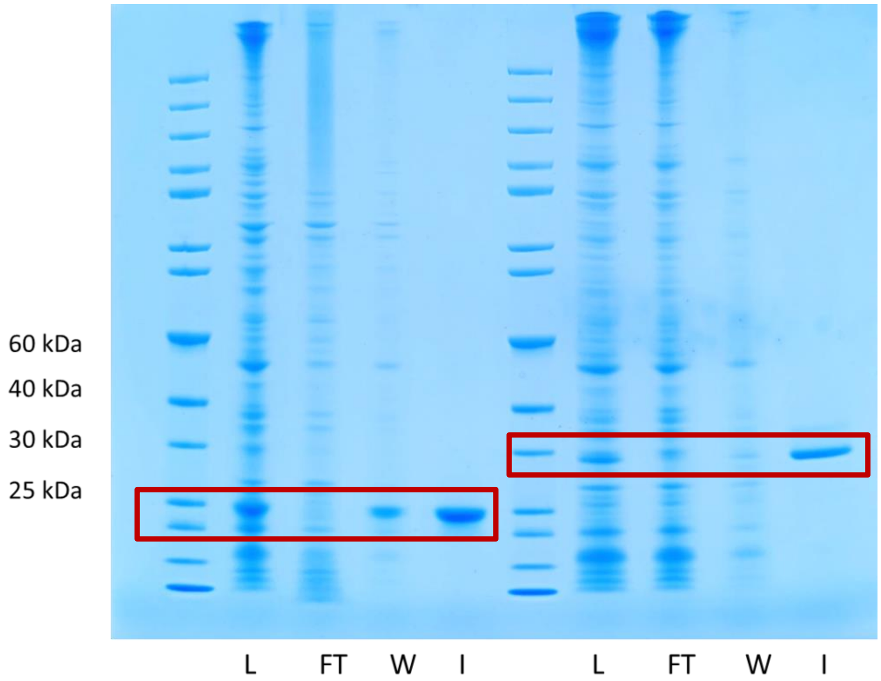

Figure S25. SDS gel of the immobilization of the HMT (24.617 kDa, left) and StspM1 (30.879 kDa, right). As standard, the Page Ruler unstained protein ladder (0.02 – 0.05 µg/µL) has been used. For the lysate and flow through: 2 µL sample have been diluted in 10 µL water before applying to the gel; the other samples were applied undiluted. L = lysate, FT = flow through, W = washing fraction, I = immobilization fraction.

Figure S26: Mutants Y127A and Y127F

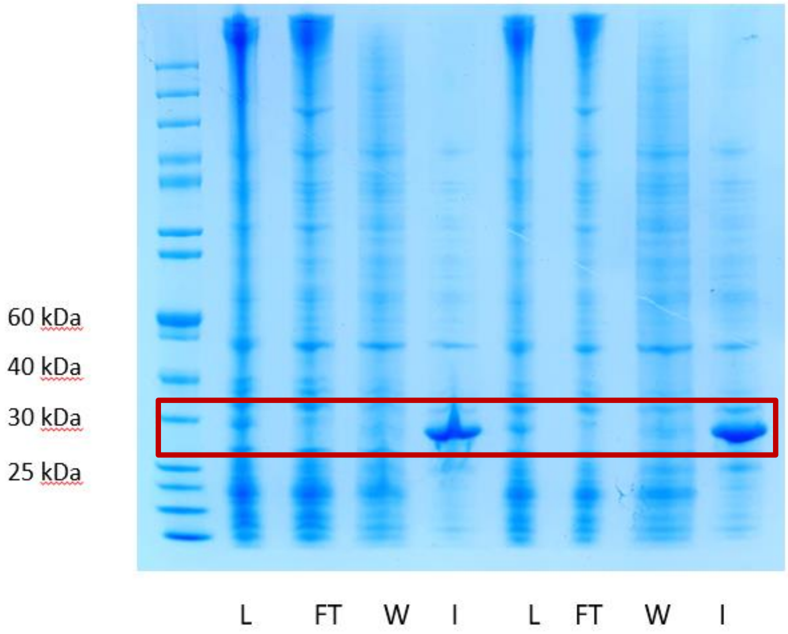

Figure S26. SDS gel of the immobilization of the Y127A (left) and Y127F (right). As standard the Page Ruler unstained protein ladder (0.02 – 0.05 µg/µL) has been used. L = lysate, FT = flow through, W = washing fraction, I = immobilization fraction. The immobilized enzymes were used under *in vitro* assay conditions showing no activity.

Table S3: Conversion under initial conditions

Table S3. Conversion of substrates *LL*-cWW **7a**, *DD*-cWW **7b**, and *LD*-cWW **7c** under initial conditions [47] and with an exchanged buffer. Initial conditions: pH 7.5, 50 mM Tris, 100 mM NaCl, 1 mM SAM, 1 mM *LL*-cWW, 40 µM stspM1, 120 min, 30 °C; optimized conditions: pH 7.5, 50 mM KPi, 1 mM SAM, 1 mM cWW, 40 µM stspM1, 120 min, 40 °C; negative control: pH 7.5, 50 mM KPi, 1 mM SAM, 1 mM cWW, 120 min, 40 °C. Conversions have been analyzed via RP-HPLC.

**Conditions**	**Substrate**	**Remaining substrate (R_t = 11.5 min)**	**single methylated product (Rt_{LL} = 13.9 min, Rt_{DD}= 15.1 min)**	**double methylated product (Rt_{LL} = 16.0 min)**	**Rel. Conversion**
**Li et al. conditions**	*LL*-cWW	89%	10%	1%	11%
	DD-cWW	95%	5%	-	5%
	LD-cWW	100%	-	-	-
**optimized conditions**	*LL*-cWW	54%	43%	3%	46%
	DD-cWW	80%	20%	-	20%
	LD-cWW	100%	-	-	-
**Negative control**	*LL*-cWW	100%	-	-	-
	DD-cWW	100%	-	-	-

Table S4. Kinetic parameters for the methylation reaction of *LL*-cWW (single methylation), *LL*-cWW (double methylation) and *DD*-cWW (single methylation) determined with the Glo Assay (Promega). Experiments were performed according to the manufacturer's protocol.

	***LL*-cWW 7a (single methylation**	***LL*-cWW 14 (di methylation)**	***DD*-cWW 7b**
**K_m (cWW)**	3.86 ±0,28	32.45 ±2.83	14.75 ±1.90
**K_m (SAM)**	10.39 ±0.79	4.20± 0.46	12.31 ±1.59
**k_{cat} [s^{-1}]**	0.0036	0.0018	0.0011
**v_{max} [µM/min]**	0.32 ±0.01	0.16 ±0.01	0.10 ±0.004
**R^2**	0.986	0.980	0.987

Table S5: SEC of StspM1

Table S5. Results of SEC. V_e = elution volume, V_t=column volume (120 mL), K_{av} = partition coefficient (proportion of pores available to the molecule)

**Protein**	**MW [kDa]**	**V_e [mL]**	**K_{av} =(V_e-V_0)/(V_t-V_0)**
**Blue Dextran : V_0**	2000	47,81	-
**Alpha -Amylase**	200	66,95	0,265
Cytochrome C	12,4	101,94	0,750
ADH	150	70,48	0,314
Carbonic anhydrase	29	86,31	0,533
BSA	66	77,43	0,410
StspM1		78,41	0,424

Table S6-8: DOE conditions and validation

Table S6. Conditions and results of the design of experiment approach. The Design Expert 12 (12.0.7.0) was used to create the conditions. Two additional negative controls (35 and 36) were measured. Reactions were performed under *in vitro* assay conditions.

	**Factor 1**	**Factor 2**	**Factor 3**	**Response**
**Run**	Lysate amount StspM1	Lysate amount HMT	MeI eqiv.	conv (24h)
	µL	µL	equiv	%
**1**	485	105	15	95
**2**	316	161	4	69
**3**	485	105	1	17
**4**	485	200	8	92
**5**	654	49	4	26
**6**	485	105	8	72
**7**	485	105	15	92
**8**	485	105	8	73
**9**	316	49	12	64
**10**	485	200	8	95
**11**	654	161	4	57
**12**	200	105	8	56
**13**	654	161	4	56
**14**	316	161	12	77
**15**	316	49	4	61
**16**	770	105	8	38
**17**	485	105	8	66
**18**	485	105	8	71

**19**	654	49	12	72
**20**	654	49	12	64
**21**	485	105	8	82
**22**	485	105	8	72
**23**	654	161	12	95
**24**	770	105	8	63
**25**	316	161	4	60
**26**	654	49	4	27
**27**	485	10	8	9
**28**	485	10	8	10
**29**	316	49	12	56
**30**	316	161	12	76
**31**	316	49	4	24
**32**	654	161	12	93
**33**	485	105	1	14
**34**	200	105	8	55
**35**	0	0	10	0
**36**	0	0	0	0

Table S7. Calculated conditions for an experiment with a maximal conversion and a minimum amount of StspM1 Lysate. The Design Expert 12 (12.0.7.0) was used to calculate the conditions from the data of the design of experiment.

Factor	Calculated amount
**Lysate amount [µL] STSP_M1 (minimum)**	337,28
**Lysate amount [µL] HMT (in range)**	161,49
**MeI equiv (in range)**	12,16

Table S8. Validation of the predicted conversions with the calculated conditions for an experiment with a maximal conversion and a minimum amount of StspM1 Lysate. The Design Expert 12 (12.0.7.0) was used to calculate the conditions. Reactions were performed under *in vitro* assay conditions.

	**Factor 1**	**Factor 2**	**Factor 3**	**Response**
**Run**	Lysate amount StspM1	Lysate amount HMT	MeI eqiv.	conv (24h)
	µL	µL	equiv	%
**1**	337	161	12	90
**2**	337	161	12	89
**3**	337	161	12	95
**4**	337	161	12	96

NMR spectra

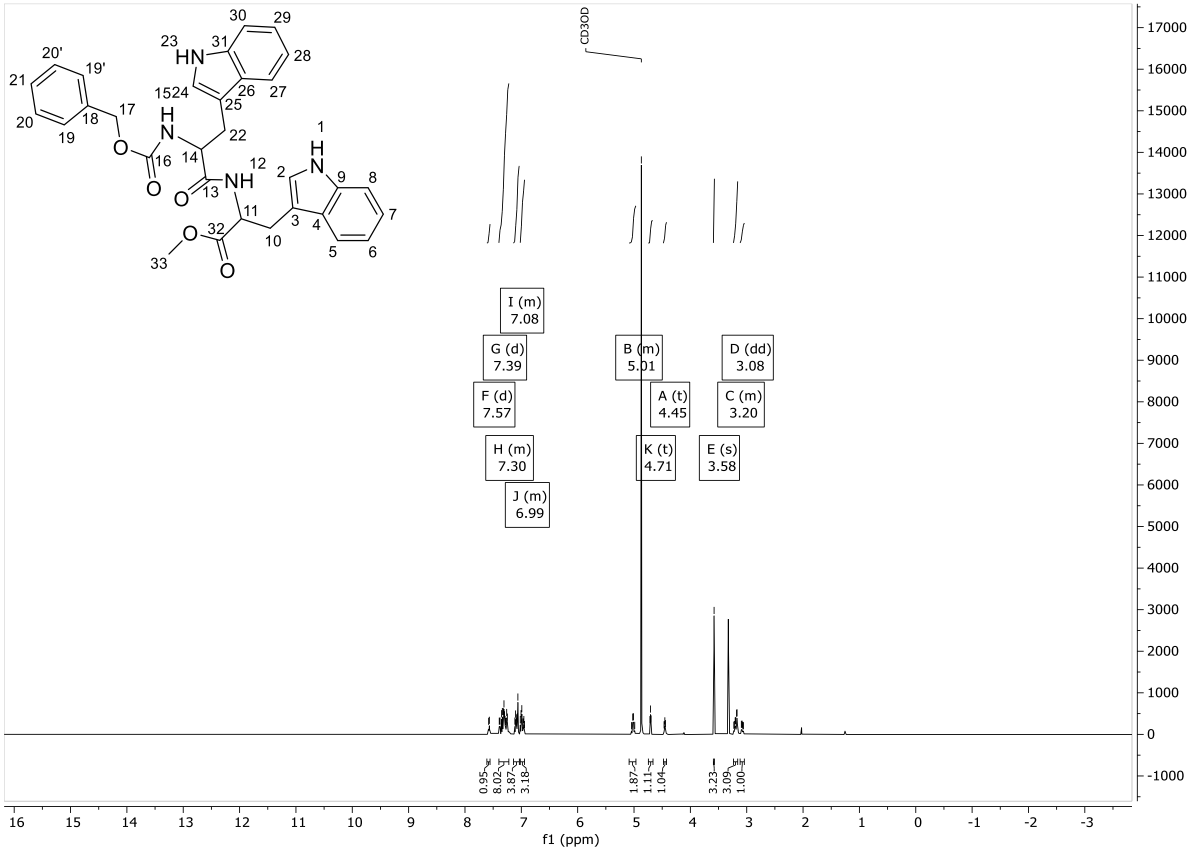

Figure S27. ^{1}H-NMR spectrum of Methyl ((benzyloxy)carbonyl)-*L*-tryptophyl-*L*-tryptophanate (**S3a)** in MeOD (600 MHz).

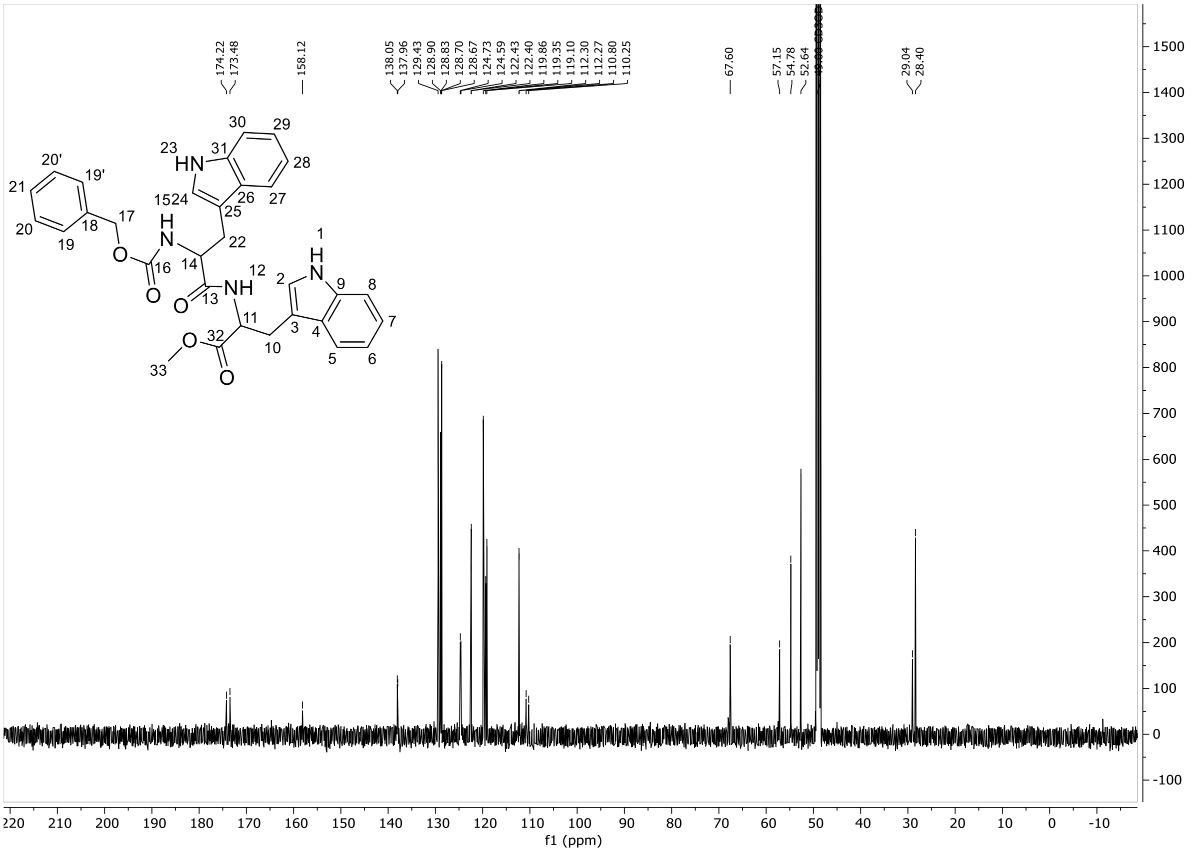

Figure S28. ^{13}C-NMR spectrum of Methyl ((benzyloxy)carbonyl)-*L*-tryptophyl-*L*-tryptophanate (**S3a)** in MeOD (151 MHz).

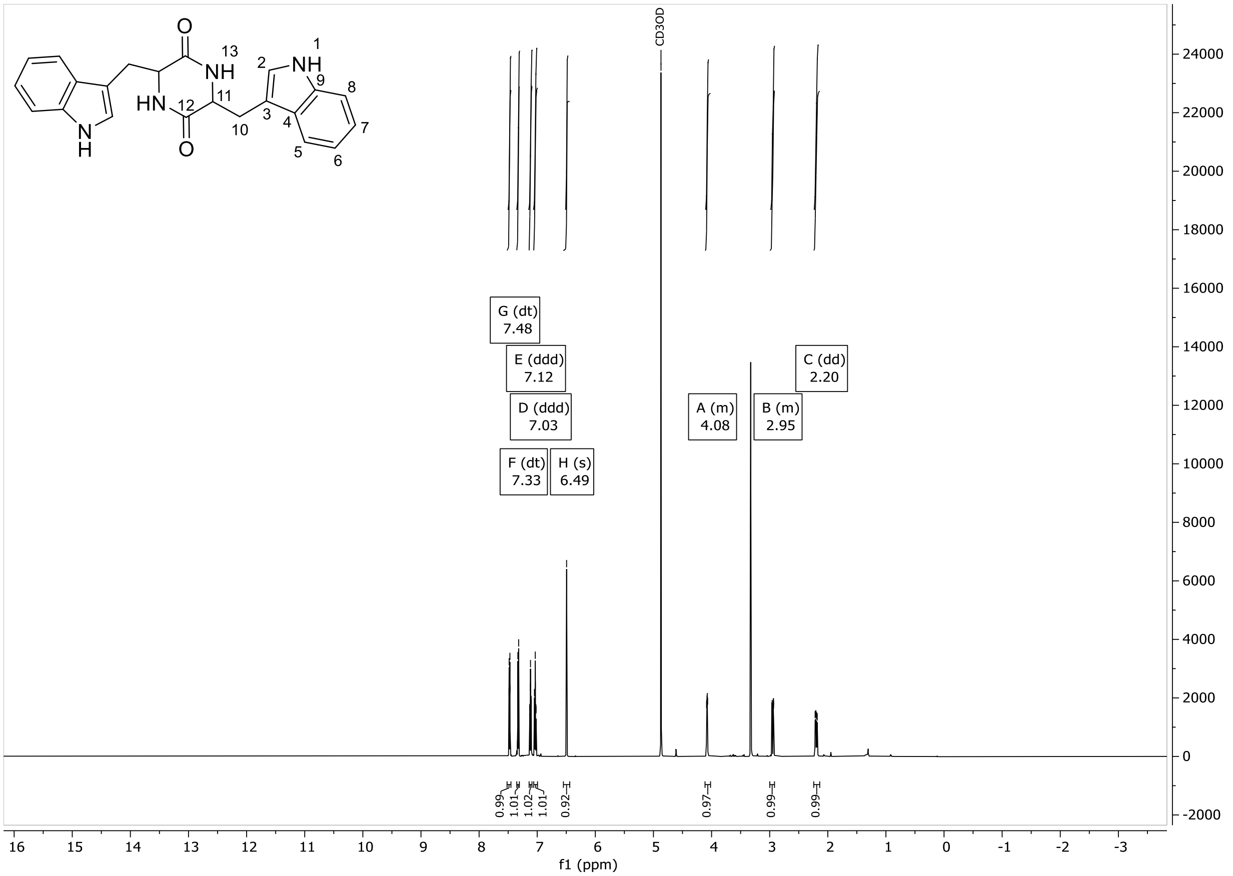

Figure S29. ^{1}H-NMR spectrum of LL-cWW **7a** in MeOD (600 MHz).

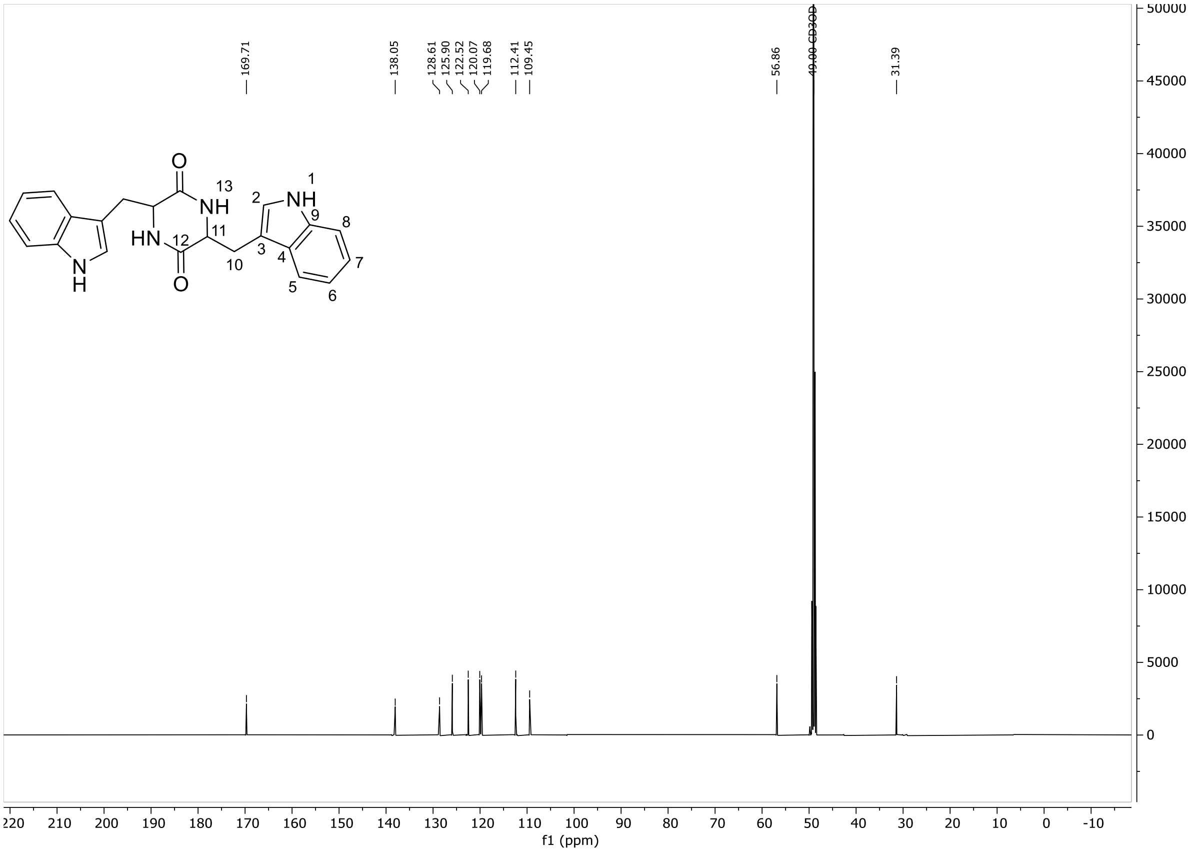

Figure S30. ^{13}C-NMR spectrum of LL-cWW **7a** in MeOD (151 MHz).

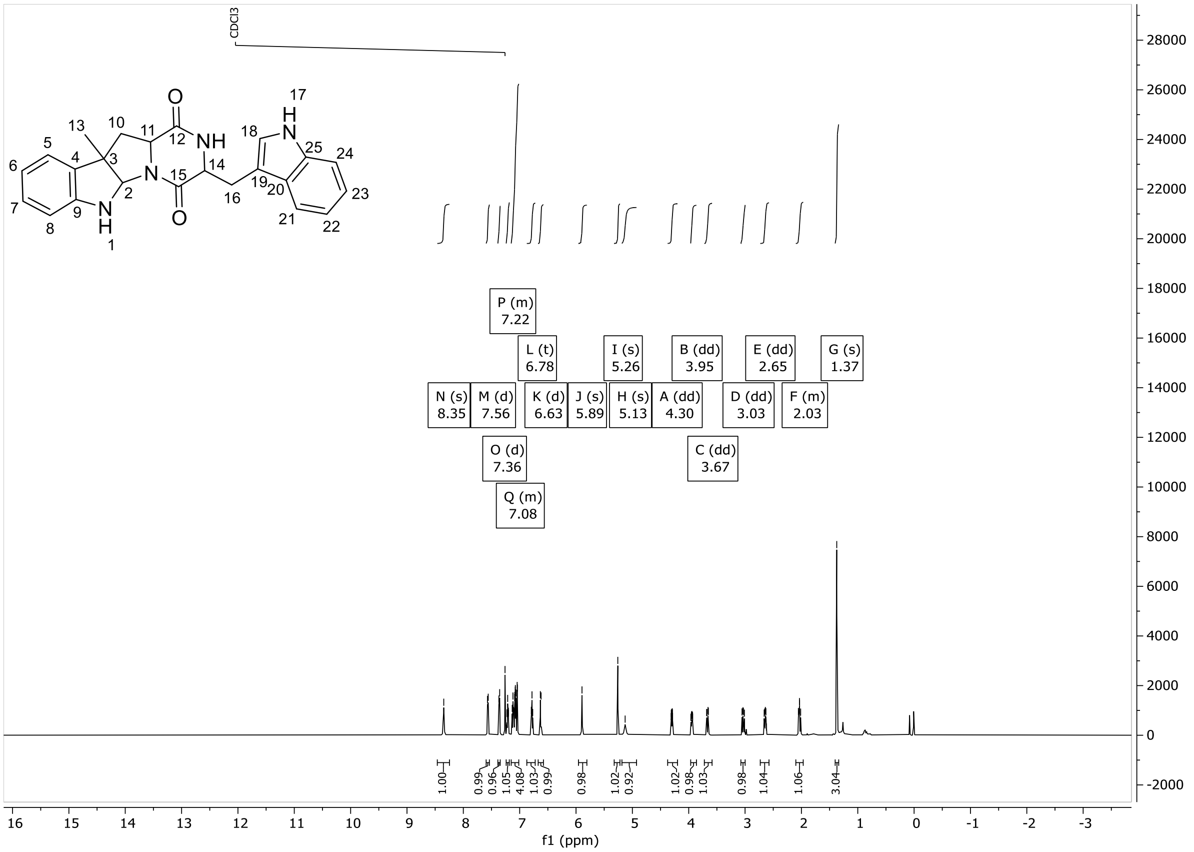

Figure S31. ^{1}H-NMR spectrum of single methylated *LL*-cWW **14** in $CDCl_3$ (600 MHz).

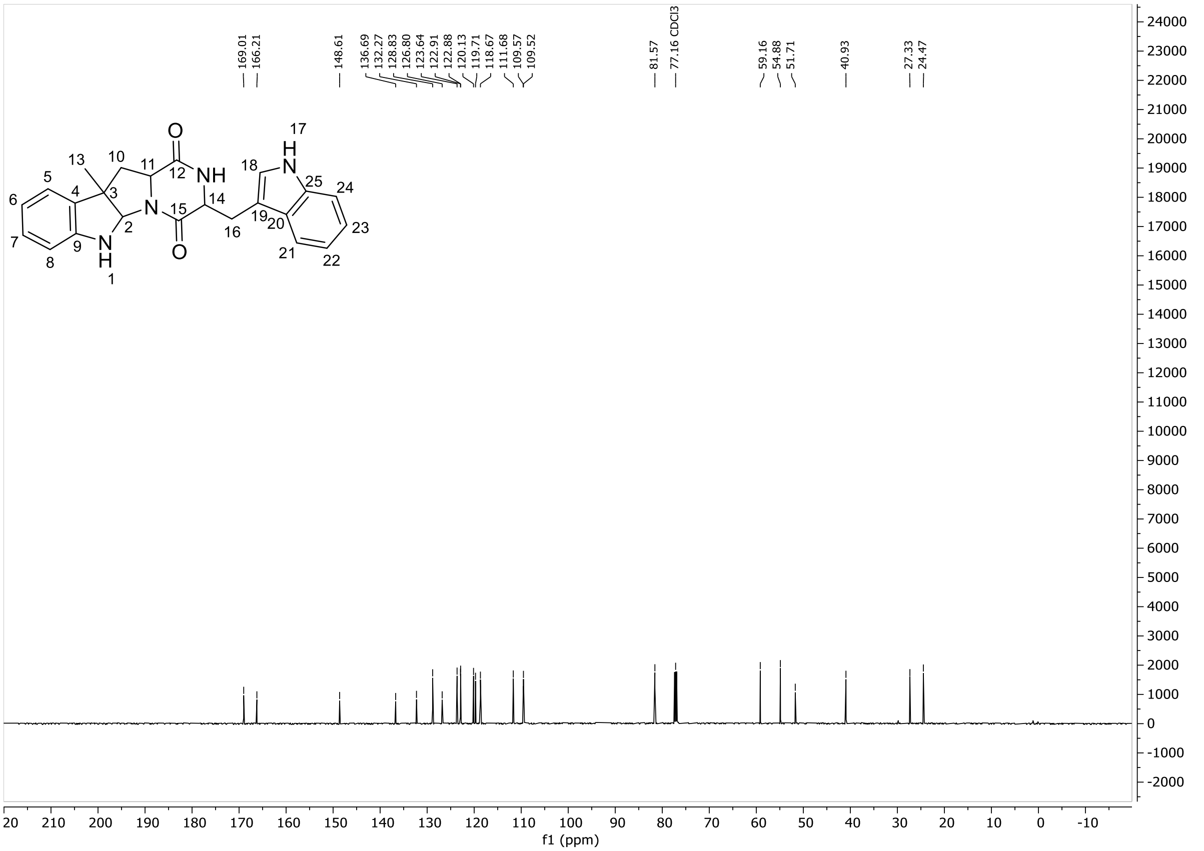

Figure S32. ^{13}C-NMR spectrum of single methylated *LL*-cWW **14** in $CDCl_3$ (151 MHz).

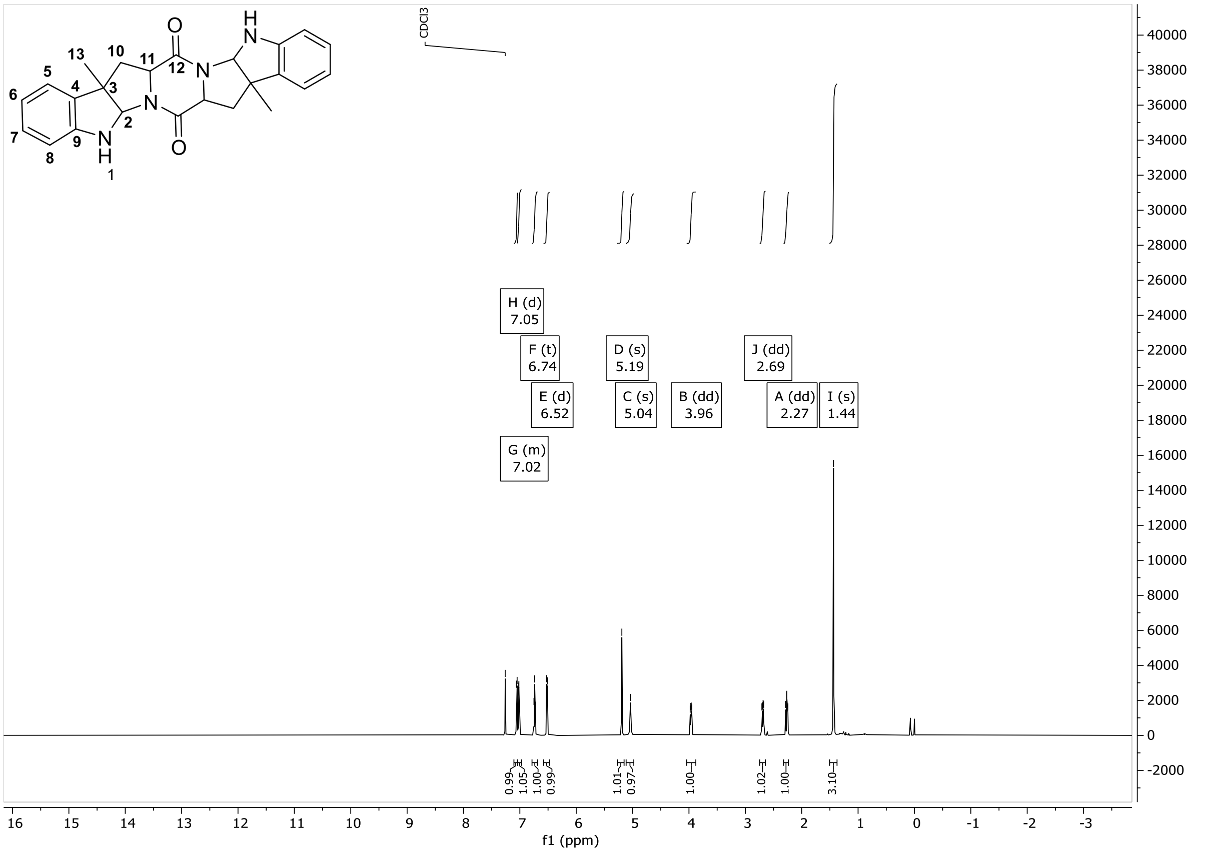

Figure S33. ^{1}H-NMR spectrum of double methylated *LL*-cWW **15** in $CDCl_3$ (600 MHz).

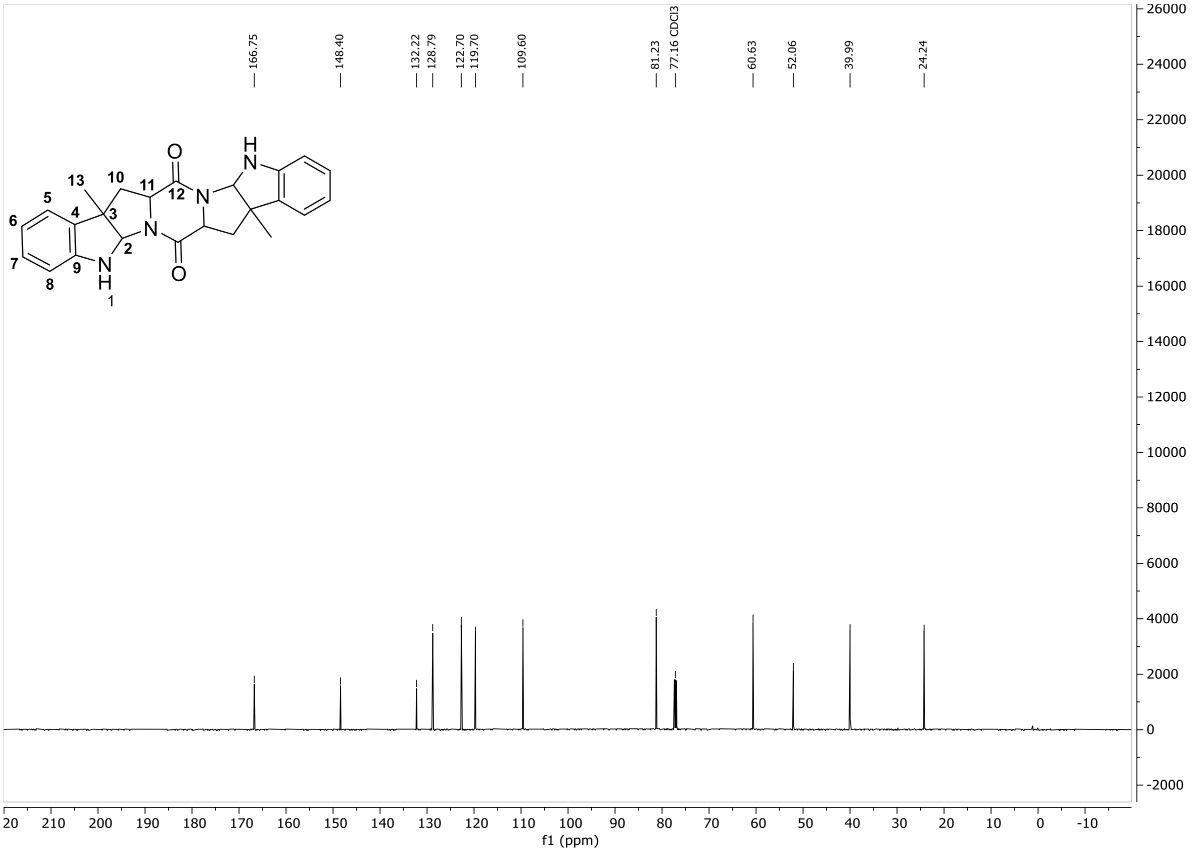

Figure S34. ^{13}C-NMR spectrum of double methylated LL-cWW **15** in $CDCl_3$ (151 MHz).

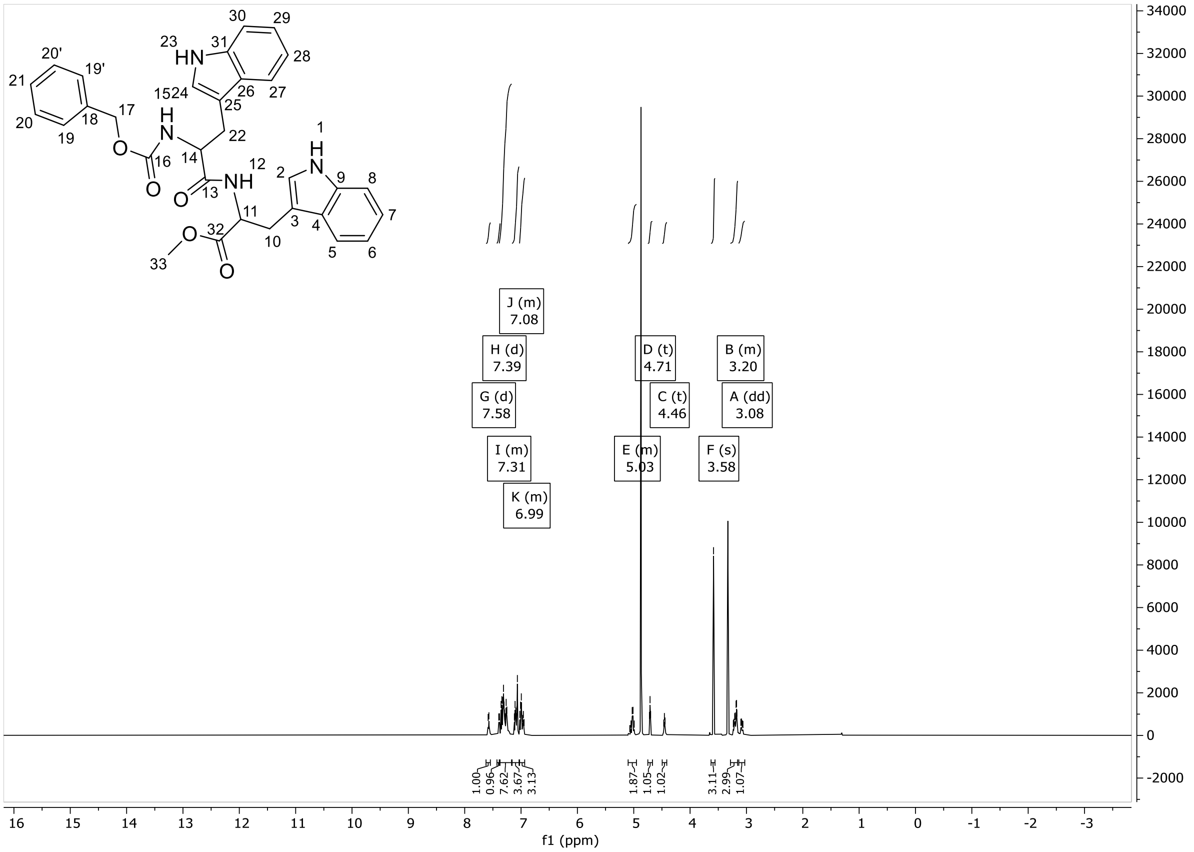

Figure S35. ^{1}H-NMR spectrum of Methyl ((benzyloxy)carbonyl)-*D*-tryptophyl-*D*-tryptophanate **(S3b)** in MeOD (600 MHz).

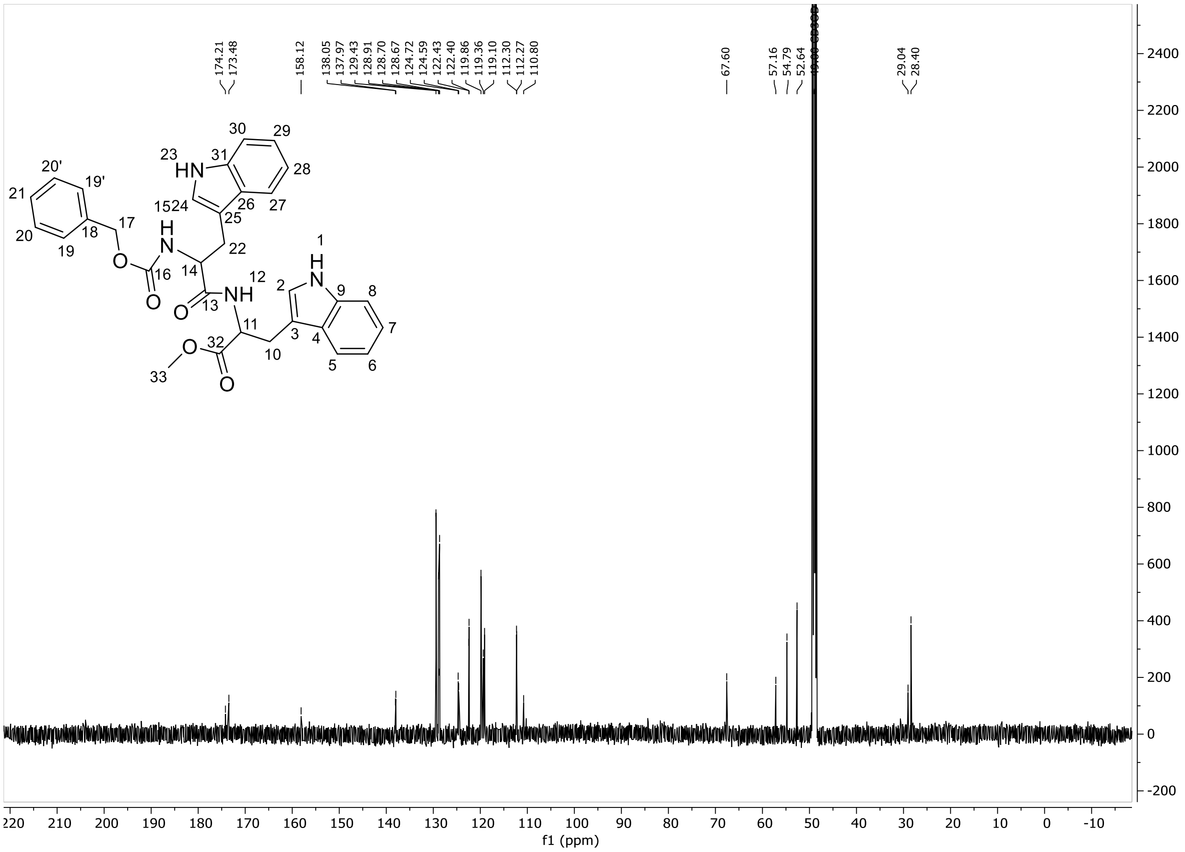

Figure S36. ^{13}C-NMR spectrum of Methyl ((benzyloxy)carbonyl)-*D*-tryptophyl-*D*-tryptophanate **(S3b)** in MeOD (151 MHz).

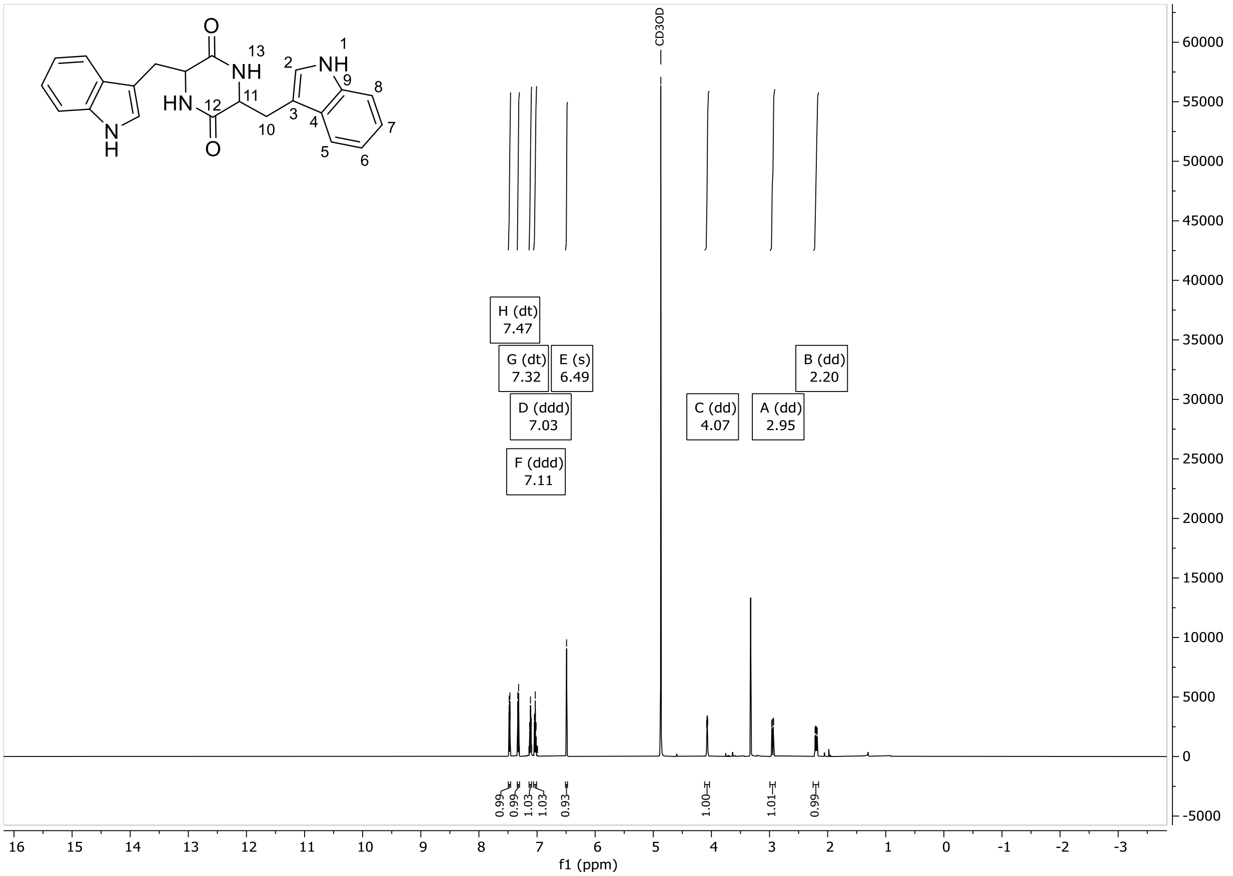

Figure S37. ^{1}H-NMR spectrum of *DD*-cWW **7b** in MeOD (600 MHz).

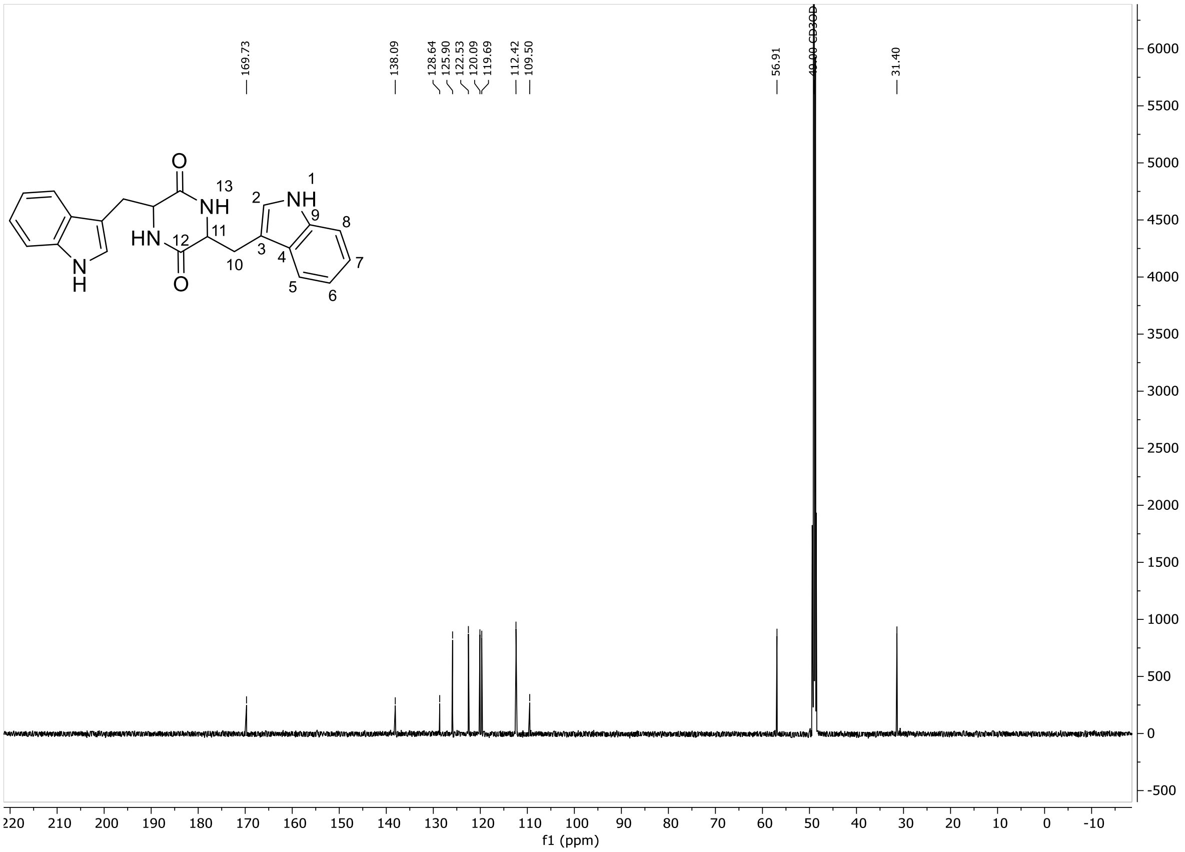

Figure S38. ^{13}C-NMR spectrum of *DD*-cWW **7b** in MeOD (151 MHz).

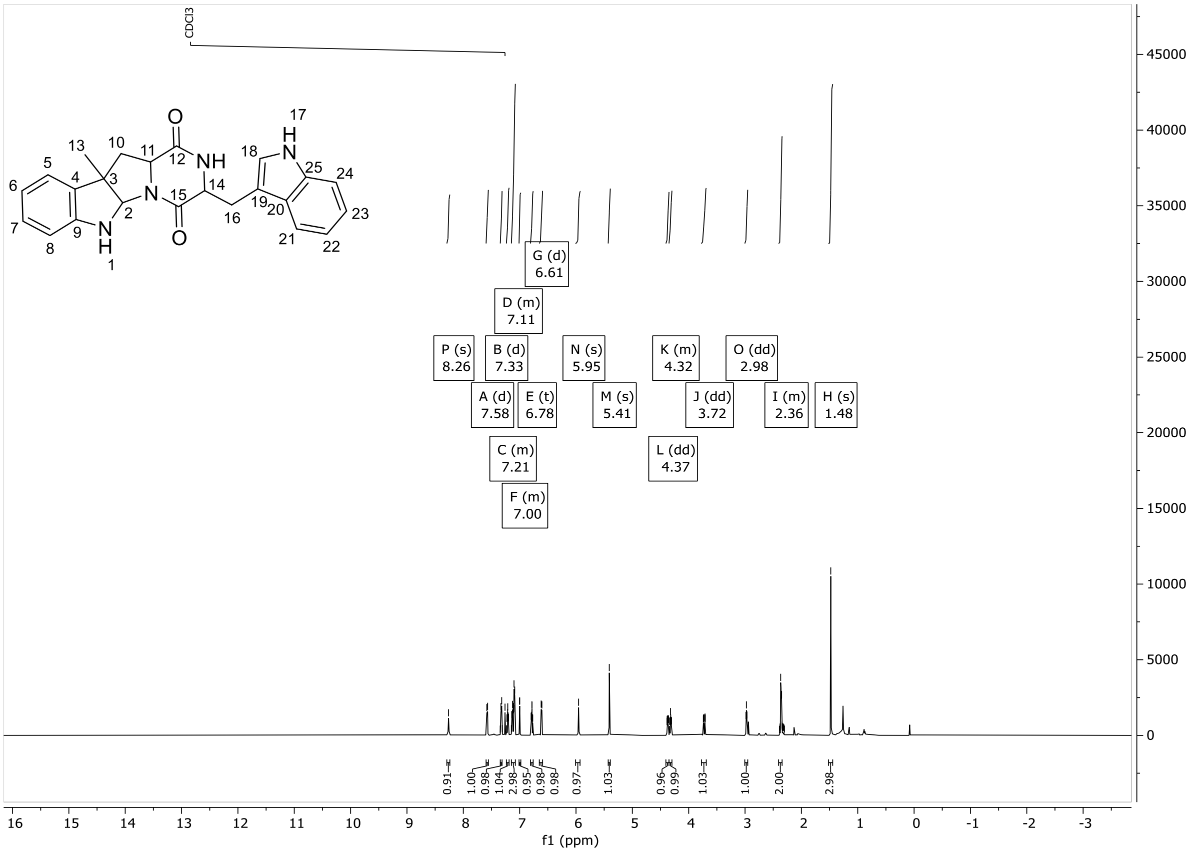

Figure S39. ^{1}H-NMR spectrum of single methylated *DD*-cWW **16** in $CDCl_3$ (600 MHz).

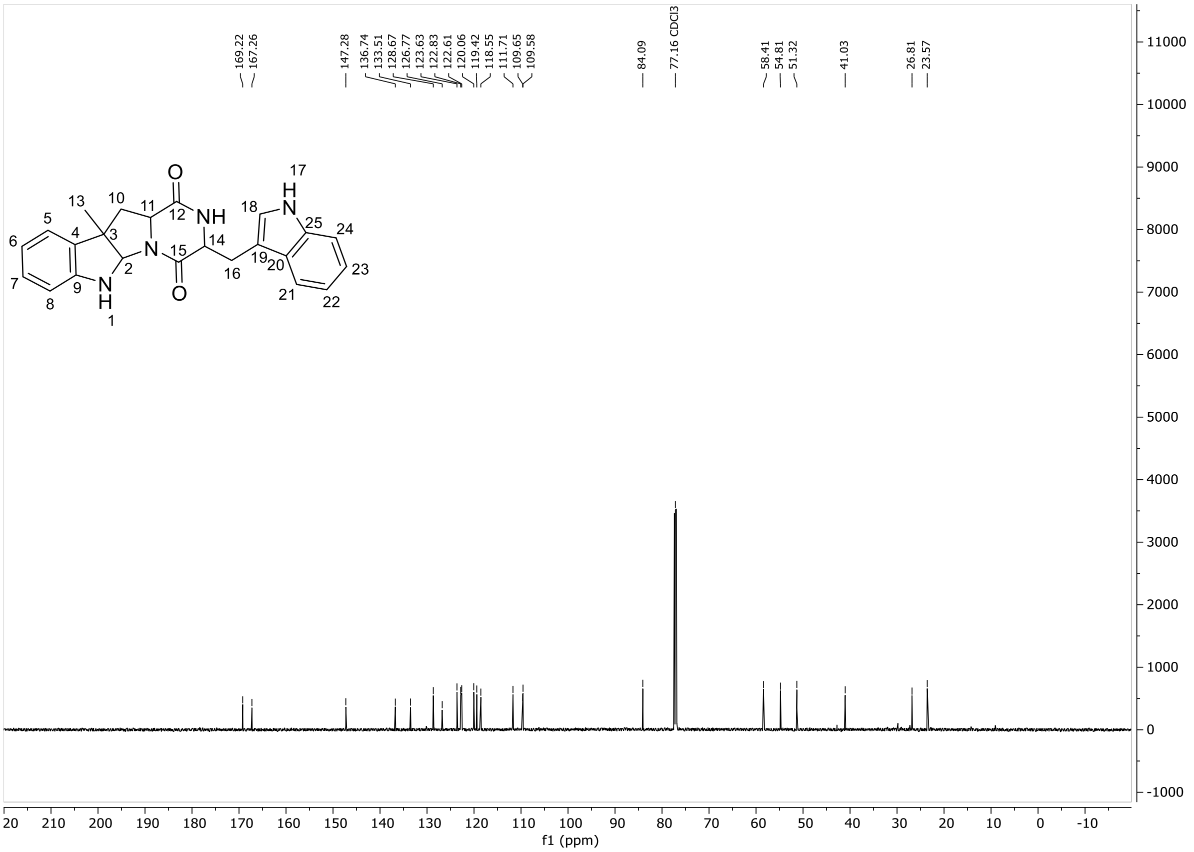

Figure S40. ^{13}C-NMR spectrum of single methylated *DD*-cWW **16** in $CDCl_3$ (151 MHz).

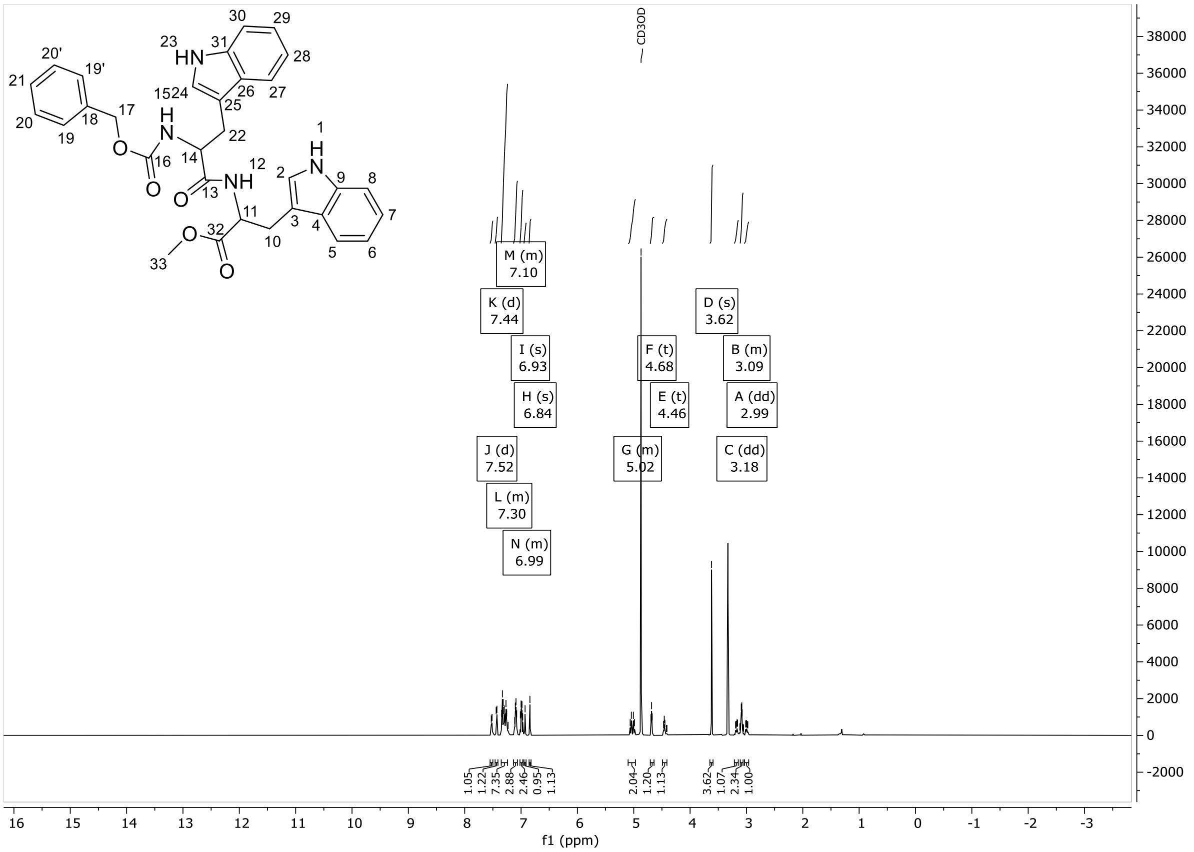

Figure S41. ^{1}H-NMR spectrum of Methyl ((benzyloxy)carbonyl)-*L*-tryptophyl-*D*-tryptophanate **(S3c)** in MeOD (600 MHz).

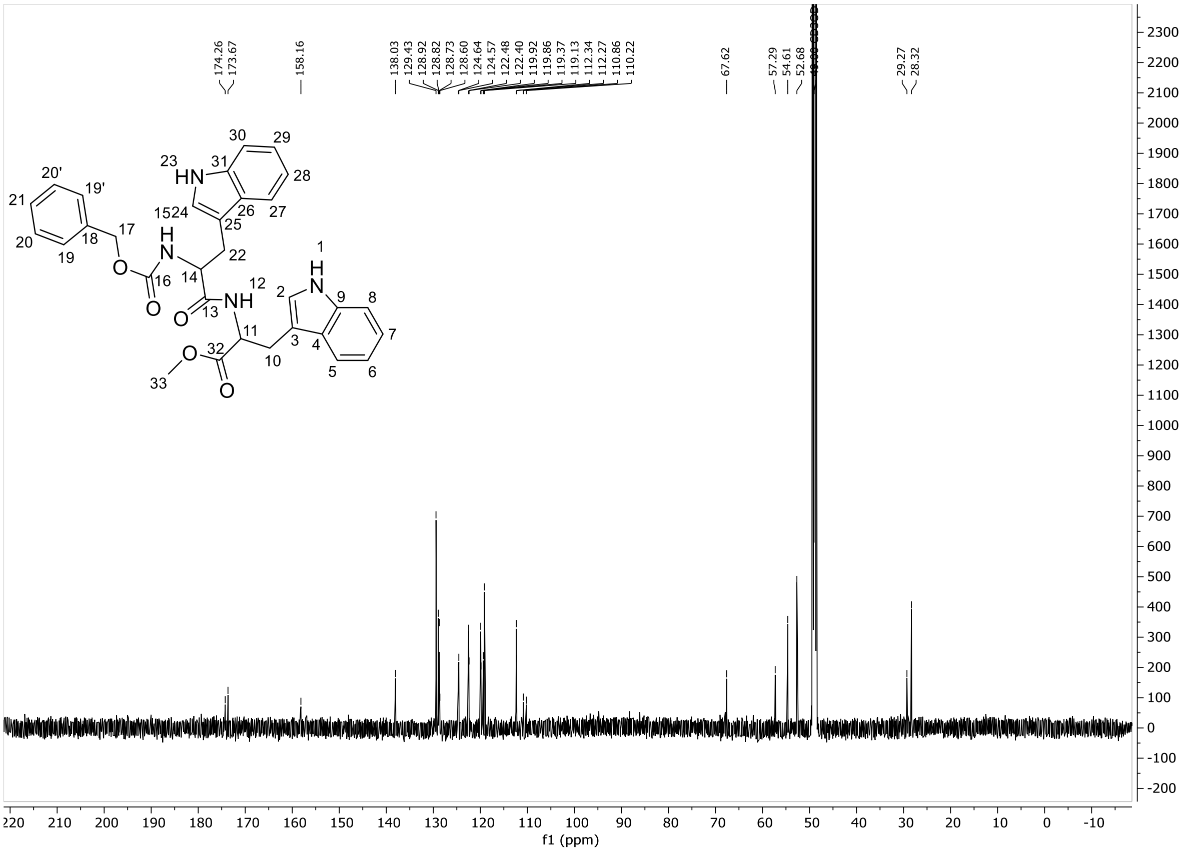

Figure S42. ^{13}C-NMR spectrum of Methyl ((benzyloxy)carbonyl)-*L*-tryptophyl-*D*-tryptophanate **(S3c)** in MeOD (151 MHz).

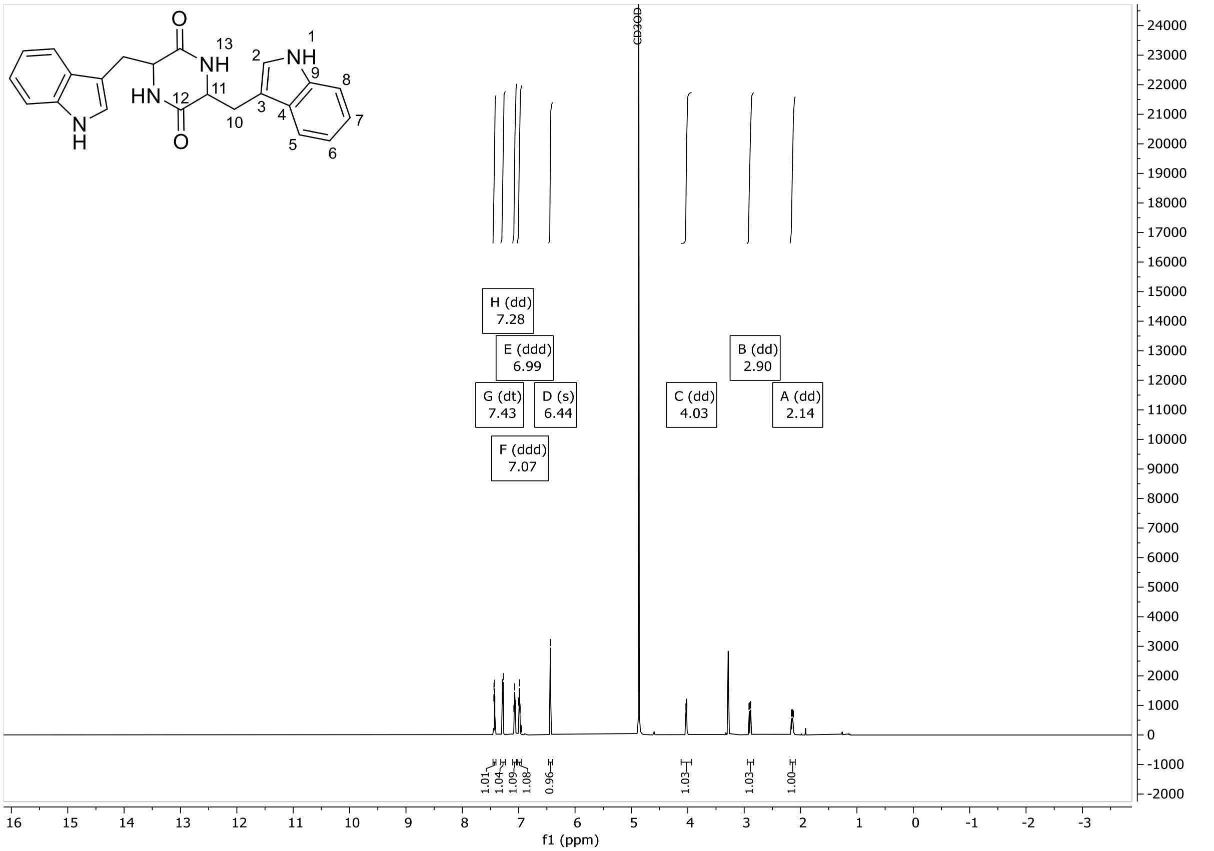

Figure S43. ^{1}H-NMR spectrum of *LD*-cWW **7c** in MeOD (600 MHz).

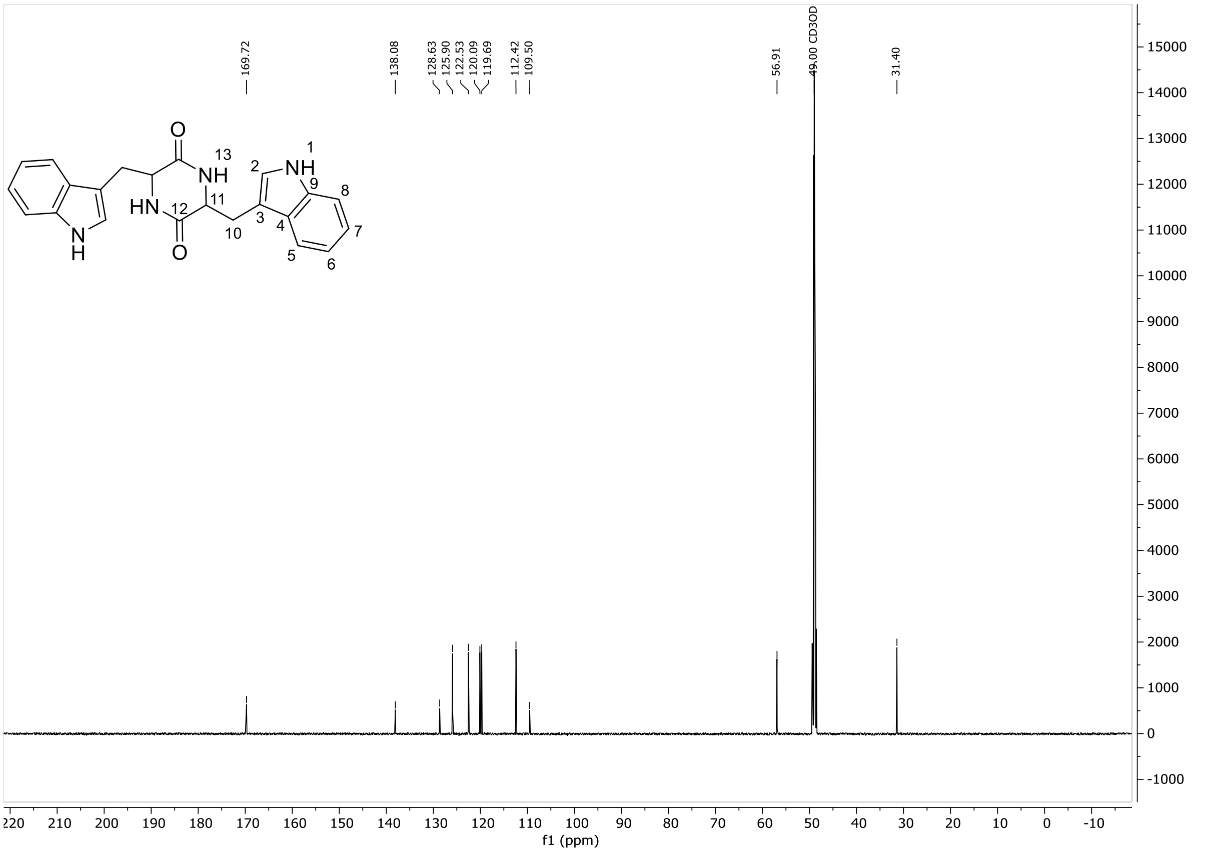

Figure S44. ^{13}C-NMR spectrum of *LD*-cWW **7c** in MeOD (151 MHz).

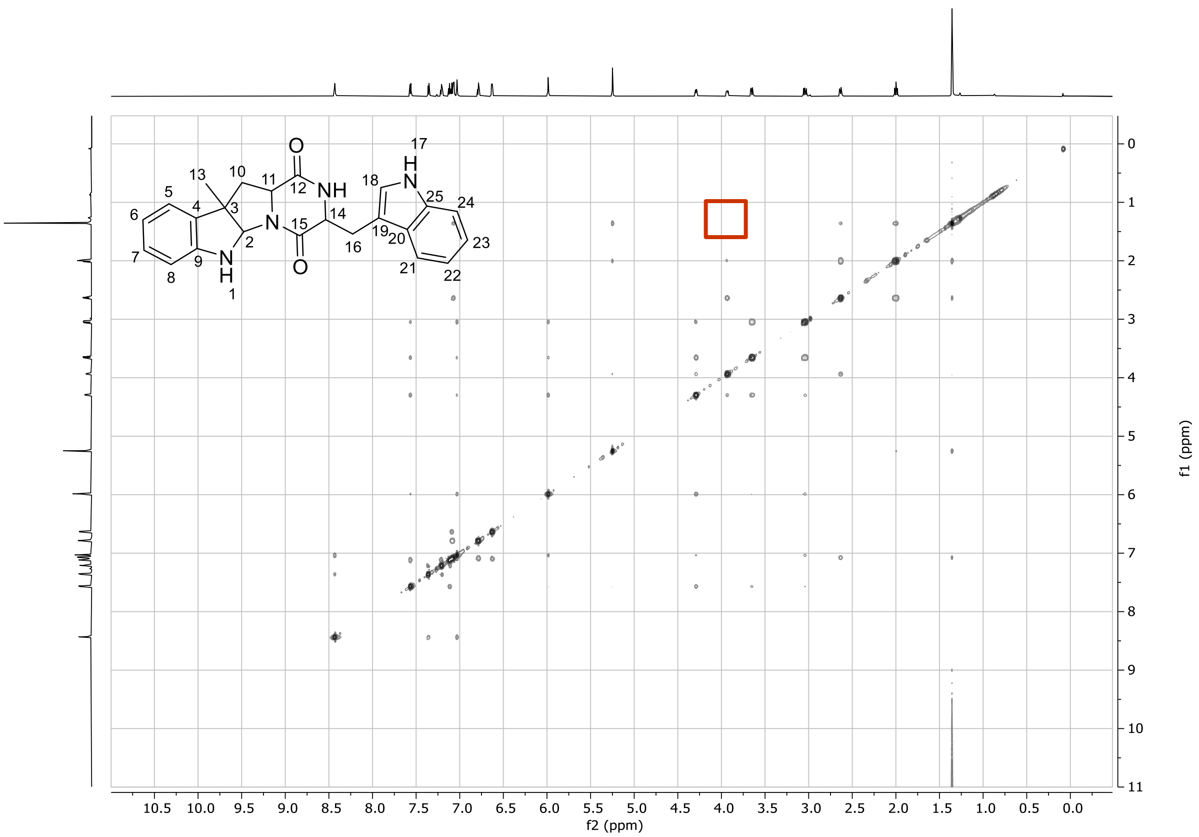

Figure S45. ROESY-spectrum of single methylated *LL*-cWW **14** in $CDCl_3$ (750 MHz).

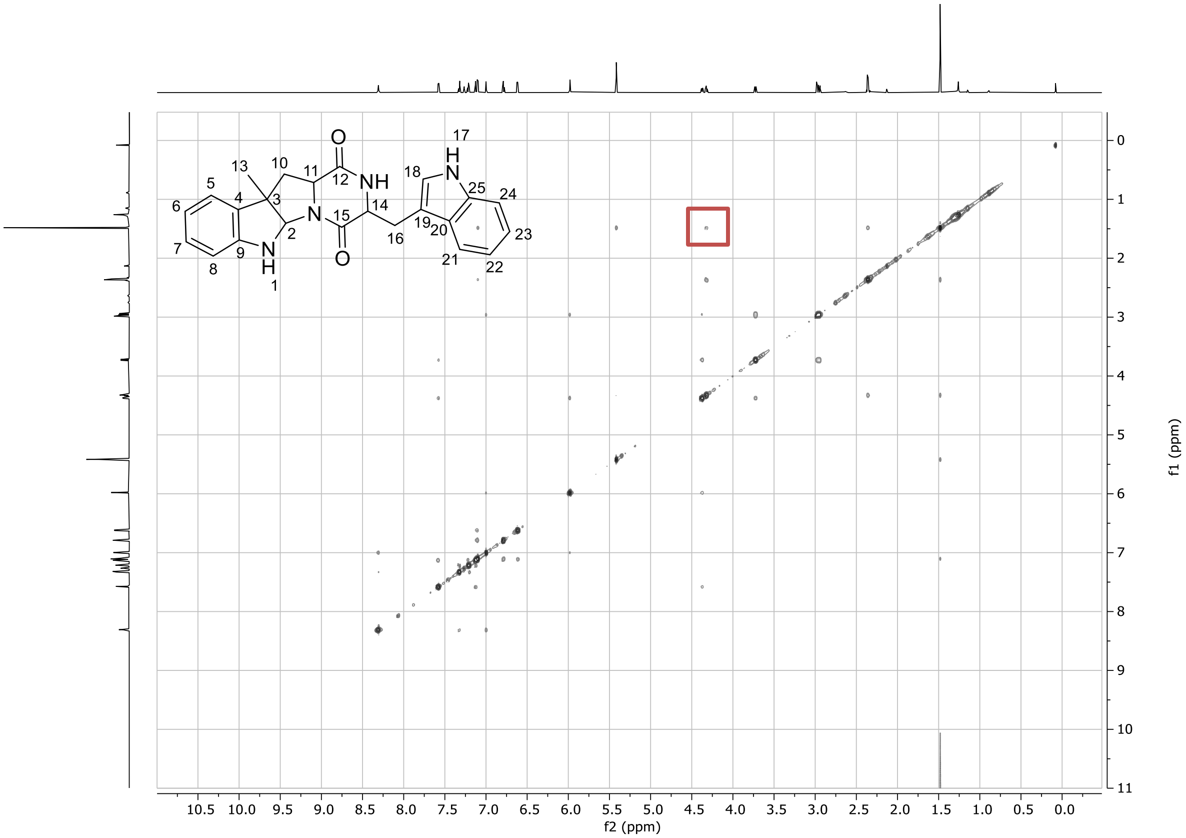

Figure S46. ROESY-spectrum of single methylated *DD*-cWW **16** in $CDCl_3$ (750 MHz).

References

(1) Jumper, J.; Evans, R.; Pritzel, A.; Green, T.; Figurnov, M.; Ronneberger, O.; Tunyasuvunakool, K.; Bates, R.; Žídek, A.; Potapenko, A. Highly accurate protein structure prediction with AlphaFold. *Nature*, 2021, 583-589. https://doi.org/10.1038/s41586-021-03819-2.
(2) Skolnick, J.; Gao, M.; Zhou, H.; Singh, S. AlphaFold 2: why it works and its implications for understanding the relationships of protein sequence, structure, and function. *J. Chem. Inf. Model.*, 2021, 4827-4831. https://doi.org/10.1021/acs.jcim.1c01114.
(3) Baek, M.; Park, T.; Heo, L.; Park, C.; Seok, C. GalaxyHomomer: a web server for protein homo-oligomer structure prediction from a monomer sequence or structure. *Nucleic Acids Res.*, 2017, W320-W324. https://doi.org/10.1093/nar/gkx246.
(4) Heo, L.; Park, H.; Seok, C. GalaxyRefine: Protein structure refinement driven by side-chain repacking. *Nucleic Acids Res.*, 2013, W384-W388. https://doi.org/10.1093/nar/gkt458.
(5) Amariei, D. A.; Pozhydaieva, N.; David, B.; Schneider, P.; Classen, T.; Gohlke, H.; Weiergräber, O. H.; Pietruszka, J. Enzymatic C3-Methylation of Indoles Using Methyltransferase PsmD— Crystal Structure, Catalytic Mechanism, and Preparative Applications. *ACS Catal.*, 2022, 14130-14139. https://doi.org/10.1021/acscatal.2c04240.
(6) Friesner, R. A.; Banks, J. L.; Murphy, R. B.; Halgren, T. A.; Klicic, J. J.; Mainz, D. T.; Repasky, M. P.; Knoll, E. H.; Shelley, M.; Perry, J. K. Glide: a new approach for rapid, accurate docking and scoring. 1. Method and assessment of docking accuracy. *J. Med. Chem.*, 2004, 1739-1749. https://doi.org/10.1021/jm0306430.
(7) Halgren, T. A.; Murphy, R. B.; Friesner, R. A.; Beard, H. S.; Frye, L. L.; Pollard, W. T.; Banks, J. L. Glide: a new approach for rapid, accurate docking and scoring. 2. Enrichment factors in database screening. *J. Med. Chem.*, 2004, 1750-1759. https://doi.org/10.1021/jm030644s.
(8) Harder, E.; Damm, W.; Maple, J.; Wu, C.; Reboul, M.; Xiang, J. Y.; Wang, L.; Lupyan, D.; Dahlgren, M. K.; Knight, J. L. OPLS3: a force field providing broad coverage of drug-like small molecules and proteins. *J. Chem. Theory Comput.*, 2016, 281-296. https://doi.org/10.1021/acs.jctc.5b00864.
(9) Madhavi Sastry, G.; Adzhigirey, M.; Day, T.; Annabhimoju, R.; Sherman, W. Protein and ligand preparation: parameters, protocols, and influence on virtual screening enrichments. *J. Comput. Aided Mol. Des.*, 2013, 221-234. https://doi.org/10.1007/s10822-013-9644-8.
(10) Zhang, J.; Kulik, H. J.; Martinez, T. J.; Klinman, J. P. Mediation of donor–acceptor distance in an enzymatic methyl transfer reaction. *PNAS*, 2015, 7954-7959. https://doi.org/10.1073/pnas.1506792112.
(11) Bayly, C.; Cieplak, P.; Cornell, W.; Kollman, P. A well-behaved electrostatic potential based method using charge restraints for deriving. *J. Phys. Chem.*, 1993, 10269-10280. https://doi.org/10.1021/j100142a004.
(12) Lu, T.; Chen, F. Multiwfn: a multifunctional wavefunction analyzer. *J. Comput. Chem.*, 2012, 580-592. https://doi.org/10.1002/jcc.22885.
(13) Neese, F.; Wennmohs, F.; Becker, U.; Riplinger, C. The ORCA quantum chemistry program package. *J. Chem. Phys.*, 2020, 224108. https://doi.org/10.1063/5.0004608.
(14) Wang, J.; Wolf, R. M.; Caldwell, J. W.; Kollman, P. A.; Case, D. A. Development and testing of a general amber force field. *J. Comput. Chem.*, 2004, 1157-1174. https://doi.org/10.1002/jcc.20035.

(15) Wang, J.; Wang, W.; Kollman, P. A.; Case, D. A. Automatic atom type and bond type perception in molecular mechanical calculations. *J. Mol. Graph. Model.*, 2006, 247-260. https://doi.org/10.1016/j.jmgm.2005.12.005.
(16) Maier, J. A.; Martinez, C.; Kasavajhala, K.; Wickstrom, L.; Hauser, K. E.; Simmerling, C. ff14SB: improving the accuracy of protein side chain and backbone parameters from ff99SB. *J. Chem. Theory Comput.*, 2015, 3696-3713. https://doi.org/10.1021/acs.jctc.5b00255.
(17) Jorgensen, W. L.; Chandrasekhar, J.; Madura, J. D.; Impey, R. W.; Klein, M. L. Comparison of simple potential functions for simulating liquid water. *J. Chem. Phys.*, 1983, 926-935. https://doi.org/10.1063/1.445869.
(18) Olsson, M. H.; Søndergaard, C. R.; Rostkowski, M.; Jensen, J. H. PROPKA3: consistent treatment of internal and surface residues in empirical p K a predictions. *J. Chem. Theory Comput.*, 2011, 525-537. https://doi.org/10.1021/ct100578z.
(19) Giambasu, G. M.; Case, D. A.; York, D. M. Predicting site-binding modes of ions and water to nucleic acids using molecular solvation theory. *J. Am. Chem. Soc.*, 2019, 2435-2445. https://doi.org/10.1021/jacs.8b11474.
(20) Giambaşu, G. M.; Gebala, M. K.; Panteva, M. T.; Luchko, T.; Case, D. A.; York, D. M. Competitive interaction of monovalent cations with DNA from 3D-RISM. *Nucleic Acids Res.*, 2015, 8405-8415. https://doi.org/10.1093/nar/gkv830.
(21) Giambaşu, G. M.; Luchko, T.; Herschlag, D.; York, D. M.; Case, D. A. Ion counting from explicit-solvent simulations and 3D-RISM. *Biophys. J.*, 2014, 883-894. http://hdl.handle.net/10211.3/196250.
(22) Beglov, D.; Roux, B. An integral equation to describe the solvation of polar molecules in liquid water. *J. Phys. Chem.*, 1997, 7821-7826. https://doi.org/10.1021/jp971083h.
(23) Kovalenko, A.; Hirata, F. Three-dimensional density profiles of water in contact with a solute of arbitrary shape: a RISM approach. *Chem. Phys. Lett.*, 1998, 237-244. https://doi.org/10.1016/S0009-2614(98)00471-0.
(24) Darden, T.; York, D.; Pedersen, L. Particle mesh Ewald: An N· log (N) method for Ewald sums in large systems. *J. Chem. Phys.*, 1993, 10089-10092. https://doi.org/10.1063/1.464397.
(25) Case, D. A.; Aktulga, H. M.; Belfon, K.; Ben-Shalom, I.; Brozell, S. R.; Cerutti, D. S.; Cheatham III, T. E.; Cruzeiro, V. W. D.; Darden, T. A.; Duke, R. E. *Amber 2021*; University of California, San Francisco, 2021.
(26) Salomon-Ferrer, R.; Gotz, A. W.; Poole, D.; Le Grand, S.; Walker, R. C. Routine microsecond molecular dynamics simulations with AMBER on GPUs. 2. Explicit solvent particle mesh Ewald. *J. Chem. Theory Comput.*, 2013, 3878-3888. https://doi.org/10.1021/ct400314y.
(27) Liu, J.; Li, D.; Liu, X. A simple and accurate algorithm for path integral molecular dynamics with the Langevin thermostat. *J. Chem. Phys.*, 2016, 024103. https://doi.org/10.1063/1.4954990.
(28) Ryckaert, J.-P.; Ciccotti, G.; Berendsen, H. J. Numerical integration of the cartesian equations of motion of a system with constraints: molecular dynamics of n-alkanes. *J. Comput. Phys.*, 1977, 327-341. https://doi.org/10.1016/0021-9991(77)90098-5.
(29) Roe, D. R.; Cheatham III, T. E. PTRAJ and CPPTRAJ: software for processing and analysis of molecular dynamics trajectory data. *J. Chem. Theory Comput.*, 2013, 3084-3095. https://doi.org/10.1021/ct400341p.

(30) Zhang, X.; Zhou, L.; Cheng, X. Crystal structure of the conserved core of protein arginine methyltransferase PRMT3. *EMBO J.*, 2000, 3509-3519. https://doi.org/10.1093/emboj/19.14.3509.
(31) Wu, R.; Cao, Z. QM/MM study of catalytic methyl transfer by the N5-glutamine SAM-dependent methyltransferase and its inhibition by the nitrogen analogue of coenzyme. *J. Comput. Chem.*, 2008, 350-357. https://doi.org/10.1002/jcc.20793.
(32) Sharp, K. A.; Honig, B. Calculating total electrostatic energies with the nonlinear Poisson-Boltzmann equation. *J. Phys. Chem.*, 1990, 7684-7692. https://doi.org/10.1021/j100382a068.
(33) Gohlke, H.; Case, D. A. Converging free energy estimates: MM-PB (GB) SA studies on the protein–protein complex Ras–Raf. *J. Comput. Chem.*, 2004, 238-250. https://doi.org/10.1002/jcc.10379.
(34) Onufriev, A.; Bashford, D.; Case, D. A. Modification of the generalized Born model suitable for macromolecules. *J. Phys. Chem.*, 2000, 3712-3720. https://doi.org/10.1021/jp994072s.
(35) Miller III, B. R.; McGee Jr, T. D.; Swails, J. M.; Homeyer, N.; Gohlke, H.; Roitberg, A. E. MMPBSA. py: an efficient program for end-state free energy calculations. *J. Chem. Theory Comput.*, 2012, 3314-3321. https://doi.org/10.1021/ct300418h.
(36) Crooks, G. E.; Hon, G.; Chandonia, J.-M.; Brenner, S. E. WebLogo: a sequence logo generator. *Genome Res.*, 2004, 1188-1190. http://www.genome.org/cgi/doi/10.1101/gr.849004.
(37) Schneider, T. D.; Stephens, R. M. Sequence logos: a new way to display consensus sequences. *Nucleic Acids Res.*, 1990, 6097-6100. https://doi.org/10.1093/nar/18.20.6097.
(38) Katoh, K.; Standley, D. M. MAFFT multiple sequence alignment software version 7: improvements in performance and usability. *Mol. Biol. Evol.*, 2013, 772-780. https://doi.org/10.1093/molbev/mst010.
(39) Berezin, C.; Glaser, F.; Rosenberg, J.; Paz, I.; Pupko, T.; Fariselli, P.; Casadio, R.; Ben-Tal, N. ConSeq: the identification of functionally and structurally important residues in protein sequences. *Bioinform.*, 2004, 1322-1324. https://doi.org/10.1093/bioinformatics/bth070.
(40) Ashkenazy, H.; Abadi, S.; Martz, E.; Chay, O.; Mayrose, I.; Pupko, T.; Ben-Tal, N. ConSurf 2016: an improved methodology to estimate and visualize evolutionary conservation in macromolecules. *Nucleic Acids Res.*, 2016, W344-W350. https://doi.org/10.1093/nar/gkw408.
(41) Suzek, B. E.; Huang, H.; McGarvey, P.; Mazumder, R.; Wu, C. H. UniRef: comprehensive and non-redundant UniProt reference clusters. *Bioinform.*, 2007, 1282-1288. https://doi.org/10.1093/bioinformatics/btm098.
(42) Altschul, S. F.; Madden, T. L.; Schäffer, A. A.; Zhang, J.; Zhang, Z.; Miller, W.; Lipman, D. J. Gapped BLAST and PSI-BLAST: a new generation of protein database search programs. *Nucleic Acids Res.*, 1997, 3389-3402. https://doi.org/10.1093/nar/25.17.3389.
(43) He, B.; Mortuza, S.; Wang, Y.; Shen, H.-B.; Zhang, Y. NeBcon: protein contact map prediction using neural network training coupled with naïve Bayes classifiers. *Bioinform.*, 2017, 2296-2306. https://doi.org/10.1093/bioinformatics/btx164.
(44) Alqahtani, N.; Porwal, S. K.; James, E. D.; Bis, D. M.; Karty, J. A.; Lane, A. L.; Viswanathan, R. Synergism between genome sequencing, tandem mass spectrometry and bio-inspired synthesis reveals insights into nocardioazine B biogenesis. *OBC*, 2015, 7177-7192. https://doi.org/10.1039/C5OB00537J.
(45) Schneider, P.; Henßen, B.; Paschold, B.; Chapple, B. P.; Schatton, M.; Seebeck, F. P.; Classen, T.; Pietruszka, J. Biocatalytic C3-Indole Methylation—A Useful Tool for the Natural-

Product-Inspired Stereoselective Synthesis of Pyrroloindoles. *Angew. Chem. Int.*, 2021, 23412-23418. https://doi.org/10.1002/anie.202107619.
(46) Yi, J. C.; Liu, C.; Dai, L. X.; You, S. L. Synthesis of C3-Methyl-Substituted Pyrroloindolines and Furoindolines via Cascade Dearomatization of Indole Derivatives with Methyl Iodide. *Chem. Asian J.*, 2017, 2975-2979. https://doi.org/10.1002/asia.201701151.
(47) Li, H.; Qiu, Y.; Guo, C.; Han, M.; Zhou, Y.; Feng, Y.; Luo, S.; Tong, Y.; Zheng, G.; Zhu, S. Pyrroloindoline cyclization in tryptophan-containing cyclodipeptides mediated by an unprecedented indole C3 methyltransferase from Streptomyces sp. HPH0547. *ChemComm*, 2019, 8390-8393. https://doi.org/10.1039/C9CC03745D.
